# Identification of HOC^•^HC(O)H, HOCH_2_C^•^O, and HOCH_2_CH_2_O^•^ Intermediates in the Reaction of H + Glycolaldehyde in Solid *Para*-Hydrogen and Its Implication to the Interstellar Formation of Complex Sugars

**DOI:** 10.1021/jacs.4c05896

**Published:** 2024-08-09

**Authors:** Prasad Ramesh Joshi, Yuan-Pern Lee

**Affiliations:** † Department of Applied Chemistry and Institute of Molecular Science, National Yang Ming Chiao Tung University, Hsinchu 300093, Taiwan; ‡ Center for Emergent Functional Matter Science, National Yang Ming Chiao Tung University, Hsinchu 300093, Taiwan

## Abstract

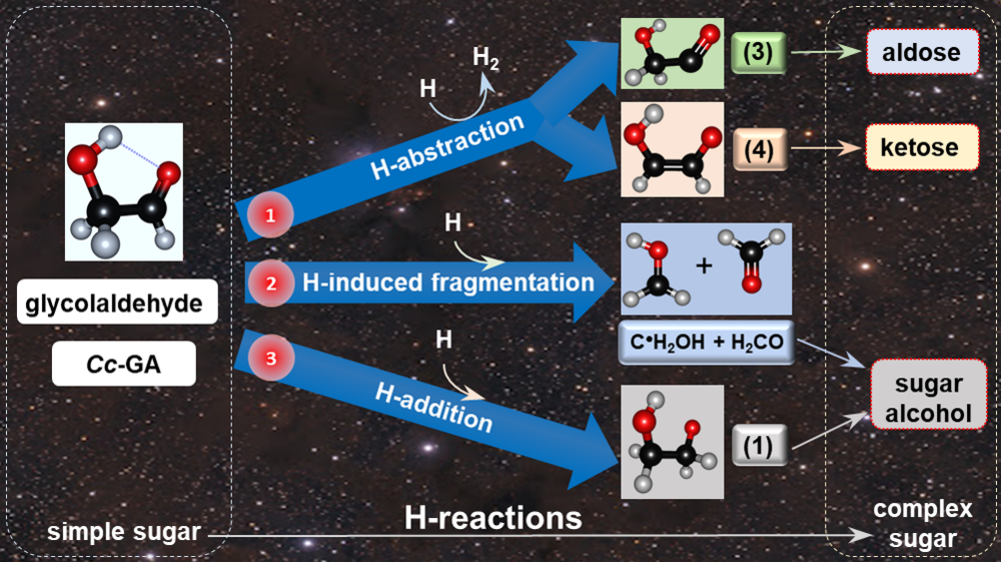

Glycolaldehyde [HOCH_2_C(O)H, GA], the primitive sugar-like molecule detected in the interstellar medium (ISM), is a potential precursor for the synthesis of complex sugars. Despite its importance, the mechanism governing the formation of these higher-order sugars from GA under interstellar circumstances remains elusive. Radical intermediates HOCH_2_CH_2_O^•^ (**1**), HOCH_2_C^•^HOH (**2**), HOCH_2_C^•^O (**3**), HOC^•^HC(O)H (**4**), and O^•^CH_2_C(O)H (**5**) derived from GA could be potential precursors for the formation of glyceraldehyde (aldose sugar), dihydroxyacetone (ketose sugar), and ethylene glycol (sugar alcohol) in dark regions of ISM. However, the spectral identification of these intermediates and their roles were little investigated. We conducted reactions involving H atoms and the *Cis*-*cis* conformer of GA (*Cc*-GA) in solid *p*-H_2_ at 3.2 K and identified IR spectra of radicals *Cc*-HOCH_2_C^•^O (**3**) and *Cc*-HOC^•^HC(O)H (**4**) produced from H abstraction as well as closed-shell HOCHCO (**6**) produced via consecutive H abstraction of GA. In addition, *Cc*-HOCH_2_CH_2_O^•^ (**1**) and C^•^H_2_OH + H_2_CO (**7**) were produced through the H addition and the H-induced fragmentation channels, respectively. In darkness, when only H-tunneling reactions occurred, the formation of (**3**) was major and that of (**1**) was minor. In contrast, during IR irradiation to produce H atoms with higher energy, the formation of (**4**) and C^•^H_2_OH + H_2_CO (**7**) became important. We also successfully converted most *Cc-*GA to the second-lowest-energy conformer *Trans*-*trans*-GA (*Tt*-GA) by prolonged IR irradiation at 2827 nm to investigate H + *Tt*-GA; *Tt*-HOCH_2_C^•^O (**3′**), *Tt*-HOC^•^HC(O)H (**4′),** HOCHCO (**6**), *Tt*-HOCH_2_CH_2_O^•^ (**1′**), and C^•^H_2_OH + H_2_CO (**7**) were observed. We discuss possible routes for the formation of higher-order sugars or related compounds involving (**7**), (**1**), (**3**), and (**4**), but neither (**2**), which was proposed previously, nor (**5**) plays a significant role in H + GA. Such previously unreported rich chemistry in the reaction of H + GA, with four channels of three distinct types, indicates the multiple roles that GA might play in astronomical chemistry.

## Introduction

The complex organic molecules (COM) have received a lot of attention for their potential contribution to the origin of life, which involves the formation of amino acids, sugars, and nucleobases. Sugars are key ingredients in astrobiology because of their active roles in the origin of life via the RNA hypothesis; they also play a significant role in the biological processes such as metabolism and transmission of genetic information.^1−3^ Glycolaldehyde (hydroxyacetaldehyde or hydroxyethanal, HOCH_2_C(O)H, denoted GA) is the first sugar-like molecule detected in the interstellar medium (ISM). It was first revealed toward the galactic center source Sgr B2(N) in 2000,^4^ and confirmed by several observations.^5−9^ Later, GA was detected toward the center of our galaxy,^10^ hot molecular cores of star-forming regions,^11−15^ solar-like protostars,^16−19^ and cometary ices.^20−22^ GA is the smallest possible molecule to contain both an aldehyde moiety and a hydroxy moiety; it also conforms to the general formula C_n_(H_2_O)_n_ for carbohydrate. GA plays a catalytic role in the formose reaction,^23^ which involves the formation of sugars from formaldehyde (H_2_CO); the reaction of GA with H_2_CO produces glyceraldehyde, a triose; subsequent additions of H_2_CO lead to the facile production of ribose, a crucial component of RNA.^24,25^ Cometary GA was proposed as a source of pre-RNA molecules.^26^

GA was also formed in significant yields in the atmospheric oxidation of ethene^27^ or isoprene.^28^ The most important degradation paths of GA are its reaction with OH (major) and photolysis (minor). In both cases, the main path is assumed to involve the fission of the C–H bond of the formyl moiety [C(O)H] to yield hydroxyacetyl radical, HOCH_2_C^•^O (**3**).^29^ Niki et al. investigated reactions of Cl + GA and OH + GA at 700 Torr with infrared (IR) absorption and, from indirect evidence, reported that the major initial path is the formation of the HOCH_2_C^•^O (**3**) radical, whereas the minor path is the formation of the hydroxy-vinoxy [or 1-hydroxy-2-oxo ethyl, HOC^•^HC(O)H (**4**)] from the H abstraction of the CH_2_ moiety.^30^ However, neither radical was identified directly.

Álvarez-Barcia et al. calculated rate coefficients (*k*) of possible reactions of H + GA at 75 K with the density-functional theory MPWB1K/def2-TZVP and the activation energy (*E*_a_) of each channel with the CCSD(T)-F12/VTZ-F12 method,^31^
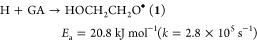
1
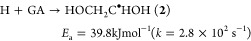
2
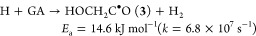
3
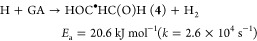
4
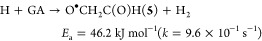
5in which reactions 1 and 2 involve the H addition to the C atom and the O atom of the C(O)H moiety of GA, respectively, whereas reactions 3–5 involve the H abstraction of the C(O)H, CH_2_, and OH moieties of GA, respectively. Further H-abstraction on the CH_2_ or C(O)H moieties of HOCH_2_C^•^O (**3**) or HOC^•^HC(O)H (**4**), respectively, may result in the formation of the closed-shell molecule hydroxyketene (or 2-hydroxyethen-1-one, HOCHCO) (**6**), one of the products identified in this work.
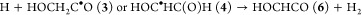
6

**Figure 1:**
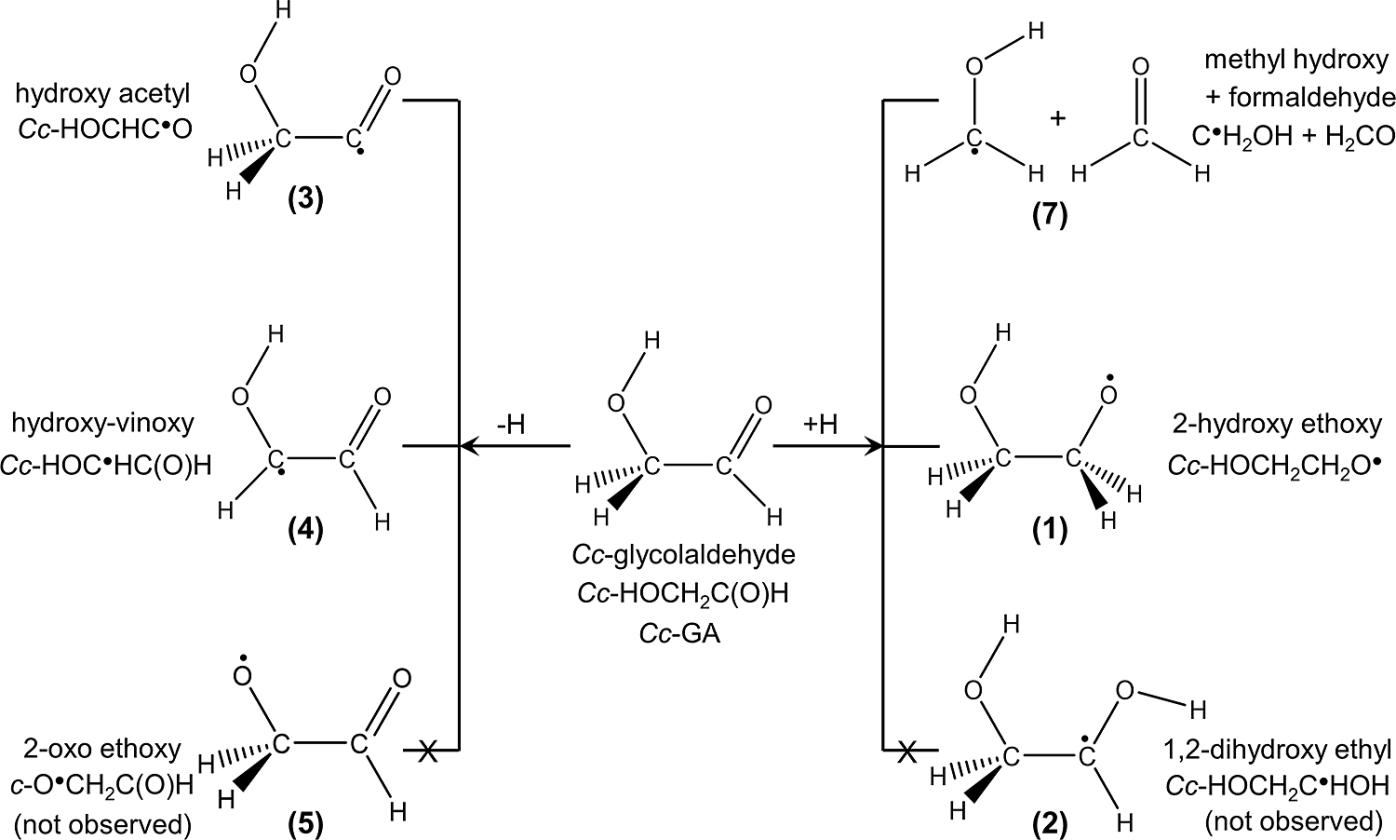
Scheme for the reactions of *Cc*-glycolaldehyde with hydrogen atoms.

Reactions 1–5 suggested by Álvarez-Barcia et al. and the overlooked H-induced fragmentation,
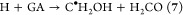
7are summarized in the scheme in Figure 1. None of these radicals produced in reactions 1–5 have been identified directly in either gas-phase or solid-state experimental studies, nor their further reactions explored. Detecting the radicals and understanding their reactions will provide valuable information on the reactions of GA and the formation of sugars in the present astrochemical model.

Fedoseev et al. observed the formation of GA and ethylene glycol (HOCH_2_CH_2_OH, EG) by hydrogenation of CO under dense-cloud conditions.^32^ Leroux et al. bombarded GA ice at 10 K with H atoms and observed the formation of EG, indicating a chemical link between these two compounds in ISM via H addition,^33^
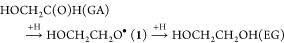
8However, these authors stated that the reverse reactions
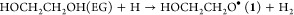
9
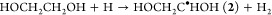
10have large activation energies, calculated to be 54 and 28 kJ mol^–1^, respectively,^31^ so that these reactions would be inefficient under their experimental conditions. Rivilla et al. reported that the abundance ratios of [EG]/[GA] in the star-forming regions varies with the luminosity of the source, spanning from 1 to >15, which indicates that likely EG and GA are formed by different chemical routes and are not directly linked.^13^ Detection of either (**1**) or (**2**) will help us to understand the link between GA and EG.

Fedoseev et al.^34^ performed solid-state hydrogenation experiments and demonstrated the formation of glycerol (a sugar alcohol with three carbons) and, tentatively, glyceraldehyde (a sugar) by codepositing GA, CO, and H atoms at 15 K; glycerol is critical for the formation of membranes of living cells and organelles. These authors proposed a reaction mechanism involving the hydrogenation of GA to form the intermediate 1,2-dihydroxy ethyl radical HOCH_2_C^•^HOH (**2**) via reaction 2, followed by subsequent reactions with C^•^H_2_OH or HC^•^O to form glycerol or glyceraldehyde, respectively; this scheme might be extended similarly to form more complex sugars and sugar alcohols. The intermediate HOCH_2_C^•^HOH (**2**) proposed from the H addition of GA in this scheme is different from that, 2-hydroxy ethoxy radical HOCH_2_CH_2_O^•^ (**1**), proposed in the scheme for converting GA to EG by Leroux et al.^33^ Furthermore, two H-abstraction reactions, producing HOCH_2_C^•^O (**3**) and HOC^•^HC(O)H (**4**) via reactions 3 and 4, respectively, were predicted to have barriers smaller than that of reaction 1, 21 kJ mol^–1^. Reactions 3 and 4 might play important roles in the reaction of GA with H atoms and the formation of glycerol and glyceraldehyde.

To investigate reactive interstellar intermediates at low temperatures, we have successfully employed the *p*-H_2_ quantum solid as a matrix host to take advantage of its unique properties, such as diminished cage effect, convenient generation of hydrogen atoms, and efficient quantum-tunneling of H atoms to study reactions of H atoms with astrochemically relevant species.^35^ Although solid *p*-H_2_ does not mimic closely astrochemical environments, it offers excellent opportunities to investigate fundamental reaction mechanisms involved in the formation of COM, particularly in dark regions of ISM. The reactions of H atoms with astrochemically relevant species including methanol,^36^ formamide,^37^ methyl formate,^38^ acetamide,^39^ acetic acid,^40^ glycine,^41^ methyl amine,^42^ and *N*-methyl formamide^43^ were explored in our previous investigations; the reactions of H atoms with fulminic acid, formaldoxime,^44^ and ethanol^45^ were studied by Tarczay and co-workers. Many of these reactions proceed through quantum tunneling when they involve a small barrier. The H atoms are mobile in solid *p-*H_2_ because of the quantum diffusion, in which the H atoms diffuse “chemically” by reacting with their neighboring H_2_ molecules through breaking the existing H–H bond and forming a new H–H bond and a “diffused” H atom via quantum-tunneling; successive formation and breaking of H–H bonds allow the hydrogen atom to move in solid *p-*H_2_ and eventually reach the vicinity of guest molecules to react.^46^ The previously overlooked H-abstraction channels and the coupling of H abstraction and H addition to induce either a quasi-equilibrium between two species, such as formamide H_2_NC(O)H and HNCO,^37^ or an endothermic reaction, such as the isomerization of *trans*-NMF (*N*-methyl formamide) to *cis*-NMF with higher energy or the H-induced fragmentation, such as the formation of HNCO + CH_4_ and CH_2_NH + CO from H + *trans*-NMF, have been reported.^43^ A detailed description on these H atom reactions in solid *p*-H_2_ is available in a recent review.^35^

In this work, we investigated the reactions of H atoms with two lowest-energy conformers (*Cc*- and *Tt*-) of GA and identified radicals HOCH_2_C^•^O (**3**) and HOC^•^HC(O)H (**4**) via H abstraction, HOCH_2_CH_2_O^•^ (**1**) via H addition, and C^•^H_2_OH + H_2_CO (**7**) via H-induced fragmentation. IR absorption spectra of the *Cc*- and *Tt*-forms of (**3**), (**4**), and (**1**) were clearly characterized. Further H abstraction yielded HOCHCO (**6**). The roles of these radicals in the production of aldose, ketose, and EG are discussed.

## Results

**Figure 2:**
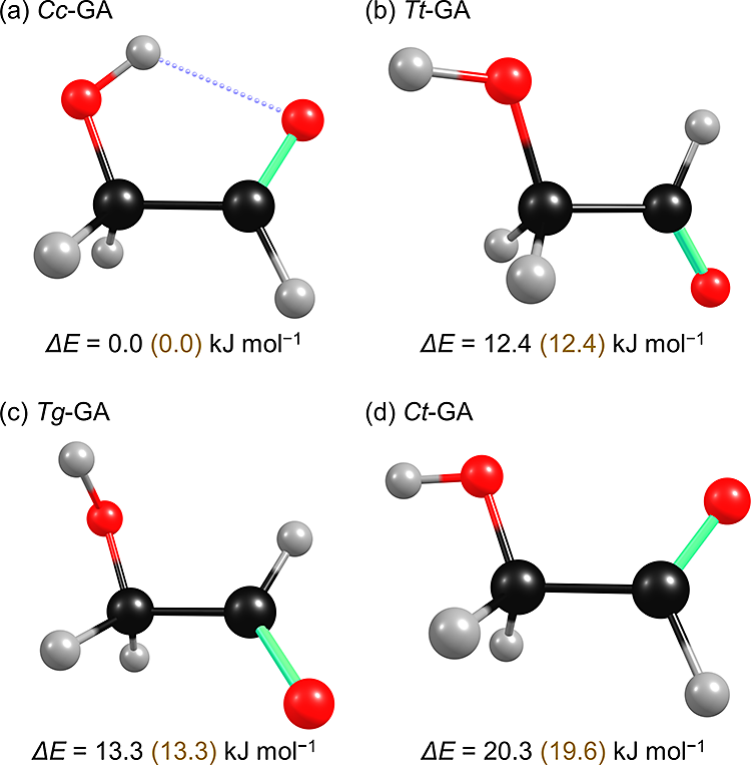
Geometries of conformers of glycolaldehyde (GA) optimized with the B3LYP/aug-cc-pVTZ method. (a) *Cis–cis* glycolaldehyde (*Cc*-GA), (b) *Trans–trans* glycolaldehyde (*Tt*-GA), (c) *Trans–gauche* glycolaldehyde (*Tg*-GA), and (d) *Cis–trans* glycolaldehyde (*Ct*-GA). Relative energies (in kJ mol^–1^) calculated with the CCSD(T)/aug-cc-pVTZ//B3LYP/aug-cc-pVTZ method (black) are listed; those with the B3LYP/aug-cc-pVTZ method (olive) are given in parentheses for comparison.

GA has 4 possible conformers, *Cc*, *Tt*, *Tg*, and *Ct*, in which the first capital character refers to the relative orientation of the C=O and the C–O bonds and the second character the O–H and the C–C bonds; *c*, *t*, and *g* refer to *cis*, *trans*, and *gauche* conformations, respectively.^47^ Geometries of these four conformers and relative energies are presented in Figure 2; structural parameters of these conformers are depicted in Figure S1. The *Cc*-GA with an intramolecular hydrogen bond (H-bond) has the lowest energy and is dominant in the gaseous phase.^48,49^ In matrices, some *Cc*-GA were reported to be converted to *Tt*-GA upon IR irradiation.^50^

**Figure 3:**
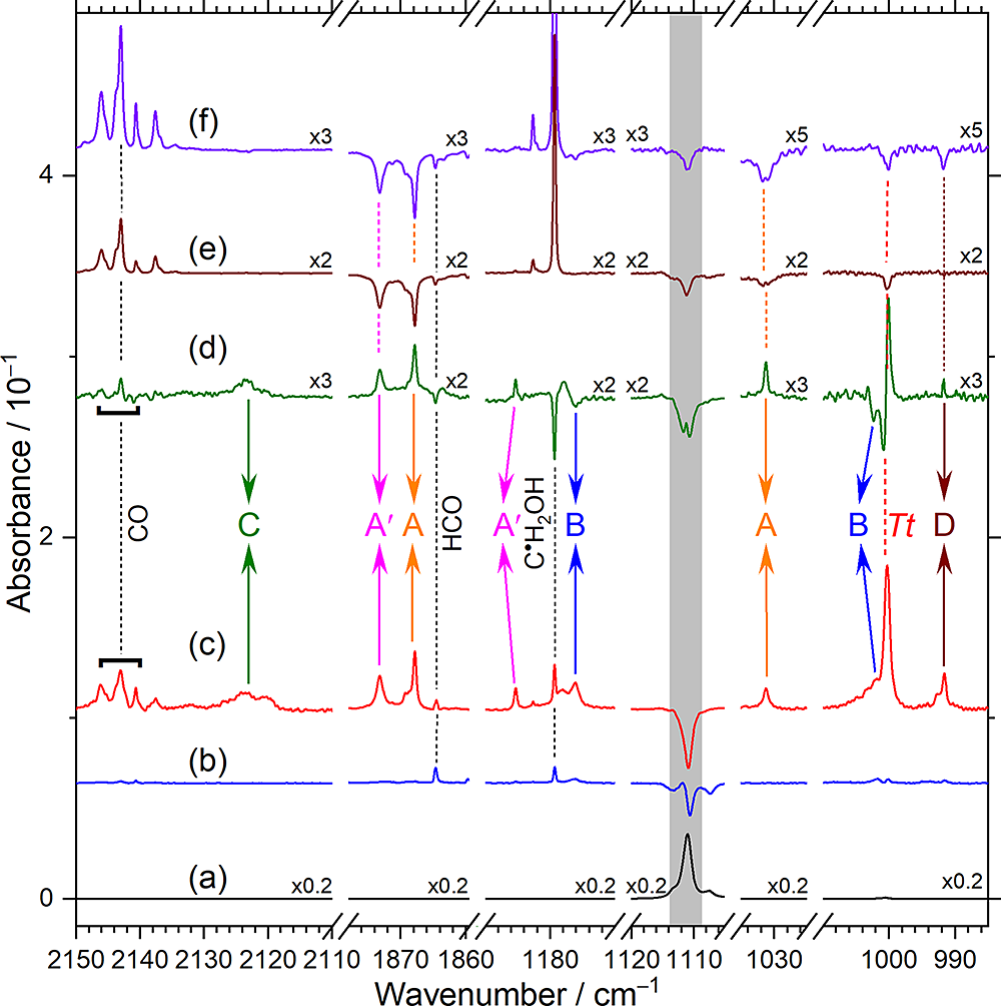
Representative spectra of a GA/Cl_2_/*p*-H_2_ (1/10/10,000) matrix recorded at various stages of the H + *Cc*-GA experiment. (a) Spectrum recorded after deposition at 3.2 K for 6 h. Difference spectra after photolysis at 380 nm for 30 min (b), after additional full IR irradiation for 1 h (c), after terminating the IR irradiation and maintaining the matrix in darkness for 10 h (d), after secondary photolysis at 520 nm for 15 min (e), and after further secondary photolysis at 460 nm for 15 min (f). Lines in groups A–D and A′ are marked with orange, blue, green, brown, and pink arrows and labels; these lines are assigned as *Cc*-HOCH_2_C^•^O (**3**), *Cc*-HOC^•^HC(O)H (**4**), HOCHCO (**6**), *Cc*-HOCH_2_CH_2_O^•^ (**1**), and *Tt*-HOCH_2_C^•^O (**3′**), respectively. Lines of *Tt*-GA are indicated with red. The shaded gray area indicates spectral regions subjected to interference by the absorption of *Cc*-GA.

The IR spectrum in representative spectral regions after deposition of a GA/Cl_2_/*p*-H_2_ (1/10/10,000) matrix for 6 h at 3.2 K is depicted in Figure 3a; the full-range spectrum (except 2750–2250 cm^–1^) is presented in Figure S2a. Most lines correspond to the most stable *Cc*-GA (∼97%), and some to *Tt-*GA (∼3%), with wavenumbers nearly identical to those reported previously for *Cc*-GA and *Tt*-GA in *p*-H_2_;^51,52^ lines due to *Tt*-GA are indicated in red in the figures. The observed wavenumbers are compared with the literature values and the scaled harmonic vibrational wavenumbers predicted with the B3LYP/aug-cc-pVTZ method in Table S1; the harmonic vibrational wavenumbers are scaled according to 0.9619*x* + 24.6 for wavenumbers <2500 cm^–1^ and 0.9200*x* + 133.4 for wavenumbers >2500 cm^–1^, as discussed in the Methods section.

The matrix was photolyzed at 380 nm for 30 min to generate Cl atoms, and the difference spectra after UV irradiation are depicted in Figures 3b and S2b; the difference spectrum was obtained on subtracting the spectrum recorded before a specific experimental step from that recoded afterward. Because a small amount of GA was dissociated near 365 nm, a typical wavelength used to generate Cl atoms, we chose the longest feasible wavelength to minimize the photolysis as much as possible while maintaining sufficient production of Cl atoms. Even at this wavelength, a small amount of C^•^H_2_OH (∼1.4 ppm),^53^ H_2_CO (∼1.2 ppm),^52^ HC^•^O (∼1.0 ppm),^52^ and *Tt*-conformer (0.3 ppm)^52^ were observed; values in parentheses are estimated mixing ratios according to theoretical IR intensities. Subsequent IR irradiation was performed to generate H atoms (along with HCl) through the reaction Cl + H_2_ (ν = 1) → HCl + H; the resultant difference spectra are presented in Figures 2c and S2c, in which positive features indicate production and negative ones destruction. Apart from the newly observed features, which will be discussed later, absorption attributed to HCl (from Cl + H_2_)^54^ and HO_2_ (from H + O_2_, which are representative indications of the presence of H atoms in the matrix)^55^ were observed. After IR irradiation, the matrix was maintained in darkness for 10 h to allow H atom tunneling reactions; the difference spectra are presented in Figures 2d and S2d. Subsequently, the matrix was subjected to secondary photolysis at various wavelengths to differentiate the newly observed lines; the difference spectra following irradiation at 520 and 460 nm are presented in Figures 2e/S2e and 2f/S2f, respectively.

Upon IR irradiation (Figures 3c and S2c), a set of new features (group A) appeared at 3626.5, 1867.9, 1425.0, 1215.6, 1054.6(?), 1031.4, and 677.9 cm^–1^; the question mark indicates uncertainty in grouping, likely due to interference by other species. These features increased in intensity by ∼32% after maintenance in darkness and decreased in intensity by ∼65 and ∼30% upon secondary photolysis at 520 and 460 nm, respectively. As discussed later, they are assigned to *Cc*-HOCH_2_C^•^O (**3**). A second set of new lines, group B, appeared at 3371.8, 1549.5, 1493.2, 1308.8, 1176.3, 1002.4, 760.9, and 793.5 cm^–1^. They decreased in intensity by ∼15% after being maintained in darkness and remained unaltered after secondary irradiation at 520 and 460 nm. As discussed later, they are assigned to *Cc*-HOC^•^HC(O)H (**4**). The third set, group C, appeared at 3617.0, 3033.2, 2123.6, 1250.2, and 1137.4(?) cm^–1^ after IR irradiation, increased by ∼21% in darkness, and remained the same upon secondary photolysis; they are later assigned to HOCHCO (**6**). Finally, new lines in group D appeared at 3610.5, 2905.5(?), 1354.8, 1071.4, 991.7, and 765.2 cm^–1^ after IR irradiation, slightly increased in intensity by ∼6% after being in darkness, remained unchanged upon 520 nm irradiation, and decreased in intensity by ∼11% upon 460 nm irradiation; they are later assigned to *Cc*-HOCH_2_CH_2_O^•^ (**1**). In addition to these four groups, four intense lines at 3677.3, 1204.4, 1067.7, and 1000.2 cm^–1^ and some weaker ones of *Tt*-GA were enhanced after IR irradiation.^52^ The *Cc* → *Tt* conformational conversion was also reported by Aspiala et al. when they employed a broadband IR source to irradiate *Cc*-GA in various rare-gas matrices.^50^ The variation of integrated absorbance for *Cc*-GA, *Tt*-GA, and four groups of new lines after each step of the experiment is summarized in Table S2. In this H-rich reaction, we estimated the mixing ratios of *Cc*-GA and *Tt*-GA to be ∼89 and ∼36 ppm, respectively, after IR irradiation; thus, H atoms likely reacted with both conformers of GA to form the observed products.

To distinguish the reactions of *Cc*-GA and *Tt*-GA, in one type of experiment, we employed IR irradiation at 3537 cm^–1^ (2827 nm), corresponding to the OH-stretching fundamental of *Cc*-GA, to convert *Cc*-GA to *Tt-GA* as much as possible before investigating its reaction with H atoms, indicated as H + *Tt-*GA hereafter. The conformation conversion with IR irradiation is preferred over the UV irradiation at 266 nm because the latter also causes fragmentation,^52^ as compared in Figure S3; IR irradiation at 2827 nm of *Cc*-GA in solid *p*-H_2_ for 15 h yielded ∼89% of *Tt*-GA and <1% *Tg*-GA,^56^ without any fragmented products, as represented in Figure S3c.

The results of H + *Tt-*GA are presented in Figures S4 and S5. Three groups of observed features, Groups A′, B′, and D′, with behavior similar to those of groups A, B, and D in experiments of H + *Cc-*GA, respectively, and lines in group C with unshifted lines were observed. The wavenumber lists of groups A′, B′, and D′ are also presented in Tables 1, 2, and 4, respectively. The variation of integrated absorbance for *Tt*-GA and four groups of new lines at each step of the H + *Tt-*GA experiment are summarized in Table S3. It is noteworthy that some lines in groups A′, B′, and D′ were also observed in H + *Cc*-GA experiments, as indicated in Figures 2 and S2, but intensities of these lines were much smaller than those in H + *Tt*-GA experiments.

**Table 1:** Comparison of Observed Vibrational Wavenumbers and Relative IR Intensities of Lines in Groups A and A′ with Scaled Harmonic Vibrational Wavenumbers and IR Intensities of *Cc*-HOCH_2_C^•^O (**3**) and *Tt*-HOCH_2_C^•^O (**3′**) Predicted with the B3LYP/aug-cc-pVTZ Method

mode	sym.	*Cc*-HOCH_2_C^•^O (**3**)				*Tt*-HOCH_2_C^•^O (**3′**)				mode description^c^
		group A/*p*-H_2_		B3LYP/aug-cc-pVTZ		group A′/*p*-H_2_		B3LYP/aug-cc-pVTZ		
		ν /cm^–1^	int.^a^ /%	ν^b^ /cm^–1^	int./km mol^–1^	ν/cm^–1^^a^	int. /%^b^	ν/cm^–1^	int. km mol^–1^	
ν_1_	a′	3626.5	45	3628	41	3638.1	56	3654	50	ν OH
ν_2_	a′^d^			2981	4	e		2908	14	ν_a_ CH_2_
ν_3_	a′	e		2881	22	e		2869	36	ν_s_ CH_2_
ν_4_	a′	1867.9	100	1869	120	1873.3	100	1877	130	ν C=O
ν_5_	a′	1425.0	18	1427	17	1444.5	7	1434	13	δ CH_2_
ν_6_	a′	f		1359	39			1360	3	δ COH/ω CH_2_
ν_7_	a′			1289	3	1185.4	85	1198	96	ω CH_2_/δ COH
ν_8_	a′^d^	1215.6	g	1192	21			1199	0	*t* CH_2_/δ COH
ν_9_	a′	1031.4	96	1034	144	1073.5	93	1076	106	ν CO
		1054.6?								
ν_10_	a′			870	6			823	6	γ CH_2_
ν_11_	a′			781	1			845	8	ν CC
ν_12_	a′	677.9	20	680	21			526	2	δ CCO
ν_13_	a′^d^			296	56			334	4	δ OH (*oop*)
ν_14_	a′^d^			238	6			152	0	*def* (*oop*)
ν_15_	a′^d^			198	72			239	111	τ C–O

a IR intensities as percent of that of the most intense line in each species.

b Harmonic vibrational wavenumbers scaled according to 0.9619*x* + 24.6 for wavenumbers <2500 cm^–1^ and 0.9200*x* + 133.4 for wavenumbers >2500 cm^–1^.

c Approximate mode description; ν: stretch, ν_s_: symmetric stretch, ν_a_: antisymmetric stretch, δ: bend, ω: wag, γ: rock, *t*: twist, τ: torsion, *def*: deformation, *oop*: out-of-plane.

d For *Tt*-HOCH_2_C^•^O (**3′**), the symmetry of this mode is a”.

e Interference due to the absorption of HCl and HCl-H_2_O complexes.

f Interference due to the absorption of parent.

g A shoulder to the intense *Tt*-GA line at 1203.8 cm^–1^ so that intensity cannot be determined accurately.

**Table 2:** Comparison of Observed Vibrational Wavenumbers and Relative IR Intensities of Lines in Groups B and B′ with Scaled Harmonic Vibrational Wavenumbers and IR Intensities of *Cc*-HOC^•^HC(O)H (**4**) and *Tt*-HOC^•^HC(O)H (**4′**) Predicted with the B3LYP/aug-cc-pVTZ Method

mode	sym.	*Cc*-HOC^•^HC(O)H (**4**)				*Tt*-HOC^•^HC(O)H (**4′**)				mode description^c^
		group B/*p*-H_2_		B3LYP/aug-cc-pVTZ		group B′/*p*-H_2_		B3LYP/aug-cc-pVTZ		
		ν/cm^–1^	int.^a^/%	ν^b^/cm^–1^	int./km mol^–1^	ν/cm^–1^^a^	int./%^b^	ν/cm^–1^	int. km mol^–1^	
ν_1_	a′	3371.8	24	3393	59	3629.6		3645	136	ν OH
ν_2_	a′			3100	7			3059	6	ν _HO_CH
ν_3_	a′	d		2903	53	e		2840	66	ν _O=_CH
ν_4_	a′	1549.5	67	1529	92	1554.8	15	1557	34	ν C=O
ν_5_	a′	1493.2	49	1493	67	1487.3	23	1476	58	ν CC/ν CO (*oph*)
ν_6_	a′			1375	5	f		1334	25	ν CO/δ OH/δ _O=_CH (*ip*)
ν_7_	a′	1308.8	24	1311	32	1245.9	100	1231	231	δ OH/δ CH (*ip*)/ν CO^i^
ν_8_	a′	1176.3	100	1185	140	1214.4	31	1222	68	δ OH^j^/δ _HO_CH (*ip*)/ν CO
ν_9_	a′	1002.4	g	1002	66	1074.7	h	1069	33	ν CC/δ OH (*ip*)^k^
ν_10_	a′	760.9	56	796	22			586	2	δ CCO/δ CC=O (*oph*)
ν_11_	a′			285	28			338	14	δ CCO/δ CC=O (*iph*)
ν_12_	a”			925	1			950	1	δ _O=_CH (*oop*)
ν_13_	a”	793.5	83	803	112	734.3	12	716	19	δ OH (*oop*)^k^/δ CH (*oop*)
ν_14_	a”			653	4			419	123	*def* (*oop*)
ν_15_	a”			426	9			257	9	τ C–C

a IR intensities as percent of that of the most intense line in each species.

b Harmonic vibrational wavenumbers scaled according to 0.9619*x* + 24.6 for wavenumbers <2500 cm^–1^ and 0.9200*x* + 133.4 for wavenumbers >2500 cm^–1^.

c Approximate mode description; ν: stretch, δ: bend, *def*: deformation, τ: torsion, *ip*: in-plane, *oop*: out-of-plane, *iph: in-phase, oph: out-of-phase*.

d Interference due to absorption of HCl.

e Interference due to the absorption of parent.

f Interference due to the absorption of C^•^H_2_OH.

g A shoulder to the intense line of *Tt*-GA at 1000.2 cm^–1^.

h A shoulder to the intense line of *Tt*-HOCH_2_C^•^O (**3**) at 1073.5 cm^–1^.

i The mode ν CO is only for *Tt*-HOC^•^HC(O)H.

j The mode δ OH (*ip*) is only for *Cc*-HOC^•^HCC(O)H.

k The mode δ OH (*ip*) is only for *Tt*-HOC^•^HC(O)H.

## Discussion

### Quantum-Chemical Calculations

**Figure 4:**
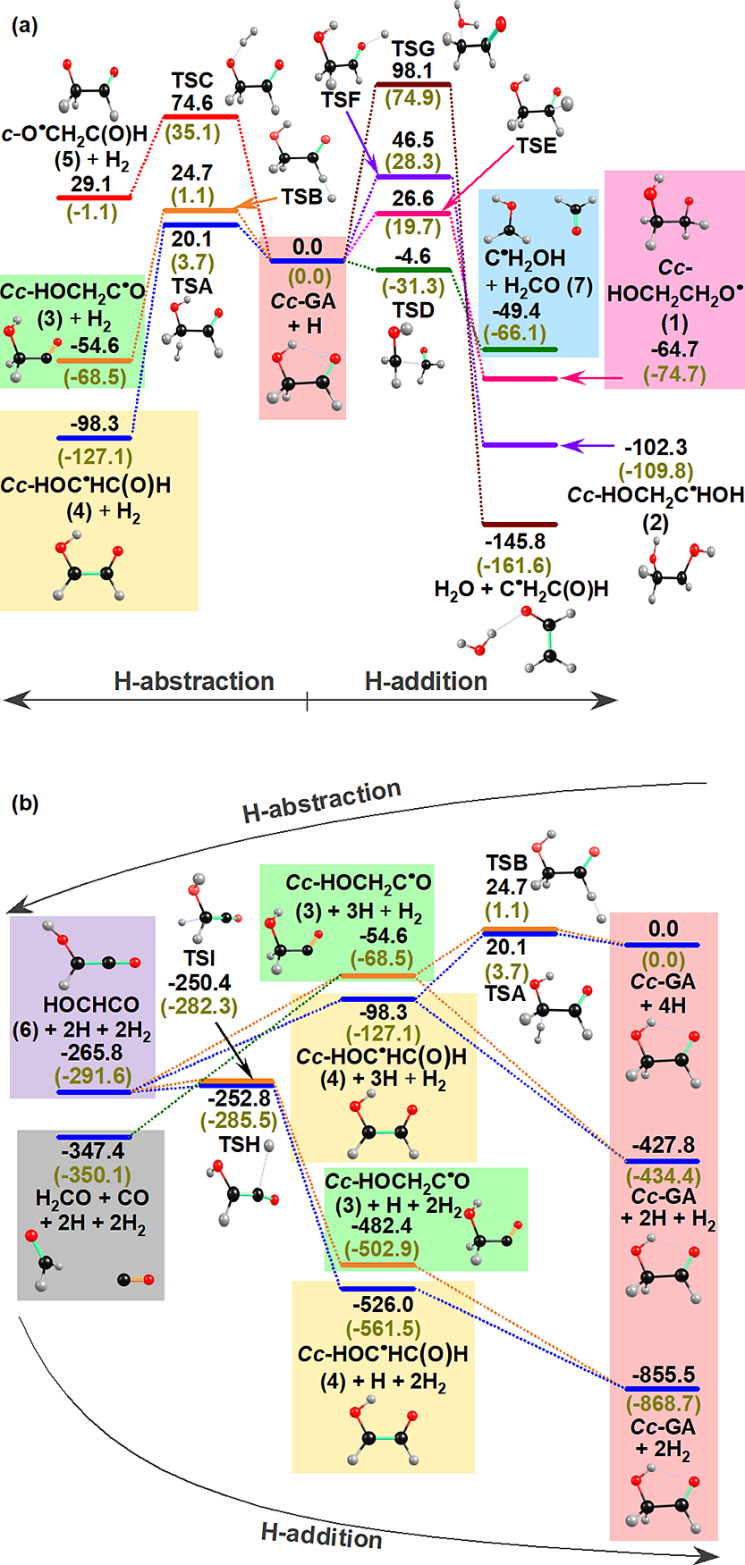
Potential-energy scheme of various channels predicted for reaction H + *Cc*-HOCH_2_C(O)H (*Cc*-GA). (a) All possible H-abstraction and H-addition channels of H + *Cc-*GA; energies are relative to that of H + *Cc-*GA. (b) Two most feasible successive H-abstraction channels followed by two H-addition channels connecting *Cc*-GA with HOCHCO (**6**) via *Cc*-HOC^•^HC(O)H (**4**) or *Cc*-HOCH_2_C^•^O (**3**) and possible formation of fragmented products H_2_CO + CO; energies are relative to 4H + *Cc-*GA. Energies (in kJ mol^–1^) were calculated with the CCSD(T)/aug-cc-pVTZ//B3LYP/aug-cc-pVTZ method (black); values in parentheses (olive) were calculated with the B3LYP/aug-cc-pVTZ method. Zero-point vibrational energies (ZPVE), calculated with the B3LYP/aug-cc-pVTZ method, were corrected.

In order to investigate possible products of H + *Cc*-GA, we performed quantum-chemical calculations with the B3LYP/aug-cc-pVTZ method; geometries of products via H-addition or H-abstraction channels are depicted in Figures S6 and S7, respectively. The potential-energy scheme (PES) for H-abstraction (left side) and H-addition (right side) paths of the reaction H + *Cc*-GA are presented in Figure 4a; listed values (black) were calculated with the CCSD(T)/aug-cc-pVTZ//B3LYP/aug-cc-pVTZ method; those with the B3LYP/aug-cc-pVTZ method are listed in parentheses (olive) for comparison. According to CCSD(T) calculations, the H abstraction from the CH_2_ and C(O)H moieties of *Cc-*GA, resulting in the formation of *Cc*-HOC^•^HC(O)H (**4**) + H_2_ and *Cc*-HOCH_2_C^•^O (**3**) + H_2_, have barriers of 20 and 25 kJ mol^–1^ and exothermicities of 98 and 55 kJ mol^–1^, respectively. In contrast, the H abstraction from the OH moiety, resulting in the formation of *c*-O^•^CH_2_C(O)H (**5**) + H_2_, is unlikely to occur because this reaction is endothermic by 29 kJ mol^–1^ and has a large barrier of 75 kJ mol^–1^. The H addition to the C atom of the C=O moiety of *Cc*-GA results in the formation of *Cc*-HOCH_2_CH_2_O^•^ (**1**) via a small barrier 27 kJ mol^–1^. The H addition to the O atom of the C=O moiety, resulting in the formation *Cc*-HOCH_2_C^•^HOH (**2**), is exothermic by 102 kJ mol^–1^ but with a large barrier of 47 kJ mol^–1^; whereas the H addition to the O atom of the OH moiety leading to the rupture of the C–O bond to form H_2_O + C^•^H_2_C(O)H is the most exothermic (by 146 kJ mol^–1^), but it involves the largest barrier, 98 kJ mol^–1^, and is hence unlikely to occur. The geometries of all transition states involved in these channels are presented in Figure S8. Surprisingly, our predictions revealed that a second channel for the attack of a hydrogen atom on the C atom of the C=O moiety of *Cc*-GA resulted in the formation of C^•^H_2_OH + H_2_CO (**7**) via a barrierless C–C bond rupture, for which a barrier is expected; more sophisticated theoretical investigations are required to gain a deeper understanding of this channel.

The PES depicted in Figure 4b represents consecutive H abstraction of *Cc-*GA and the back H-addition channels. The H abstraction on the C(O)H moiety of *Cc*-HOC^•^HC(O)H (**4**) or on the CH_2_ moiety of *Cc*-HOCH_2_C^•^O (**3**) leads to the formation of the same product, HOCHCO (**6**) + H_2_, through barrierless paths. Alternatively, the barrierless H abstraction from the OH moiety of *Cc*-HOCH_2_C^•^O (**3**) results into fragmentation to products H_2_CO + CO + H_2_ via the C–C bond cleavage. The back H-addition paths H + HOCHCO (**6**) to form (**3**) or (**4**) have small barriers, ∼13 and 15 kJ mol^–1^, respectively. The H-addition paths of (**3**) or (**4**) to form *Cc-*GA are barrierless. A comprehensive discussion on the PES of the reaction H + *Tt*-GA is presented in Supporting Information Note 1 and Figure S9; the geometries of associated transition states are presented in Figure S10.

### Assignments of Products from Hydrogen Abstraction

**Figure 5:**
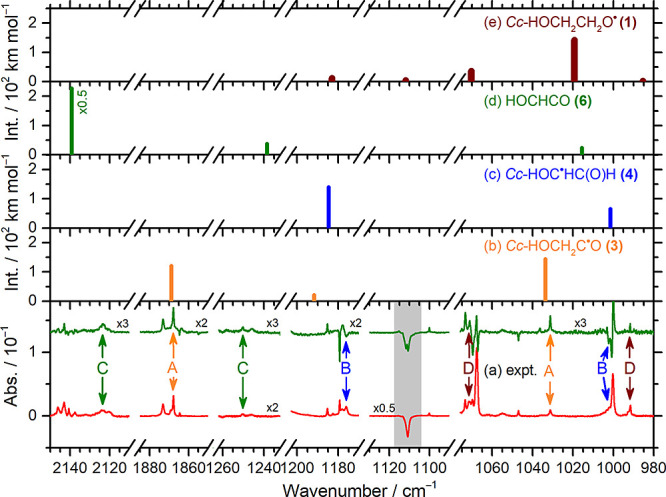
Comparison of lines in groups A–D with predicted IR stick spectra of possible products of H + *Cc-*GA in representative spectral regions. (a) Lower trace is from Figure 3c and the upper trace from Figure 3d; lines in groups A–D are labeled and marked with orange, blue, green, and brown arrows. The predicted stick spectra are (b) *Cc*-HOCH_2_C^•^O (**3**), (c) *Cc*-HOC^•^HC(O)H (**4**), (d) HOCHCO (**6**), and (e) *Cc*-HOCH_2_CH_2_O^•^ (**1**), according to scaled harmonic vibrational wavenumbers. The region severely interfered with by the *Cc*-GA absorption is colored gray.

Lines in group A depicted in the bottom trace of Figures 5a and S11a, taken from Figures 3c and S2c, are compared with the IR stick spectra of (**3**), (**4**), (**6**), and (**1**) according to the vibrational wavenumbers and harmonic IR intensities predicted with the B3LYP/cc-pVTZ method, shown in Figures 5b–e and S11b–e; the harmonic vibrational wavenumbers were scaled as discussed in the Methods section. A detailed spectral assignment is presented in Supporting Information Note 2.

Lines in group A agree satisfactorily with the IR stick spectra of *Cc*-HOCH_2_C^•^O (**3**), Figures 5b and S11b, in terms of vibrational wavenumbers and relative IR intensities. Table 1 compares the observed vibrational wavenumbers and relative IR intensities of lines in group A with the predicted scaled harmonic vibrational wavenumbers and IR intensities of *Cc*-HOCH_2_C^•^O (**3**). The key structural change from GA to (**3**) is the decreased length of the C=O bond and hence the diminished hydrogen bonding due to abstraction of the formyl hydrogen. The OH-stretching (ν_1_) wavenumber of (**3**), observed at 3626.5 cm^–1^, is greater than that of the hydrogen-bonded OH of *Cc-*GA, 3537.8 cm^–1^, supporting the diminished H-bonding in (**3**). The wavenumber of the most intense mode, C=O stretch (ν_4_) observed at 1867.9 cm^–1^, is much greater than the corresponding value at 1746.6 cm^–1^ of *Cc-*GA, supporting a stronger C=O bond in (**3**) after H abstraction of *Cc*-GA; the predicted C=O bond length decreased from 1.207 to 1.179 Å. Most predicted lines of (**3**) with IR intensity >20 km mol^–1^ were observed, as shown in Table 1. We hence assigned lines in group A to *Cc*-HOCH_2_C^•^O (**3**).

Lines in group B depicted in Figures 5a and S11a, agree satisfactorily with the IR stick spectrum of *Cc*-HOC^•^HC(O)H (**4**) shown in Figures 5c and S11c. Table 2 compares the observed vibrational wavenumbers and relative IR intensities of the lines in group B with those predicted for *Cc*-HOC^•^HC(O)H (**4**). The key structural changes from GA to (**4**) are the reduced strength of the C=O bond and the increased strength of the C–C and C–O bonds due to the delocalization over OCCO. The OH-stretching (ν_1_) mode of (**4**), observed at 3371.8 cm^–1^, has a wavenumber much smaller than that of the hydrogen-bonded OH of *Cc-*GA, 3537.8 cm^–1^, supporting an enhanced H-bonding in (**4**). The wavenumber of the intense C=O stretch (ν_4_) mode observed at 1549.5 cm^–1^ is much smaller than the corresponding value of 1746.6 cm^–1^ of *Cc-*GA, supporting a much weaker C=O bond in (**4**); the predicted C=O bond length increased from 1.207 to 1.242 Å. Most predicted lines of (**4**) with IR intensity >20 km mol^–1^ were observed (Table 2). We hence assigned lines in group B to *Cc*-HOC^•^HC(O)H (**4**).

The vibrational wavenumbers and IR intensities of lines in group C, shown in Figures 5a and S11a, agree satisfactorily with the IR stick spectrum predicted for hydroxyketene, HOCHCO (**6**), shown in Figures 5d and S11d. Table 3 compares the observed vibrational wavenumbers and relative IR intensities of the lines in group C with those predicted for HOCHCO (**6**). The main difference in the structure of (**6**) as compared with those of (**3**) and (**4**) is the presence of the C=C bond with a bond length, 1.318 Å, much shorter than those (1.520 and 1.418 Å) of the C–C bond in (**3**) and (**4**), respectively. The most intense line corresponding to the characteristic *anti*-symmetric C=C=O stretch (ν_3_) was observed at 2123.6 cm^–1^; other lines are much weaker. We assigned lines in group C to HOCHCO compound (**6**).

**Table 3:** Comparison of Observed Vibrational Wavenumbers and Relative IR Intensities of Lines in Group C with Scaled Harmonic Vibrational Wavenumbers and IR Intensities of HOCHCO (**6**) Predicted with the B3LYP/aug-cc-pVTZ Method

mode	sym.	HOCHCO (**6**)				mode description^c^
		group C/*p*-H_2_		B3LYP/aug-cc-pVTZ		
		ν/cm^–1^	int.^a^ /%	ν^b^ /cm^–1^	int./km mol^–1^	
ν_1_	a′	3617.0	11	3602	52	ν OH
ν_2_	a′	3033.2	3	3057	17	ν CH
ν_3_	a′	2123.6	100	2139	506	ν_a_ C=C=O
ν_4_	a′			1396	18	ν CC/δ OCH/ δ COH
ν_5_	a′	1250.2	8	1239	38	δ COH /δ OCH
ν_6_	a′	1137.4?^d^	15	1155	74	ν CO
ν_7_	a′			1016	24	ν CO/δ CCH
ν_8_	a′			688	6	δ CC=O/δ CCO (*iph*)
ν_9_	a′			580	46	δ CH (*oop*)
ν_10_	a′			519	26	*def* (*oop*)
ν_11_	a′			237	6	δ CC=O/δ CCO (*oph*)
ν_12_	a′			289	109	τ C–O

a IR intensities as percent of that of the most intense line at 2123.6 cm^–1^.

b Harmonic vibrational wavenumbers scaled according to 0.9619*x* + 24.6 for wavenumbers <2500 cm^–1^ and 0.9200*x* + 133.4 for wavenumbers >2500 cm^–1^.

c Approximate mode description; ν: stretch, ν_a_: antisymmetric stretch, δ: bend, *def*: deformation, τ: torsion, *oop*: out-of-plane, *iph*: in-phase, *oph:* out-of-phase.

d Interference due to absorption of parent.

### Assignments of Products from Hydrogen Addition

A detailed spectral assignment is presented in Supporting Information Note 2. Lines in group D are compared to the predicted IR stick spectra of H-reaction products in Figures 5 and S11. These lines agree satisfactorily with the predicted IR stick spectra of *Cc*-HOCH_2_CH_2_O^•^ (**1),** as presented in Figures 5e and S11e; the experimental results of lines in group D are compared with those predicted for (**1**) in Table 4. The main difference in the structures between (**1**) and *Cc-*GA is that the C=O bond in *Cc-*GA becomes a C–O bond in (**1**), with the bond length increased from 1.207 to 1.364 Å. The vibrational wavenumbers for the coupled C–O_H_ and C–O^•^ stretches (modes ν_14_ and ν_13_) were observed at 991.7 (most intense) and 1071.4 cm^–1^, respectively. The line associated with the _O_CH_2_–rocking (ν_17_) mode was observed at 765.2 cm^–1^. We hence assigned lines in group D to *Cc*-HOCH_2_CH_2_O^•^ (**1**) (Table 4).

**Table 4:** Comparison of Observed Vibrational Wavenumbers and Relative IR Intensities of Lines in Groups D and D′ with Scaled Harmonic Vibrational Wavenumbers and IR Intensities of *Cc*-HOCH_2_CH_2_O^•^ (**1**) and *Tt*-HOCH_2_CH_2_O^•^ (**1′**) Predicted with the B3LYP/aug-cc-pVTZ Method

mode	sym.	*Cc*-HOCH_2_CH_2_O^•^ (**1**)				*Tt*- HOCH_2_CH_2_O^•^ (**1′**)				mode description^c^
		group D/*p*-H_2_		B3LYP/aug-cc-pVTZ		group D′*/p*-H_2_		B3LYP/aug-cc-pVTZ		
		ν/cm^–1^	int.^a^/%	ν^b^ /cm^–1^	int./km mol^–1^	ν/cm^–1^	int./%	ν/cm^–1^	int. km mol^–1^	
ν_1_	a′	3610.5	27	3607	37	3656.3	57	3660	43	ν OH
ν_2_	a′^d^	e		2966	24	e		2926	40	ν_a HO_CH_2_
ν_3_	a′	2905.5?	f	2885	57	f		2892	46	ν_s HO_CH_2_
ν_4_	a′^d^	e		2848	27			2795	1	ν_a O_CH_2_
ν_5_	a′			2767	6	f		2794	16	ν_s O_CH_2_
ν_6_	a′			1472	1			1487	3	δ _HO_CH_2_
ν_7_	a′	e		1384	38			1416	2	ω _HO_CH_2_/δ COH
ν_8_	a′^d^	1354.8	11	1343	14			1254	1	δ COH/*t*_HO_CH_2_
ν_9_	a′	g		1336	15	1375.5	13	1356	16	δ _O_CH_2_/*t* CH_2_^h^
ν_10_	a′	e		1232	32	1293.6	50	1284	34	*t*_HO_CH_2_/δ _O_CH_2_
ν_11_	a′^d^	i		1183	12			1165	2	*t*_O_CH_2_/ν CC
ν_12_	a′			1112	4	1210.9	71	1206	50	*t*_O_CH_2_/δ CCH^h^
ν_13_	a′	1071.4	25	1070	37	1061.6	15	1059	19	ν CO/ν CO_H_ (*iph*)
ν_14_	a′	991.7	100	1019	141	1036.4	100	1039	67	ν CO/νCO_H_ (*oph*)
ν_15_	a′^d^			985	3			887	2	γ _HO_CH_2_/γ _O_CH_2_
ν_16_	a′			851	13	992.6	j	980	18	ν CC
ν_17_	a′	765.2	20	765	23			433	32	γ _O_CH_2_
ν_18_	a′			539	9			493	4	δ CCO/δ CCH
ν_19_	a′^d^			412	109			242	88	τ C–O_H_
ν_20_	a′			306	9			305	18	δ CCO/τ C–C
ν_21_	a′^d^			161	16			134	20	τ C–C

a IR intensities as percent of that of the most intense line in each species.

b Harmonic vibrational wavenumbers scaled according to 0.9619*x* + 24.6 for wavenumbers <2500 cm^–1^ and 0.9200*x* + 133.4 for wavenumbers >2500 cm^–1^.

c Approximate mode description; ν: stretch, ν_s_: symmetric stretch, ν_a_: antisymmetric stretch, δ: bend, ω: wag, γ: rock, *t*: twist, τ: torsion, *iph: in-phase, oph: out-of-phase*.

d For *Tt*-HOCH_2_CH_2_O^•^ (**1′**), the symmetry of this mode is a″.

e Interference due to the absorption of parent.

f Interference due to the absorption of HCl and HCl-H_2_O complexes.

g Interference due to the absorption of C^•^H_2_OH.

h For *Tt*-HOCH_2_CH_2_O^•^ (**1′**), this mode is ω CH_2_/δ COH.

i Interference due to the absorption of *Tt*-HOCH_2_CO^•^ (**3′**).

j A shoulder to the intense line of *Cc*-HOCH_2_CH_2_O^•^ (**1**) at 991.7 cm^–1^.

The destruction of *Cc*-HOCH_2_C^•^O (**3**) at 460 and 520 nm and *Cc*-HOCH_2_CH_2_O^•^ (**1**) at 460 nm during secondary photolysis agrees well with the UV spectra predicted with the TD-B3LYP/aug-cc-pVTZ method. A description on their photodecomposition is provided in Supporting Information Note 3 (Figures S12–S14). We also compared the observed lines with the predicted IR stick spectra of other possible products, *c*-O^•^CH_2_C(O)H (**5**), H_2_O + C^•^H_2_C(O)H, and *Cc*-HOCH_2_C^•^HOH (**2**), as presented in Figure S15; lines in group A–D agree poorly with those predicted for these species.

In addition to lines in groups A–D, we also observed lines of C^**•**^H_2_OH at 3651.9, 3165.3, 3038.5, 1457.8, 1332.1, 1179.3, and 1046.9 cm^–1^,^53^ lines of H_2_CO at 2782.9 and 1742.6 cm^–1^,^52^ and rovibrational lines of CO around 2143 cm^–1^.^57^ The formation of C^**•**^H_2_OH and H_2_CO is in line with the PES depicted in Figure 5 in which the H-attack to the C atom of the C=O moiety of *Cc-*GA resulted in the rupture of the C–C bond and subsequent formation of C^**•**^H_2_OH and H_2_CO, reaction 7, which was not included in the calculations by Álvarez-Barcia et al.^31^ Furthermore, the H abstraction on the OH moiety of *Cc*-HOCH_2_C^**•**^O (**3**) results in fragmented products H_2_CO + CO; this could be one of the pathways for the formation of H_2_CO and CO.

After prolonged irradiation of *Cc-*GA at 3537 cm^**–1**^ to convert it to *Tt*-GA, the reaction of H atoms with *Tt*-GA produced lines in groups A′, B′, C, and D′. The variations of integrated absorbance for these groups of new features, C^**•**^H_2_OH, H_2_CO, HC^**•**^O, and CO at each step of the H + *Tt*-GA experiment are summarized in Table S3. Following the similar consideration, we assigned lines in groups A′, B′, and C to H-abstraction products *Tt*-HOCH_2_C^**•**^O (**3′**), *Tt*-HOC^**•**^HC(O)H (**4′**), and HOCHCO (**6**), respectively, and lines in group D′ to the H-addition product *Tt*-HOCH_2_CH_2_O^**•**^(**1′**), as presented in Figures S16 and S17 and discussed in Supporting Information Note 4.

### Temporal Profiles and Reaction Mechanism

**Figure 6:**
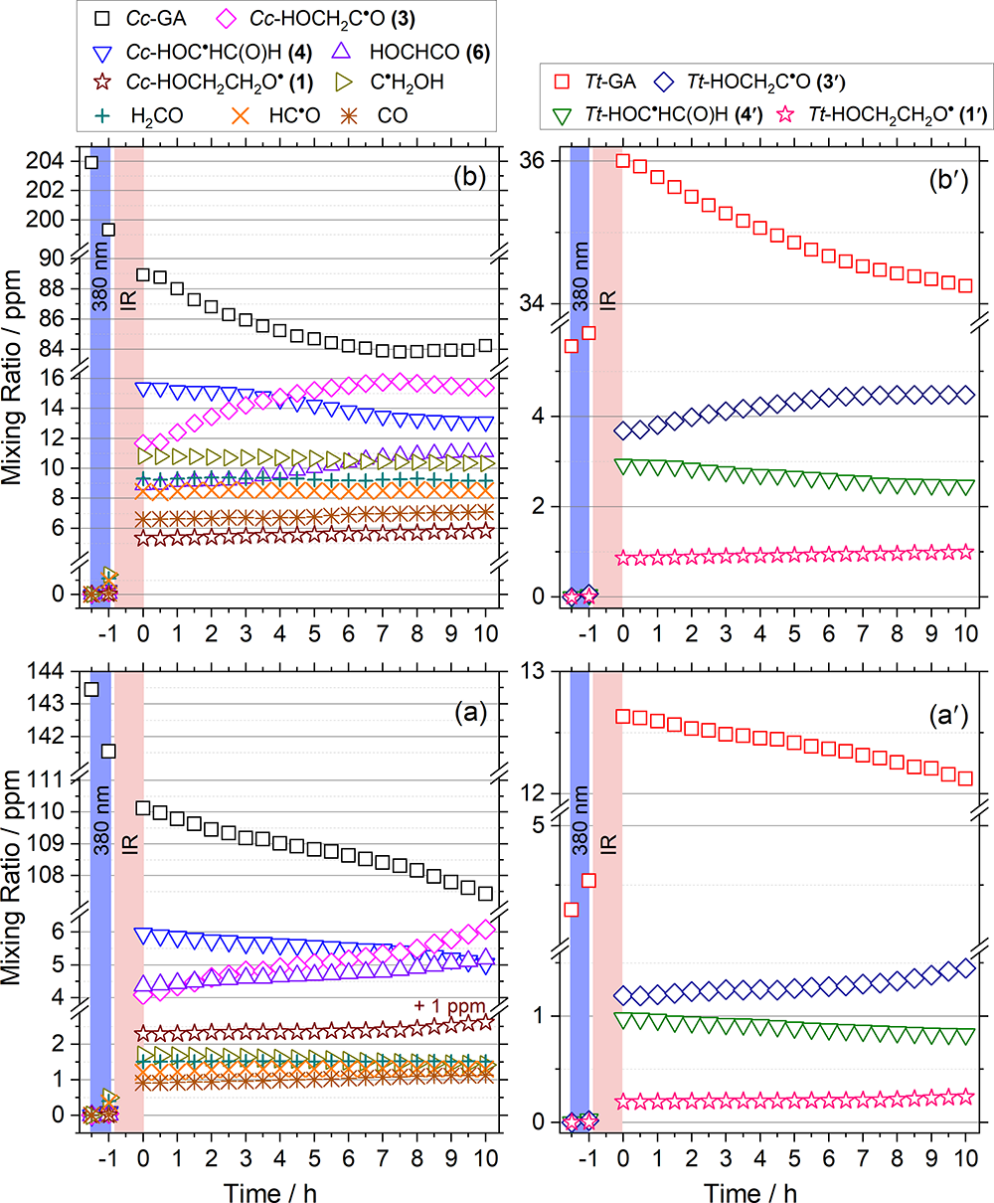
Temporal evolution of mixing ratios of *Cc*-GA and products formed in the reaction H + *Cc*-GA. Left panels represent temporal profiles of products from the reaction H + *Cc*-GA, whereas right panels represent temporal profiles of products from the reaction H + *Tt*-GA; *Tt*-GA was produced mainly after IR irradiation. (a) H-deficient experiment for the reaction H + *Cc*-GA: [H]_0_/[*Cc*-GA] ≈ 2.3 and [*Cc*-GA]_0_ = 143.4 ppm. (a′) H-deficient experiment for species related to H + *Tt*-GA. (b) H-rich experiment for the reaction H + *Cc*-GA: [H]_0_/[*Cc*-GA] ≈ 7.5 and [*Cc*-GA]_0_ = 203.9 ppm. (b′) H-rich experiment for species related to H + *Tt*-GA. [H]_0_ was estimated from [HCl]_0_. The profile of *Cc*-HOCH_2_CH_2_O^•^ (**1**) in (a) was shifted up by 1 ppm for clarity. The regions shaded with blue and red correspond to the period of 380 nm and IR irradiation, respectively.

The estimated mixing ratios of observed species in each step of two experiments of *Cc-*GA, with [H]_0_/[*Cc-*GA]_0_ ≈ 2.3 ([*Cc*-GA]_0_ = 143 ± 6 ppm) and 7.5 ([*Cc*-GA]_0_ = 204 ± 8 ppm), are listed in Table S4; a detailed description on the estimation of mixing ratios is discussed in Supporting Information Note 5. Due to uncertainties in the calculated IR intensities of each species, the absolute values in mixing ratios might have uncertainties as large as a factor of 2, but the percentage changes in mixing ratios of a specific species in various stages of experiments are considered to be reliable. To alleviate potential errors associated with predicted IR intensities, we averaged values derived from several spectral lines whenever possible. The integrated regions of each species used for the estimation of mixing ratios are listed in Table S8. The standard deviations in fitting various lines of each species are listed in the caption of Table S4. Behaviors in two periods allowed for the reaction H + *Cc-*GA, the first during IR irradiation (time = −1.0 to 0 h) and the second in darkness (time = 0.0–10.0 h), are quite distinct, as shown in Figure 6 (expanded version for small mixing ratios in Figure S18). Although the estimated mixing ratios might have large uncertainties, we note that the sum of mixing ratios of all products (including those associated with *Tt-*GA) after IR irradiation was 31 and 110 ppm for the H-deficient and the H-rich experiments, respectively. These values agree well with the loss of *Cc-*GA (including conversion of *Cc-*GA to *Tt-*GA) at 31 and 110 ppm in two experiments, respectively. However, in darkness, the sum of mixing ratios of all products of *Cc-*GA, 2.2 and 4.1 ppm, is slightly smaller than the loss of *Cc-*GA, 2.6 and 4.7 ppm, respectively, likely because of the matrix evaporation over a prolonged period (thickness of the matrix decreased slightly from 1.1 to 0.9 mm in darkness); the mixing ratios of *Tt*-GA and its products were not considered in this case because of their small values, hence large uncertainties. Furthermore, the fractions of conversion from *Cc-*GA to *Tt*-GA upon IR irradiation in both H-deficient and H-rich experiments, 26 and 27%, respectively, are consistent.

In darkness, in the H-deficient experiment, the mixing ratios of *Cc-*GA, *Tt*-GA, *Cc*-HOC^•^HC(O)H (**4),** and *Tt*-HOC^•^HC(O)H (**4′**) decreased continuously, whereas those of *Cc*-HOCH_2_C^•^O (**3**), *Tt*-HOCH_2_C^•^O (**3′**), HOCHCO (**6**), *Cc*-HOCH_2_CH_2_O^•^ (**1**), and *Tt*-HOCH_2_CH_2_O^•^ (**1′**) increased continuously. The *anti*-correlations between *Cc-*GA and (**3),** between (**4**) and (**6**), and between *Tt-*GA and (**3′**) are clearly demonstrated. For the H-rich experiment, in darkness, these *anti*-correlations are also obvious; the mixing ratio of *Cc-*GA continuously decreased to reach a minimum after ∼8 h and then increased slightly afterward, whereas that of (**3**) increased continuously, reached to a maximum after ∼8 h, then decreased slightly. This is an indication that the H addition to (**3**) to form back *Cc-*GA became more important at a later stage in the H-rich experiment. The ratios of the increase of (**3**) and (**1**) relative to the decay of *Cc-*GA remain the same, indicating that the formation of (**3**) and (**1**) is from the same reaction, namely H + *Cc-*GA.

In darkness, (**3**) and (**6**) increased with time, whereas (**4**) decreased with time, indicating that the reaction of H with (**4**) to form (**6**) is more facile than that of H with *Cc-*GA to form (**4**), in agreement with the PES showing that the former reaction is barrierless whereas the latter has a barrier ∼20 kJ mol^–1^. The ratios of the increase of (**6**) relative to the decrease of *Cc-*GA in the H-deficient experiment are ∼70% of those in the H-rich experiment, supporting the fact that in the H-rich experiments, the second H-abstraction path became more important. In the H-rich experiment, both (**4**) and (**6**) appeared to be approaching to a constant value, indicating that the H-addition reaction H + (**6**) to form back (**4**) became more important at the later stage.

The variations of the mixing ratios of C^•^H_2_OH, H_2_CO, HC^•^O, and CO in darkness were small (<0.3 ppm in the H-deficient experiment and <0.5 ppm in the H-rich experiment); the mixing ratios of H_2_CO and HC^•^O were nearly constant, whereas that of C^•^H_2_OH decreased over time and that of CO increased by a similar amount. These fragmented products might react with H atoms, which are readily available. PES of reactions of H + C^•^H_2_OH and H_2_CO involving H-abstraction and H-addition pathways is depicted in Figure S19. The H abstractions from C^•^H_2_OH and HC^•^O are barrierless, whereas that from H_2_CO has a barrier of ∼22 kJ mol^–1^. The H addition paths to C^•^H_2_OH and HC^•^O to form CH_3_OH and H_2_CO, respectively, are barrierless, whereas those to CO and H_2_CO have barriers of ∼11 and 41 kJ mol^–1^, respectively. According to the PES in Figure S19, the formation of CH_3_OH from C^•^H_2_OH is likely, but the most intense absorption lines at 3679.7 and 1031.0 cm^–1^ of CH_3_OH were interfered with by IR absorptions of *Cc*-GA and *Cc*-HOCH_2_C^•^O (**3**), preventing its definitive assignment. Although, in darkness, the intensities of lines of C^•^H_2_OH decreased slightly and those of CO lines increased slightly, indicating that reactions of H atoms with C^•^H_2_OH, H_2_CO, and HC^•^O following successive H-abstraction pathways might eventually form CO, we cannot ensure this mechanism because of the small mixing ratios involved. The detailed mechanism deserves careful examination in separate experiments. Considering that no significant increase of H_2_CO and that the total mixing ratios of C^•^H_2_OH, H_2_CO, HC^•^O, and CO remained about the same in darkness, the contribution of H abstraction of HOCH_2_C^•^O (**3**) to form H_2_CO + CO and H-induced fragmentation of H + *Cc-*GA to form C^•^H_2_OH + H_2_CO (**7**) might be insignificant in darkness.

During the IR irradiation, the variations of the mixing ratios of these products are quite different from those in darkness, presumably because the H atoms produced during IR irradiation had more energy than those in the tunneling reactions in darkness and because the nearest-neighbor reaction played a more important role during IR irradiation. The exothermicity of the reaction Cl + H_2_ (*v* = 1) → HCl + H is ∼45 kJ mol^–1^;^58^ one would expect that H reactions with a larger barrier or even with slight endothermicity might occur during IR irradiation, but not in darkness. In the H-deficient experiment upon IR irradiation, the ratios of production of (**4**), (**3**), (**6**), and (**1),** and the summation of C^•^H_2_OH, H_2_CO, HC^•^O, and CO relative to the loss of *Cc-*GA due to H reactions (excluding the conversion to *Tt*-GA) are approximately 0.28, 0.20, 0.21, 0.11, and 0.20, respectively, whereas that for *Tt*-GA relative to the total loss of *Cc-*GA is 0.26. In the H-rich experiment upon IR irradiation, the ratios of production of (**4**), (**3**), (**6**), and (**1),** and the summation of C^•^H_2_OH, H_2_CO, HC^•^O, and CO relative to the loss of *Cc-*GA due to H reactions (excluding the conversion to *Tt*-GA) are approximately 0.21, 0.16, 0.12, 0.07, and 0.43, respectively, whereas that for *Tt*-GA relative to the total loss of *Cc-*GA is 0.27. The ratios for the conversion from *Cc-*GA to *Tt*-GA are similar in both H-deficient and H-rich experiments, supporting that *Tt*-GA was produced from *Cc-*GA via IR irradiation and that the hydrogen reactions with *Cc-*GA did not produce *Tt*-GA significantly. In contrast, the enhancement of C^•^H_2_OH, H_2_CO, HC^•^O, and CO in the H-rich experiment indicates that the H-induced fragmentation of (**3**) might play a significant role in H-rich experiments. In darkness, in the H-deficient experiment, the ratios of the destruction of (**4**) and the production of (**3**), (**6**), and (**1**) relative to the loss of *Cc-*GA due to H reactions are approximately–0.33, 0.74, 0.30, and 0.15, respectively; whereas, in the H-rich experiment, the ratios became–0.49, 0.79, 0.47, and 0.11, respectively. The significantly larger fraction of the production of (**4**) than (**3**) during IR irradiation as compared with the decay of (**4**) in darkness might imply that reaction 4 that produced (**4**) has a larger barrier than reaction 3 that produced (**3**), which is in contrast to the barriers predicted with the CCSD(T)/aug-cc-pVTZ method, but consistent with those predicted with the B3LYP/aug-cc-pVTZ method in this work and with the MPWB1K/def2-TZVP method reported by Álvarez-Barcia et al.^31^

**Figure 7:**
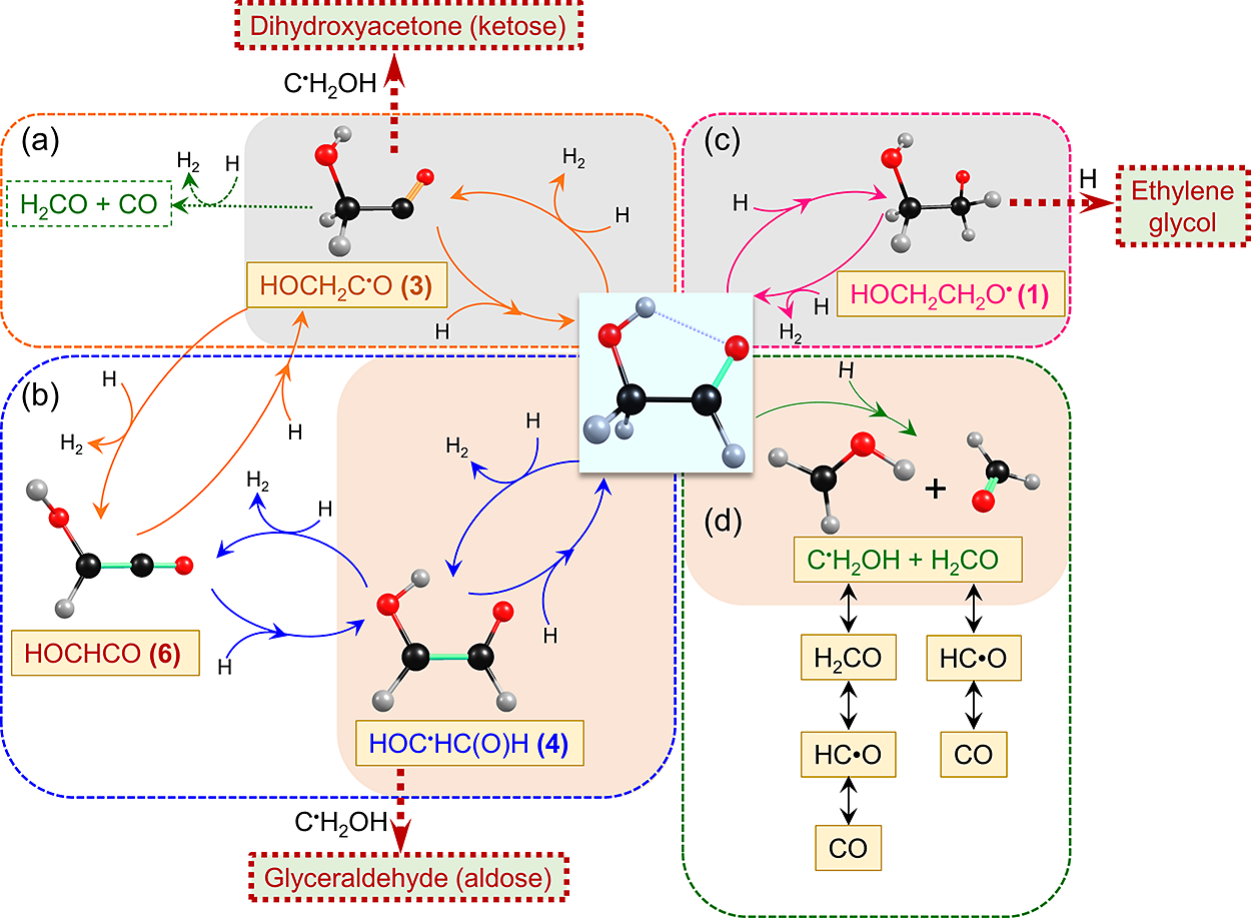
Proposed reaction mechanism in reaction H + *Cc*-GA. (a) H-abstraction/H-addition cycle connecting GA and HOCH_2_C^•^O (**3**) and a likely minor channel involving the formation of H_2_CO + CO. (b) H-abstraction/H-addition cycle connecting GA and HOC^•^HC(O)H (**4**), which is further connected with HOCHCO (**6**) via a second cycle; (**6**) might also be connected with (**3**) via a similar cycle. (c) H-addition/H-abstraction cycle connecting GA and HOCH_2_CH_2_O^•^ (**1**). (d) H-induced fragmentation channel involving the formation of C^•^H_2_OH + H_2_CO (**7**) and successive reactions to form HC^•^O and CO. The light gray background indicates the species that increased in darkness, and the light peach background indicates the species that was enhanced significantly during IR irradiation. The dotted thick brown arrows indicate possible routes for the formation of more complex sugars from the observed radical species.

A summary of the reaction mechanism according to our study is illustrated in Figure 7. In darkness, the tunneling reactions of H atoms with GA led to the formation of HOCH_2_C^•^O (**3**) via H abstraction on the C(O)H moiety of *Cc-*GA (major) and the formation of HOCH_2_CH_2_O^•^ (**1**) via H addition on *Cc-*GA (minor), as indicated with a light gray background in Figure 7; the H addition to (**3**) and H abstraction of (**1**) reproduced *Cc-*GA. The formation of HOCHCO (**6**) via H abstraction from the C(O)H moiety of HOC^•^HC(O)H (**4**), which was produced mainly during IR irradiation, was also observed, as indicated with a light gray background in Figure 7; the possibility that HOCHCO (**6**) was also produced from H abstraction of HOCH_2_C^•^O (**3**) cannot be excluded, even though the temporal profiles indicate that (**4**) and (**6**) are *anti*-correlated. A small fraction of radical C^•^H_2_OH was converted to CO in darkness. The further addition of H to (**1**) may produce EG (HOCH_2_CH_2_OH) via a barrierless path, but we did not observe this product likely because of insufficient H atoms in our experiments. In contrast, during IR irradiation, in which H atoms have more kinetic energy to react with *Cc-*GA, the formation of HOC^•^HC(O)H (**4**) via H abstraction on the CH_2_ moiety of *Cc-*GA and the formation of C^•^H_2_OH + H_2_CO (**7**) via H-induced fragmentation upon H attack on the C atom of the formyl moiety of *Cc-*GA becomes more important, as indicated with a light peach background in Figure 7.

The temporal evolution of all species in the H + *Tt*-GA experiments is presented in Figures S20 and S21 and discussed in Supporting Information Note 6. The behavior is similar to that of H + *Cc-*GA except that the yields are smaller. That the matrix suffered harsh conditions from prolonged IR irradiation during conformational (*Cc* → *Tt*) conversion might cause this difference. The thickness of the matrix decreased from 1.2 to 0.8 mm in darkness which is clearly visible in Figure S5d in which the intensity of the *p*-H_2_ feature near 706 cm^–1^ decreased in darkness. Our experiments indicated that the conformation was retained during the reaction of H + GA.

## Astronomical Implications

GA comprises hydrogen atoms of three types: the formyl HC(O), the methylene CH_2_, and the hydroxy OH. Our experiments demonstrated the richness in the reactions of GA with hydrogen atoms; the reactions resulted in the formation of HOC^•^HC(O)H (**4**) and HOCH_2_C^•^O (**3**) through H abstraction, HOCH_2_CH_2_O^•^ (**1**) via H addition, and C^•^H_2_OH + H_2_CO (**7**) from the H-induced fragmentation. Further, H abstraction of (**4**) or (**3**) produced HOCHCO (**6**). All these three radicals (**1**), (**3**), and (**4**) and the stable species (**6**) are identified with IR absorption for the first time; they are expected to play important roles in the formation of COM. The H-induced fragmentation to form C^•^H_2_OH + H_2_CO (**7**), reaction 7, was overlooked in previous theoretical investigations,^31^ but this type of H-induced fragmentation is not uncommon, as we also observed the formation of HCNO + CH_4_ and CH_2_NH + CO from the H-induced fragmentation of the radicals C^•^(O)NH(CH_3_) and HC(O)NH(C^•^H_2_), produced in the H-abstraction reactions of *N*-methyl formamide, HC(O)NH(CH_3_), in solid *p*-H_2_.^43^ The current astronomical models include mostly H-addition pathways, wherein H abstraction and H-induced fragmentation pathways are rarely considered;^59−61^ incorporation of these H abstraction and H-induced fragmentation channels is crucial for the improvement of the model for the formation of COM. Nevertheless, Watanabe and Kouchi demonstrated possibilities of H abstraction pathways while studying H and D addition reactions on ice surfaces at low temperatures.^62^

Leroux et al. bombarded GA ice at 10 K with H atoms and observed the formation of ethylene glycol (HOCH_2_CH_2_OH, EG);^33^ these authors proposed that the H-addition product (**1**) was the intermediate and further H addition produced EG. Consecutive H addition to GA via the intermediate HOCH_2_C^•^HOH (**2**) to form EG was proposed by Fedoseev et al.^34^ However, Álvarez-Barcia et al.^31^ reported that the barrier ∼40 kJ mol^–1^ for the formation of (**2**), reaction 2, was much higher than that, ∼21 kJ mol^–1^, for the formation of (**1**). As shown in the PES in Figure 4, the formation of (**2**) via reaction 2 has a barrier twice those for the formation of HOCH_2_CH_2_O^•^ (**1**) via H addition and of HOC^•^HC(O)H (**4**) and HOCH_2_C^•^O (**3**) via H abstraction, so that only (**1**), (**3**), and (**4**) were observed in our experiments of H reactions with GA. The barriers and rate coefficients for all possible H-abstraction and H-addition channels of H + GA (reactions 1**–**5) calculated by Álvarez-Barcia et al. also indicated that the formation of (**1**), (**3),** and (**4**) is at least 100 times faster than that of (**2**) in the H + GA reaction.^31^ Our observation of (**1**) supports this mechanism for the formation of (**1**) rather than (**2**). In the interstellar media, further hydrogenation of (**1**) may take place to produce EG, as shown in reaction 8.

Fedoseev et al.^34^ reported the formation of glycerol HOCH_2_CH(OH)CH_2_OH and, tentatively, glyceraldehyde HOCH_2_CH(OH)CHO, when GA and CO molecules were codeposited with H atoms at 15 K. These authors proposed a reaction scheme via the H-added radical intermediate HOCH_2_C^•^HOH (**2**), followed by reactions of (**2**) with HC^•^O and C^•^H_2_OH to form glyceraldehyde and glycerol, respectively,


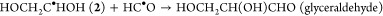
11


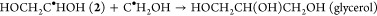
12

Alternatively, glycerol can be produced from glyceraldehyde by successive H addition. From our experiments, it is likely that the proposed mechanism for the formation of glyceraldehyde might not go through the intermediate HOCH_2_C^•^HOH (**2**); the H abstraction of GA might produce HOC^•^HC(O)H (**4**), which reacts further with C^•^H_2_OH to form glyceraldehyde,


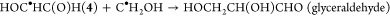
13

in which C^•^H_2_OH was also observed in the H + GA reaction in this work, or in other experiments via successive hydrogenation of CO^63^ or H abstraction on the CH_3_ moiety of CH_3_OH.^36,64^

During IR irradiation, we observed the enhanced formation of C^•^H_2_OH and H_2_CO; the fraction of formation of C^•^H_2_OH and H_2_CO was increased in the H-rich experiment upon IR irradiation. The reaction of H with GA hence serves as a source of C^•^H_2_OH, which is an important intermediate in astrochemistry and was proposed in many reactions.^32,34,45^ For example, the major radical product HOCH_2_C^•^O (**3**) produced from H abstraction of GA in darkness might further react with C^•^H_2_OH to produce ketose,
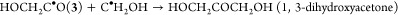
14

The radicals observed in this work could potentially pave the way for the synthesis of higher-order sugar molecules in interstellar environments. Furthermore, the self-reaction of C^•^H_2_OH might form EG on dust grains, as reported in the literature,^64−66^
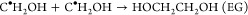
15

This previously neglected channel of H + *Cc-*GA to form C^•^H_2_OH, which depends on the mixing ratio of H, and their subsequent reactions might be one of the reasons why the abundance ratios of [EG]/[GA] changes in various star-forming regions.^13,67^

## Conclusions

In the investigation of the reactions H + *Cc*-/*Tt*-GA in solid *p*-H_2_ at 3.2 K, we identified *Cc*- and *Tt*-conformers of HOC^•^HC(O)H (**4**/**4′**) and HOCH_2_C^•^O (**3**/**3′**) produced via H-abstraction paths, HOCH_2_CH_2_O^•^ (**1**) via the H-addition path, and C^•^H_2_CO + H_2_CO (**7**) via the H-induced fragmentation; further H abstraction of (**4**) or (**3**) produced HOCHCO (**6**). The observation of such rich chemistry in reactions of hydrogen with GA, with four channels of three distinct types, is extraordinary. IR spectra of (**1**), (**3**/**3′**), (**4**/**4′**), and (**6**) investigated herein are previously unreported and these observations are consistent with various feasible paths according to the PES of H + *Cc*-/*Tt*-GA predicted quantum-chemically.

The H abstraction on the C(O)H moiety of *Cc*/*Tt*-GA, resulting in the formation of HOCH_2_C^•^O (**3**/**3′**), is the major path in darkness; its temporal behavior indicates that further H addition to (**3**) reproduced GA. With IR irradiation, the H abstraction of *Cc*/*Tt*-GA on the CH_2_ moiety becomes more important; this reaction leads to the formation of HOC^•^HC(O)H (**4**/**4′**), and the second H abstraction on the formyl moiety of (**4**) forms HOCHCO (**6**). A dual cycle, each consisting of a H-abstraction and a H-addition channel, might chemically connect GA, HOC^•^HC(O)H (**4**/**4′**), and HOCHCO (**6**). The possibility that a similar H-abstraction/H-addition cycle exists between (**3**) and (**6**) cannot be excluded. The H-induced fragmentation channel to form C^•^H_2_CO + H_2_CO (**7**) also becomes more prominent during IR irradiation and when the mixing ratio of H atoms is large. This is the first example in which the branching among possible channels varies significantly during IR irradiation as compared with when the matrix was maintained in darkness.

The formation of HOCH_2_C^•^O (**3**) and HOC^•^HC(O)H (**4**) might react with other radicals such as C^•^H_2_CO to lead to the synthesis of more complex sugars such as dihydroxyacetone (ketose sugar) and glyceraldehyde (aldose sugar), respectively. The production of C^•^H_2_CO from H + GA also plays an important role in radical reactions in astrochemistry. These channels should be included in the astrochemical model for the formation of COM. The radical intermediate HOCH_2_C^•^HOH (**2**), proposed by Fedoseev et al.^34^ to be produced from the H addition of GA to react with HC^•^O and C^•^H_2_OH to form glyceraldehyde and glycerol, respectively, likely cannot compete with the formation of HOCH_2_CH_2_O^•^ (**1**) according to our experimental and theoretical results.

## Methods

The experimental apparatus of an IR-absorption/matrix-isolation system using solid *p*-H_2_ as a matrix host is described in detail elsewhere.^68−70^ A nickel-coated copper plate, maintained at a temperature of ∼3.2 K using a helium compressor, serves as both a cold matrix substrate and a reflective mirror for reflection-type absorption measurements. IR spectra were measured with a Fourier-transform infrared (FTIR) spectrometer (Bruker, VERTEX 80 V), with a KBr beam splitter and a Hg–Cd–Te detector cooled to 77 K. Typically, 400 interferometric scans at spectral resolution 0.25 cm^–1^ were recorded at each stage of experiments. To avoid the undesired formation of H atoms via reactions of excited H_2_ with Cl atoms during data acquisition, a filter (Spectrogon LP-2500, cutoff wavelength 2.4 μm) was placed to block the light with wavenumber >4000 cm^–1^ from the source of the IR spectrometer. The vapor of HOCH_2_C(O)H, GA, was obtained by warming the GA dimer (2, 5-dihydroxy-1,4-dioxane, Combi-Blocks, purity >98%) near 35 °C prior to mixing with *p*-H_2_. A separate line was used for the codeposition of a gaseous mixture of Cl_2_ in *p*-H_2_. A gaseous mixture of GA/Cl_2_/*p*-H_2_ (1/10/10,000) was introduced over a period of 6 h at a total flow rate of ∼7 STP cm^3^ min^–1^ (STP stands for the standard temperature 273 K and pressure 760 Torr). UV irradiation at 380 ± 6 nm from a light-emitting diode (LED) (3 W, Nikkiso Giken) induced the photodissociation of Cl_2_ to generate Cl atoms. Following this, the matrix was subjected to IR irradiation from an external light source of unfiltered heated silicon carbide (SiC) to facilitate the reaction between Cl and vibrationally excited H_2_, resulting in the formation of H and HCl. For secondary photolysis at wavelengths of 520 and 460 nm, a tunable OPO laser (EKSPLA, NT340, repetition rate 10 Hz, pulse duration 5 ns, ∼1.5 mJ) was employed. For the conformational conversion (*Cc* to *Tt*) of GA, the matrix was irradiated with light at 2827 nm (3537 cm^–1^, ν_OH_ absorption of *Cc*-GA) for 15 h using another tunable OPO laser (EKSPLA, NT342, repetition rate 10 Hz, pulse duration 5 ns, ∼1.5 mJ). The mixing ratio of the species of interest was estimated using the method described by Tam and Fajardo^71^ by considering the predicted IR intensities and the determined optical path length,^72^ as discussed in Supporting Information Note 5. To generate *p*-H_2_, normal H_2_ (99.9999%) was passed through a trap at 77 K prior to entering a converter consisting of an iron(III) oxide catalyst maintained at 12.9 K using a separate closed-cycle helium refrigerator.

All computations, including geometry optimizations and vibrational analyses (wavenumbers and IR intensities), were performed with the software package Gaussian 16.^73^ Calculations were employed with the density-functional theory, utilizing B3LYP functionals^74^ and the standard Dunning’s correlation-consistent basis set augmented with diffuse functions, aug-cc-pVTZ.^75^ Single-point electronic energies were computed using the coupled cluster method with single, double, and perturbative triple excitations, CCSD(T),^76^ on geometries derived from the B3LYP/aug-cc-pVTZ method. The zero-point vibrational energies (ZPVE) were corrected based on the harmonic vibrational wavenumbers calculated with the B3LYP/aug-cc-pVTZ method. For scaling the calculated harmonic vibrational wavenumbers, two linear equations *y* = (0.9619 ± 0.0070)*x* + (24.6 ± 9.1) and *y* = (0.9199 ± 0.0257)*x* + (133.4 ± 81.2) were derived for regions below 2500 and above 2500 cm^–1^, respectively, by comparison of experimental values with predicted harmonic vibrational wavenumbers of *Cc*-GA; *y* is the observed wavenumber and *x* is the calculated harmonic vibrational wavenumber. The average absolute deviation between experiments and scaled harmonic vibrational wavenumbers of *Cc*-GA is 5.9 ± 5.5 cm^–1^, which is smaller than the corresponding value of 16.3 ± 14.5 cm^–1^ for anharmonic vibrational wavenumbers. The time-dependent density-functional theory (TD-DFT) using the B3LYP/6-311++G(d,p) method was used to perform the calculations on electronic excitation and oscillator strength.

## Supporting Information Available

PES of the reaction H + *Tt*-GA; assignments of lines in groups A, B, C, and D; photolysis of (**3**) and (**1**) at 460 and 520 nm; assignments of lines in groups A′, B′, and D′; estimates of mixing ratios; temporal profiles of the reaction H + *Tt*-GA; comparison of observed and calculated vibrational wavenumbers and IR intensities of *Cc*-GA and *Tt*-GA; variations of integrated absorbance for *Cc*-GA, *Tt*-GA, and reaction products at each step of the H + *Cc*-GA and H + *Tt*-GA experiments; estimated mixing ratios of observed species in each step of two experiments of *Cc-*GA and *Tt*-GA; vertical excitation wavelengths and oscillator strengths of electronic excitations of (**3**), (**3′**), (**1**), and (**1′**); integrated regions of spectral lines and IR intensities employed in the estimations of mixing ratios; geometries of conformers of GA; full-range spectra of a GA/Cl_2_/*p*-H_2_ matrix recorded at various stages of the H + *Cc*-GA and H + *Tt*-GA experiment; spectra of a GA/Cl_2_/*p*-H_2_ matrix after irradiations at 266 and 2827 nm; representative spectra recorded at various stages of the H + *Tt*-GA experiment; geometries of H-addition, H-abstraction, and H-induced fragmentation products, and transition states in reactions H + *Cc*-GA and H + *Tt*-GA; PES of reactions H + *Tt*-GA and H + C^•^H_2_OH/H_2_CO/HC^•^O/CO; comparison of lines in groups A–D, A′, B′ and D′ with predicted IR stick spectra of possible products; vertical excitation UV spectra of various products formed in the reactions of H + *Cc-*GA and H + *Tt*-GA; frontier MO diagrams of (**3**), (**3′**), (**1**), and (**1′**); temporal evolution of mixing ratios of products formed in the reaction H + *Cc*-GA in darkness and H + *Tt*-GA in darkness (PDF)
