# Supplementary material for: Identification of HOC•HC(O)H, HOCH2C•O, and HOCH2CH2O• Intermediates in the Reaction of H + Glycolaldehyde in Solid Para-Hydrogen and Its Implication to the Interstellar Formation of Complex Sugars
Source: J Am Chem Soc. 2024 Aug 9;146(33):23306–20. doi: 10.1021/jacs.4c05896 (PMC11345754; doi:10.1021/jacs.4c05896)
Supplement: Supplementary file 1 — ja4c05896_si_001.pdf [file ja4c05896_si_001.pdf]

Supporting Information

Identification of HOC•HC(O)H, HOCH$_2$C•O, and HOCH$_2$CH$_2$O• Intermediates in the Reaction of H + Glycolaldehyde in Solid *Para*-Hydrogen and its Implication to the Interstellar Formation of Complex Sugars

Prasad Ramesh Joshi*[a] and Yuan-Pern Lee*[ab]

[a] Department of Applied Chemistry and Institute of Molecular Science, National Yang Ming Chiao Tung University, Hsinchu 300093, Taiwan

[c] Center for Emergent Functional Matter Science, National Yang Ming Chiao Tung University, Hsinchu 300093, Taiwan

* Corresponding authors: prasad.nctu@gmail.com (PRJ); yplee@nycu.edu.tw (YPL)

Table of Contents

Note 1. PES of the reaction H + *Tt*-GA S1

Note 2. Assignments of lines in groups A, B, C, and D S1

Note 3. Photolysis of *Cc*-$HOCH_2C^{•}O$ **(3)** and *Cc*-$HOCH_2CH_2O^{•}$ **(1)** at 460 and 520 nm S4

Note 4. Assignments of lines in groups A′, B′, and D′ S5

Note 5. Estimates of mixing ratios S7

Note 6. Temporal profiles of the reaction H + *Tt*-GA S7

Table S1. Comparison of Observed and Calculated Vibrational Wavenumbers and IR Intensities of *Cc*-GA and *Tt*-GA S10

Table S2. Variations of Integrated Absorbance for *Cc*-GA, *Tt*-GA, and Reaction Products after Each Step of the H + *Cc*-GA Experiment with GA/Cl_2/*p*-H_2 (1/10/10000) and $[Cc\text{-GA}]_0$ = 203.9 ppm S11

Table S3. Variations of Integrated Absorbance for *Tt*-GA, *Cc*-GA, and Reaction Products after Each Step of the H + *Tt*-GA Experiment with GA/Cl_2/*p*-H_2 (1/10/10000) and $[Cc\text{-GA}]_0$ = 148.6 ppm S12

Table S4. Estimated Mixing Ratios (in ppm) of Observed Species in Each Step of H-deficient and H-rich Experiments of H + *Cc*-GA S13

Table S5. Estimated Mixing Ratios (in ppm) of Observed Species in Each Step of H-deficient and H-rich Experiments of H + *Tt*-GA S14

Table S6. Vertical Excitation Wavelengths and Oscillator Strengths of Electronic Excitations of *Cc*-$HOCH_2C^{•}O$ **(3)** and *Tt*-$HOCH_2C^{•}O$ **(3′)** Predicted with the TD-B3LYP/aug-cc-pVTZ Method S15

Table S7. Vertical Excitation Wavelengths and Oscillator Strengths of Electronic Excitations of *Cc*-$HOCH_2CH_2O^{•}$ **(1)** and *Tt*-$HOCH_2CH_2O^{•}$ **(1′)** Predicted with the TD-B3LYP/aug-cc-pVTZ method S16

Table S8. Integrated Regions of Spectral Lines and Their Corresponding Harmonic Infrared Intensities Employed in the Estimations of Mixing Ratios S17

Figure S1. Geometries of conformers of glycolaldehyde (GA) optimized with the B3LYP/aug-cc-pVTZ method S18

Figure S2. Full-range spectra (except 2750−2250 cm^{-1}) of a GA/Cl_2/*p*-H_2 (1/10/10000) matrix recorded at various stages of the H + *Cc*-GA experiment. S19

Figure S3. Representative spectra of a GA/Cl_2/*p*-H_2 (1/10/10000) matrix after irradiations at 266 nm or 2827 nm S21

Figure S4. Representative spectra of a GA/Cl_2/*p*-H_2 (1/10/10000) matrix recorded at various stages of the H + *Tt*-GA experiment S22

Figure S5. Full-range spectra (except 2750−2250 cm^{-1}) of a GA/Cl_2/*p*-H_2 (1/10/10000) matrix recorded at various stages of the H + *Tt*-GA experiment. S23

Figure S6. Geometries of H-addition and H-induced fragmentation products in reactions H + *Cc*-GA and H + *Tt*-GA optimized with the B3LYP/aug-cc-pVTZ method S25

Figure S7. Geometries of H-abstraction products in reactions H + *Cc*-GA and H + *Tt*-GA optimized with the B3LYP/aug-cc-pVTZ method S26

Figure S8. Geometries of transition states in the reaction H + *Cc*-GA optimized with the B3LYP/aug-cc-pVTZ method S27

Figure S9. Potential-energy scheme of various channels predicted for the reaction H + *Tt*-$HOCH_2C(O)H$ (*Tt*-GA) S28

Figure S10. Geometries of transition states in the reaction H + *Tt*-GA optimized with the B3LYP/aug-cc-pVTZ method S29

Figure S11. Comparison of lines in groups A−D with predicted IR stick spectra of possible H-reaction products of H + *Cc*-GA S30

Figure S12. Vertical excitation UV spectra of various products formed in the reactions of H + *Cc*-GA (A) and H + *Tt*-GA (B) S31

Figure S13. The frontier molecular orbital diagrams of *Cc*-$HOCH_2C^•O$ **(3)** and *Tt*-$HOCH_2C^•O$ **(3′)** S32

Figure S14. The frontier molecular orbital diagrams of *Cc*-$HOCH_2CH_2O^•$ **(1)** and *Tt*- $HOCH_2CH_2O^•$ **(1′)** S33

Figure S15. Comparison of lines in groups A−D, A′, B′, and D′ with predicted IR stick spectra of other H-reaction products of H + *Cc*-GA and H + *Tt*-GA S34

Figure S16. Comparison of lines in groups A′, B′, C, and D′ with predicted IR stick spectra of possible H-reaction products of H + *Tt*-GA S35

Figure S17. Comparison of lines in groups A′, B′, C, and D′ with predicted IR stick spectra of possible H-reaction products of H + *Tt*-GA in different spectral regions S36

Figure S18. Expanded version of traces (a) and (b) of Figure 6: Temporal evolution of mixing ratios of products formed in the reaction H + *Cc*-GA. S37

Figure S19. Potential-energy scheme of H-addition and H-abstraction reactions connecting $C^•H_2OH$, H_2CO, $HC^•O$, and CO S38

Figure S20. Temporal evolution of mixing ratios of *Tt*-GA and products formed in the reaction H + *Tt*-GA. S39

Figure S21. Expanded version of traces (a′) and (b′) of Figure S21: Temporal evolution of mixing ratios of products formed in the reaction H + *Tt*-GA. S40

Supporting References S41

Note 1. PES of the reaction H + *Tt*-GA

Similar to that for the reaction H + *Cc*-glycolaldehyde (GA), the PES for H + *Tt*-GA, as illustrated in Figure S9, was investigated with the CCSD(T)/aug-cc-pVTZ//B3LYP/aug-cc-pVTZ method. Both H abstractions from the methylene and the formyl moieties of *Tt*-GA, resulting in the formation of *Tt*-HOC•HC(O)H **(4′)** and *Tt*-$HOCH_2C^{\bullet}O$ **(3′)**, respectively, are exothermic by 84 and 59 kJ mol^{-1} with similar barriers 22 and 23 kJ mol^{-1}, respectively, whereas the H-abstraction from the hydroxyl moiety, leading to the formation of *t*-$O^{\bullet}CH_2C(O)H$ **(5′)**, is unlikely to occur because this reaction is endothermic by ~6 kJ mol^{-1} and has a large barrier of 60 kJ mol^{-1}. On the other hand, our predictions revealed that the attack of a hydrogen atom on the C−C bond of *Tt*-GA resulted in the bond rupture and formation of $C^{\bullet}H_2OH$ + H_2CO via a barrierless path even though a barrier is expected; more sophisticated theoretical investigations are required to gain deeper understanding for this channel. The H addition to the C atom of the C=O moiety results in the formation of $HOCH_2CH_2O^{\bullet}$ **(1′)** via a barrier of 35 kJ mol^{-1}. Moreover, the H addition to the O atom of the C=O moiety, resulting in the formation of $HOCH_2C^{\bullet}HOH$ **(2′)**, is exothermic by 92 kJ mol^{-1} with a barrier of 52 kJ mol^{-1}, whereas the H addition to the OH moiety, leading to the rupture of the O−H bond to form H_2O + $C^{\bullet}H_2C(O)H$, is the most exothermic, by 133 kJ mol^{-1}, but involves the largest barrier of 98 kJ mol^{-1}; these paths are unlikely to occur under our experimental conditions. The PES depicted in Figure S9b presents further H-abstraction on the HCO moiety of *Tt*-HOC•HC(O)H **(4′)** or on the CH_2 moiety of *Tt*-$HOCH_2C^{\bullet}O$ **(3′)** leading to the formation of the same product HOCHCO **(6)**; both paths are barrierless. Alternatively, H-abstraction from the OH moiety of **(3′)** results in the formation of fragmented products H_2CO + CO via the C−C bond cleavage. The H-addition paths were also indicated; only H + HOCHCO **(6)** paths to form **(3′)** or **(4′)** have small barriers, ~13 and 16 kJ mol^{-1}, respectively. The geometries of all transition states involved in these channels are presented in Figure S10.

Note 2. Assignments of lines in groups A, B, C, and D

Lines in group A depicted in the bottom trace of Figures 5a and S11a, taken from Figures 2c and S2c are compared with the IR stick spectra of **(3)**, **(4)**, **(6)**, and **(1)** according to the vibrational wavenumbers and harmonic IR intensities predicted with the B3LYP/cc-pVTZ method, shown in Figure 5b−5e and S11b−S11e; the harmonic vibrational wavenumbers were scaled as discussed in the Methods section.

Lines in group A agree satisfactorily with the IR stick spectra of *Cc*-$HOCH_2C^{\bullet}O$ **(3)**, Figures 5b and S11b, in terms of vibrational wavenumbers and relative IR intensities; Table 1 compares the observed vibrational wavenumbers and relative IR intensities of lines in group A with the predicted scaled harmonic vibrational wavenumbers and IR intensities of *Cc*-$HOCH_2C^{\bullet}O$ **(3)**. The key structural change from GA to **(3)** is the decreased length of the C=O bond and hence the diminished hydrogen bonding due to the abstraction of the formyl hydrogen. The OH-stretching (ν_1) wavenumber of **(3)**, predicted near 3628 cm^{-1} and observed at 3626.5

cm^{-1}, is greater than that of the hydrogen-bonded OH of *Cc*-GA, 3537.8 cm^{-1}, supporting the diminished H-bonding in **(3)**. The wavenumber of the most intense mode, C=O stretch (ν_4) predicted near 1869 cm^{-1} and observed at 1867.9 cm^{-1}, is much greater than the corresponding value at 1746.6 cm^{-1} of *Cc*-GA, supporting a stronger C=O bond in **(3)** after H abstraction of *Cc*-GA; the predicted C=O bond length decreased from 1.207 to 1.179 Å. The intense line associated with the CO-stretching (ν_9) mode, predicted near 1034 cm^{-1} and observed at 1031.4 cm^{-1}, has wavenumbers slightly smaller than that (1111.0 cm^{-1}) of *Cc*-GA, indicating a weaker C−O bond of **(3)**. Most predicted lines of **(3)** with IR intensity >20 km mol^{-1} were observed, as shown in Table 1. We hence assigned lines in group A to *Cc*-$HOCH_2C^{\bullet}O$ **(3)**; the average absolute deviation between experiments and scaled harmonic vibrational wavenumbers of **(3)** is 5.5 ± 4.3 cm^{-1}.

Lines in group B depicted in Figures 5a and S11a, agree satisfactorily with the IR stick spectrum of *Cc*-HOC$^{\bullet}$HC(O)H **(4)** shown in Figures56c and S11c; Table 2 compares the observed vibrational wavenumbers and relative IR intensities of lines in group B with those predicted for *Cc*-HOC$^{\bullet}$HC(O)H **(4)**. The key structural changes from GA to **(4)** are the reduced strength of the C=O bond and the increased strength of the C−C and C−O bonds due to the delocalization over OCCO. The OH-stretching (ν_1) mode of **(4)**, predicted near 3393 cm^{-1} and observed at 3371.8 cm^{-1}, has a wavenumber much smaller than that of the hydrogen-bonded OH of *Cc*-GA, 3537.8 cm^{-1}, supporting a slightly enhanced H-bonding in **(4)**. The wavenumber of the intense C=O stretch (ν_4) mode, predicted near 1529 cm^{-1} and observed at 1549.5 cm^{-1}, is much smaller than the corresponding value of 1746.6 cm^{-1} of *Cc*-GA, supporting a much weaker C=O bond in **(4)**; the predicted C=O bond length increased from 1.207 to 1.242 Å. The line associated with the CC-stretching (ν_9) mode, predicted near 1002 cm^{-1} and observed at 1002.4 cm^{-1}, has wavenumbers significantly greater than that (859.3 cm^{-1}) of *Cc*-GA, indicating a much stronger C−C bond of **(4)**; the predicted C−C bond length decreased from 1.503 to 1.418 Å. Similarly, the intense line associated with the CO-stretching mode (ν_8, coupled with in-plane OH- and CH-bending modes), predicted near 1185 cm^{-1} and observed at 1176.3 cm^{-1}, has wavenumbers significantly greater than that (1111.0 cm^{-1}) of *Cc*-GA, indicating a stronger C−O bond of **(4)**; the predicted C−O bond length decreased from 1.400 to 1.326 Å. Most predicted lines of **(4)** with IR intensity >20 km mol^{-1} were observed, as summarized in Table 2. Only a line predicted near 2903 cm^{-1}, corresponding to the CH-stretching (ν_3) mode, was not identified due to the interference of the parent and intense lines of HCl. We hence assigned lines in group B to *Cc*-HOC$^{\bullet}$HC(O)H **(4)**; the average absolute deviation between experiments and scaled harmonic wavenumbers of **(4)** is 12.2 ± 11.6 cm^{-1}.

The vibrational wavenumbers and IR intensities of lines in group C, shown in Figures 5a and S11a, agree satisfactorily with the IR stick spectrum predicted for hydroxyketene, HOCHCO **(6)**, shown in Figures 5d and S11d; Table 3 compares the observed vibrational wavenumbers and relative IR intensities of lines in group C with those predicted for HOCHCO **(6)**. The main difference in the structure of **(6)** as compared with those of **(3)** and **(4)** is the presence of the C=C bond with a bond length, 1.318 Å, much shorter than those (1.520 and 1.418 Å) of the C−C bond in **(3)** and **(4)**, respectively. The most intense line corresponding to the characteristic *anti*-symmetric C=C=O stretch (ν_3) was predicted near 2139 cm^{-1} and observed at 2123.6 cm^{-1}; other lines are

much weaker. The OH-stretching (ν_1) mode of **(6)**, predicted near 3602 cm^{-1}, was observed at 3617.0 cm^{-1}, slightly smaller than a value of 3626.5 cm^{-1} of **(3)**. The line corresponding to the CO-stretching mode (ν_6) mode was predicted near 1155 cm^{-1} with significant intensity, but this line could only be tentatively assigned to a line at 1137.4 cm^{-1} because of the interference from the parent absorption line near 1139.0 cm^{-1}. We hence assigned lines in group C to HOCHCO **(6)**; the average absolute deviation between experiments and scaled harmonic wavenumbers of **(6)** is 16.6 ± 4.1 cm^{-1}**.**

Lines in group D are compared with the predicted IR stick spectra of H-reaction products in Figures 5 and S11. These lines agree satisfactorily with the predicted IR stick spectra of *Cc*-$HOCH_2CH_2O^{\bullet}$ **(1),** as presented in Figures 5e and S11e; the experimental results of lines in group D are compared with those predicted for **(1)** in Table 4. The main difference in the structures between **(1)** and *Cc*-GA is that the C=O bond in *Cc*-GA becomes C−O bond in **(1)**, with bond length increased from 1.207 to 1.364 Å. The vibrational wavenumbers for the coupled C−O_H and C−$O^{\bullet}$ stretches (modes ν_{14} and ν_{13}) were predicted to be near 1019 and 1070 cm^{-1} and observed at 991.7 (most intense) and 1071.4 cm^{-1}, respectively. The OH-stretching (ν_1) mode of **(1)**, predicted near 3607 cm^{-1} and observed at 3610.5 cm^{-1}, is slightly smaller than values 3626.5 and 3617.0 cm^{-1} for **(3)** and **(6)**, respectively. The line associated with the $_{O}CH_2$-rocking (ν_{17}) mode was predicted near 765 cm^{-1} and observed at 765.2 cm^{-1}. The $_{HO}CH_2$-symmetric stretch (ν_3) was predicted near 2885 cm^{-1} with significant intensity; it was tentatively assigned to a line at 2905.5 cm^{-1} because of the severe interference of HCl absorption near 2894 cm^{-1} in this region. Other lines having intensity >20 km mol^{-1}, including ν_2, ν_4, ν_7, and ν_{10}, which were predicted near 2966, 2848, 1384, and 1232 cm^{-1}, respectively, could not be positively identified due to the interferences of absorptions of either the parent or the products. We hence assigned lines in group D to *Cc*-$HOCH_2CH_2O^{\bullet}$ **(1)** (Table 4); the average absolute deviation between experiments and scaled harmonic wavenumbers of **(1)** is 10.8 ± 10.2 cm^{-1}.

The destruction of *Cc*-$HOCH_2C^{\bullet}O$ **(3)** at 460 and 520 nm and *Cc*-$HOCH_2CH_2O^{\bullet}$ **(1)** at 460 nm during secondary photolysis agrees well with the UV spectra predicted with the TD-B3LYP/aug-cc-pVTZ method. A description on their photodecomposition is provided in Supporting Information Note 3 (Figures S12−S14). We also compared the observed lines with the predicted IR stick spectra of other possible products, *c*-$O^{\bullet}CH_2C(O)H$ **(5)**, H_2O + $C^{\bullet}H_2C(O)H$, and *Cc*-$HOCH_2C^{\bullet}HOH$ **(2)**, as presented in Figure S15; lines in group A −D agree poorly with those predicted for these species.

In addition to lines in groups A−D, we also observed lines of $C^{\bullet}H_2OH$ at 3651.9, 3165.3, 3038.5, 1457.8, 1332.1, 1179.3, and 1046.9 cm^{-1}, [1] lines of H_2CO at 2782.9 and 1742.6 cm^{-1}, [2] and rovibrational lines of CO around 2143 cm^{-1}.[3] The formation of $C^{\bullet}H_2OH$ and H_2CO is in line with the PES depicted in Figure 5 in which the H-attack to the C atom of the C=O moiety of *Cc*-GA resulted in the rupture of the C−C bond and subsequent formation of $C^{\bullet}H_2OH$ and H_2CO, reaction 7, which was not reported by Álvarez-Barcia et al. in their calculations.[8] Furthermore, the H abstraction on the OH moiety of *Cc*-$HOCH_2C^{\bullet}O$ **(3)** results in the fragmented products H_2CO + CO; this could be one

of the pathways for the formation of H_2CO and CO.

After prolonged irradiation of *Cc*-GA at 3537 cm^{-1} to convert it to *Tt*-GA, the reaction of H atoms with *Tt*-GA produced lines in groups A′, B′, C, and D′**.** The variations of integrated absorbance for these groups of new lines, $C^{\bullet}H_2OH$, H_2CO, $HC^{\bullet}O$, and CO at each step of the H + *Tt*-GA experiment are summarized in Table S3. Following the similar consideration, we assigned lines in groups A′, B′, and C to H-abstraction products *Tt*-$HOCH_2C^{\bullet}O$ **(3′)**, *Tt*-$HOC^{\bullet}HC(O)H$ **(4′)**, and HOCHCO **(6)**, respectively**,** and lines in group D′ to the H-addition product *Tt*-$HOCH_2CH_2O^{\bullet}$ **(1′)**, as presented in Figures S16 and S17 and discussed in Supporting Information Note 4.

Note 3. Photolysis of *Cc*-$HOCH_2C^{\bullet}O$ (3) and *Cc*-$HOCH_2CH_2O^{\bullet}$ (1) at 460 and 520 nm

The intensities of lines of *Cc*-$HOCH_2C^{\bullet}O$ **(3)** decreased upon irradiation at 460 and 520 nm; this photolytic behavior agrees with the TD-B3LYP/aug-cc-pVTZ calculations, which indicate a vertical absorption band near 485 nm for **(3)**, as presented in Figure S12; associated transitions and oscillator strengths are listed in Table S6. In contrast, there is no absorption for the closed-shell GA at wavelength greater than 240 nm. The frontier molecular orbitals of *Cc*-$HOCH_2C^{\bullet}O$ **(3)** illustrated in Figure S13 indicate that the transition near 485 nm for **(3)** corresponds to HOMO(α) → LUMO(α), in which the C−C bonding electron in HOMO(α) is excited to the LUMO(α) with no bonding along the C−C bond. The photodissociation of **(3)** into $C^{\bullet}H_2OH$ and CO observed in range 460−521 nm is consistent with the predictions.

Similarly, observed destruction of *Cc*-$HOCH_2CH_2O^{\bullet}$ **(1)** at 460 nm is consistent with the calculated results showing a vertical absorption band near 471 nm for **(1)**, as represented in Figure S12; associated transitions and oscillator strengths are listed in Table S7. The frontier molecular orbitals illustrated in Figure S14 indicate that the transition near 471 nm for **(1)** corresponds to HOMO−1(β) → LUMO(β) and HOMO(β) → LUMO(β), in which the C−C bonding electron in HOMO(β) is excited to the LUMO(β) with no bonding along the C−C bond. The photodissociation of **(1)** into $C^{\bullet}H_2OH$ and H_2CO near 460 nm is consistent with the predictions. Other products, *Cc*-$HOC^{\bullet}HC(O)H$ **(4)** and HOCHCO **(6)**, have predicted absorptions less than 280 nm, which are close to the absorption of parents; irradiations at 520 and 460 nm dissociate neither **(4)** nor **(6)**, consistent with our experiments.

Note 4. Assignments of lines in groups A′, B′, and D′

Lines in group A' depicted in the lower trace of Figures S16a and S17a, taken from Figures S4c and S5c, are compared with the IR stick spectra of **(3′)**, **(4′)**, **(6)**, and **(1′)** according to scaled harmonic vibrational wavenumbers and harmonic IR intensities predicted with the B3LYP/aug-cc-pVTZ method, shown in Figures S16b−S16e and S17b−S17e, respectively. Lines in group A′ agree satisfactorily with the predicted IR stick spectra of *Tt*-$HOCH_2C^•O$ **(3′)**, Figures S16b and S17b, in terms of vibrational wavenumber and relative IR intensities. Table 1 compares the observed vibrational wavenumbers and relative IR intensities of lines in group A′ with the scaled harmonic vibrational wavenumber and IR intensities of *Tt*-$HOCH_2C^•O$ **(3′)**. The key structural change from *Tt*-GA to **(3′)** due to the abstraction of the formyl hydrogen is the decreased length of the C=O bond. The OH-stretching (ν_1) mode of **(3′)**, predicted near 3654 cm^{-1} and observed at 3638.1 cm^{-1}, has wavenumbers smaller than that (3677.3 cm^{-1}) of *Tt*-GA and slightly larger than that (3626.5 cm^{-1}) of **(3)**, indicating that the O−H bond of **(3′)** is weaker in the former case and stronger in the latter case. The wavenumber of the most intense mode, C=O stretch (ν_4) predicted near 1877 cm^{-1} and observed at 1873.3 cm^{-1}, is much greater than the corresponding value at 1752.0 cm^{-1} of *Tt*-GA, supporting the stronger C=O bond in **(3′)** after H abstraction of *Tt*-GA; the predicted C=O bond length decreased from 1.202 to 1.178 Å. In comparison with **(3)**, the wavenumber is slightly greater, indicating that the C=O bond of **(3′)** is slightly stronger because of the absence of H-bonding. The second-most intense line associated with the CO-stretching (ν_9) mode, predicted near 1076 cm^{-1} and observed at 1073.5 cm^{-1}, has wavenumbers slightly larger than those (1067.7 and 1031.4 cm^{-1}) of *Tt*-GA and **(3)**, indicating a stronger C−O bond of **(3′)** compared to both *Tt*-GA and **(3)**. Most predicted lines of **(3)** with IR intensity >10 km mol^{-1} were observed, as shown in Table 1. We hence assigned lines in group A' to *Tt*-$HOCH_2C^•O$ **(3′)**; the average absolute deviation between experiments and scaled harmonic wavenumbers of **(3')** is 9.2 ± 5.3 cm^{-1}.

Lines in group B′ depicted in the bottom trace of Figures S16a and S17a, taken from Figures S4c and S5c, agree satisfactorily with the IR stick spectrum of *Tt*-$HOC^•HC(O)H$ **(4′)**, Figures S17c and S18c, in terms of vibrational wavenumber and relative IR intensities. Table 2 compares the observed vibrational wavenumbers and relative IR intensities of lines in group B' with the scaled harmonic vibrational wavenumber and IR intensities of *Tt*-$HOC^•HC(O)H$ **(4′)**. Similar to **(4)**, the key structure changes from *Tt*-GA are the reduced C=O bond strength and the increased bond strength of the C−C and C−O bonds due to the delocalization over OCCO. The OH-stretching (ν_1) mode of **(4′)**, predicted near 3645 cm^{-1} and observed at 3629.6 cm^{-1}, has wavenumbers slightly smaller than that (3677.3 cm^{-1}) of *Tt*-GA and significantly larger than that (3371.8 cm^{-1}) of **(4)**, indicating that the O−H bond of **(3′)** is weaker than that of *Tt*-GA because of the H abstraction on CH_2 of *Tt*-GA and

stronger than that of **(4)** because of the absence of the intramolecular H-bonding. The wavenumber of the C=O stretch (ν_4) predicted near 1557 cm^{-1} and observed at 1554.8 cm^{-1}, is much smaller than the corresponding value of 1752.0 cm^{-1} of *Tt*-GA, supporting a weaker C=O bond in **(4′)**; the predicted C=O bond length increased from 1.202 to 1.235 Å. The line associated with the CC-stretching mode (ν_9, coupled with the OH bend), predicted near 1069 cm^{-1} and observed at 1074.7 cm^{-1}, has wavenumbers greater than that (1000.2 cm^{-1}) of *Tt*-GA, indicating a stronger C−C bond of **(4′)**; the predicted C−C bond length decreased from 1.513 to 1.414 Å. The intense line corresponding to the CO-stretching mode (ν_7, coupled with in-plane OH- and CH-bend) and another line corresponding to the CO-stretching mode (ν_8, coupled with in-plane CH-bending), predicted near 1231 and 1222 cm^{-1} and observed at 1245.9 and 1214.4 cm^{-1}, respectively have wavenumbers significantly greater than that (1067.7 cm^{-1}) of *Tt*-GA, indicating a stronger C−O bond of **(4′)**; the predicted C−O bond length decreased from 1.418 to 1.346 Å. Most predicted lines of **(4′)** with IR intensity >15 km mol^{-1} were observed, as shown in Table 2. A predicted line near 2840 cm^{-1}, corresponding to the CH-stretching (ν_3) mode, was not observed due to the interference of the parent. We hence assigned lines in group B' to *Tt*-HOC$^{\bullet}$HC(O)H **(4′)**; the average absolute deviation between experiments and scaled harmonic wavenumbers of **(3)** is 9.9 ± 5.6 cm^{-1}.

Lines in group D' depicted in the lower trace of Figures S16a and S17a, taken from Figures S4c and S5c, agree satisfactorily with the IR stick spectrum of *Tt*-$HOCH_2CH_2O^{\bullet}$ **(1′)**, Figures S16e and S17e, in terms of vibrational wavenumber and relative IR intensities. Table 4 compares the observed vibrational wavenumbers and relative IR intensities of lines in group D' with the scaled harmonic vibrational wavenumber and IR intensities of *Tt*-$HOCH_2CH_2O^{\bullet}$ **(1′)**. The crucial geometry difference between **(1′)** and *Tt*-GA is the the C=O bond in *Tt*-GA becomes C−O bond in **(1′)**, with bond length increased from 1.202 to 1.359 Å. The vibrational wavenumbers for the coupled C−OH and C−O$^{\bullet}$ stretches (ν_{13} and ν_{14}) were predicted to be near 1059 and 1039 cm^{-1} and observed at 1061.6 and 1036.4 (most intense) cm^{-1}, respectively. Similarly, the OH-stretching (ν_1) mode of **(1′)**, predicted near 3660 cm^{-1} and observed at 3656.3 cm^{-1}, is slightly greater than a value 3610.5 cm^{-1} for **(1)** because of the absence of intramolecular H-bonding. The lines associated with the CH_2 bend (ν_9, ν_{10}, and ν_{12} coupled with the CH_2 twist and the CCH bend), predicted near 1356, 1284, and 1206 cm^{-1}, were observed at 1375.5, 1293.6, and 1210.9 cm^{-1}. Other lines having intensity >15 km mol^{-1} include CH_2-symmetric stretches (ν_3 and ν_5) and CH_2-asymmetric stretch (ν_2) predicted near 2892, 2794, and 2926 cm^{-1}, respectively, could not be definitely identified due to the interferences of absorptions of either the parent or products. We hence assigned lines in group D' to *Tt*-$HOCH_2CH_2O^{\bullet}$ **(1′)**; the average absolute deviation between experiments and scaled harmonic wavenumbers of **(1′)** is 7.6 ± 5.9 cm^{-1}. We also compared the observed lines in groups A', B', C, and D' with the predicted

IR stick spectra of other possible products, *t*-O•CH$_2$C(O)H **(5′)**, H_2O + C•H$_2$C(O)H, and *Tt*-HOCH$_2$C•HOH **(2′)**, as presented in Figure S14; the observed lines agree poorly with those predicted for these species**.**

Note 5. Estimates of mixing ratios

For the estimation of mixing ratios of species involved during the reaction H + GA using the method described by Tam and Fajardo, two parameters, IR optical pathlength of the matrix and harmonic IR intensities predicted with the B3LYP/aug-cc-pVTZ method, were employed.[4]

$$x = \frac{2.303 \int \log_{10}(I/I_0) d\nu}{\varepsilon\, l} \times V_m \times 10^6, \qquad (1)$$

in which *x* is the mixing ratio in ppm, *ε* is the absorption coefficient in cm mol^{-1}, $\int \log_{10}(I/I_0)d\nu$ is the observed integrated absorbance of a specific line, *l* (in cm) is the IR absorption pathlength through solid *p*-H_2, and V_m (= 23.16 cm^3 mol^{-1}) is the molar volume of solid *p*-H_2.[5] The IR optical pathlength *l* was determined from the IR absorption of *p*-H_2, as described by Fajardo,[6]

$$l = 5.759 \times \int A_{1167}\, d\nu - 0.0075 \qquad (2)$$

This equation represents the linear fit between *l* against $\int A_{1167}\, d\nu$ (the integrated absorbance of the band at 1167 cm^{-1}, a pure rotation line of *p*-H_2 matrix) over a period of deposition time. Due to uncertainties in the calculated IR intensities of each species, the variations in mixing ratios between different species might have uncertainties as large as a factor of two, but the ratios of mixing ratios of a specific species in various stages of experiments are considered to be reliable. To alleviate potential errors associated with predicted IR intensities, we adopted an approach of averaging values derived from several spectral lines whenever possible. The integrated regions of each species used for estimation of mixing ratios are listed in Table S8. The standard deviations in fitting various lines of each species are listed in the caption. The mixing ratio of HCl produced immediately after UV/IR irradiation was employed to estimate the initial mixing ratio of H atoms, assuming that each Cl atom generated one H atom after reacting with H_2, that is, Cl + H_2 (ν = 1) → HCl + H. In darkness, the mixing ratio of HCl increases because of secondary reaction of H atoms with some unreacted Cl atoms. In darkness, the thickness of the matrix at the initial stage was used; the decrease in thickness of the matrix due to evaporation was not accounted for in the calculations of mixing ratio.

Note 6. Temporal profiles of the reaction H + *Tt*-GA

To investigate the tunnelling reaction in darkness for the H + *Tt*-GA reaction, in separate experiments, as much *Cc*-GA as possible was converted to *Tt*-GA by IR irradiation for 15 h before its UV photolysis for 30 min and IR irradiation for 60 min; subsequently, the matrix was maintained in darkness for 10 h. The IR spectra were recorded every 30 min to provide estimated mixing ratios

of each species, as depicted in Figure S20 (expanded version of temporal evolution of products is illustrated in Figure S21). Two different conditions were studied; results of the H-deficient experiment, with $[H]_0/[Tt\text{-GA}] \approx 1.1$ and $[Tt\text{-GA}]_0 = 107$ ppm, are presented in Figure S20a′, whereas those of the H-rich experiment, with $[H]_0/[Tt\text{-GA}] \approx 4.1$ and $[Tt\text{-GA}]_0 = 131$ ppm, are presented in Figure S20b′. The estimated mixing ratios of observed species in each step of both experiments of *Tt*-GA are listed in Table S5. The behaviours of these species are similar to those of the corresponding species in experiments with H + *Cc*-GA, except that the yields appeared to be smaller.

As discussed in the main text for H + *Cc*-GA, the sum of mixing ratios of all products after IR irradiation, 6 and 51 ppm for the H-deficient and the H-rich experiment, respectively, agree well with the loss of *Tt*-GA, 6 and 52 ppm in two experiments, respectively, although the estimated mixing ratios might have large uncertainties. However, in darkness the sum of mixing ratios of the increases of all products of H + *Tt*-GA, 0.5 and 0.8 ppm, are slightly smaller than the loss of *Tt*-GA, 0.6 and 1.0 ppm, respectively; the evaporation of the matrix during darkness for 10 h might be the reason. The mixing ratios of *Cc*-GA and its products were not considered in this case because of their small mixing ratios hence large uncertainties. No apparent *Tt* → *Cc* back conversion upon UV/IR irradiation was observed.

In darkness, **(3′)** and **(6)** increased with time, whereas **(4′)** decreased with time, indicating that, the reaction of H with **(4′)** to form **(6)** is slightly more facile than that of H with *Tt*-GA to form **(4′)**, in agreement with the PES showing that the former reaction is barrierless whereas the latter has a barrier ~22 kJ mol^{-1}. The ratios of the decrease of **(4′)** and the increase of **(6)** relative to the decrease of *Cc*-GA in the H-deficient experiment are ~73% those in the H-rich experiment, also supporting that, with more H atoms in the H-rich experiments, the second H-abstraction path became more important. In the H-rich experiment, both **(4′)** and **(6)** appeared to be approaching to a constant value, indicating that H-addition reaction H + **(6)** to form back **(4′)** became more important at the later stage.

The variations of the mixing ratios of $C^{\bullet}H_2OH$, H_2CO, $HC^{\bullet}O$, and CO in darkness are small (<0.1 ppm in the H-deficient experiment and <0.2 ppm in the H-rich experiment); the mixing ratios of H_2CO and $HC^{\bullet}O$ are nearly constant, that of $C^{\bullet}H_2OH$ decreased over time, whereas that of CO increased by a similar amount. This observation seems to indicate that the reaction of H + $C^{\bullet}H_2OH$ eventually produced CO. No obvious production of CH_3OH was observed, but this might be due to interference.

During IR irradiation, the variations of the mixing ratios of these species are quite different from those in darkness, presumably because the H atoms produced during IR irradiation had more energy than those in the tunnelling reactions in darkness and the nearest-neighbor reaction played a more important role. Because of the exothermicity of the reaction $Cl + H_2 (v = 1) \rightarrow HCl + H$ is ~45 kJ

mol^{-1},[7] one would expect that H reactions with a larger barrier or even with slight endothermicity might occur during IR irradiation, but not in darkness. In the H-deficient experiment after IR irradiation, the ratios of production of **(4′)**, **(3′)**, **(6)**, and **(1′)** relative to the loss of *Tt*-GA are approximately 0.19, 0.14, 0.11, and 0.05, respectively, whereas those for the summation of $C^{\bullet}H_2OH$, H_2CO, $HC^{\bullet}O$, and CO relative to the loss of *Tt*-GA are 0.40, respectively. In the H-rich experiment after IR irradiation, the ratios of production of **(4′)**, **(3′)**, **(6)**, and **(1′)** relative to the loss of *Tt*-GA are approximately 0.15, 0.13, 0.14, and 0.12, respectively, whereas those for the summation of $C^{\bullet}H_2OH$, H_2CO, $HC^{\bullet}O$, and CO are 0.50. In the H-deficient experiment, while in darkness, the ratios of destruction of **(4′)** and production of **(3′)**, **(6)**, and **(1′)** relative to the loss of *Tt*-GA due to H reactions are approximately 0.33, 0.67, 0.33, and 0.16, respectively; whereas, in H-rich experiment, the ratios of destruction of **(4′)** and production of **(3′)**, **(6)**, and **(1′)** relative to the loss of *Tt*-GA due to H reactions are approximately 0.46, 0.70, 0.46, and 0.18, respectively. The significantly larger fraction of the production of **(6)** and **(4′)** than **(3′)** during IR irradiation as compared with those in darkness might imply that reaction 4′ that produced **(4′)** has a larger barrier than reaction 3′ that produced **(3′)**, which is in contrast to the quantum-chemical predictions predicted with the CCSD(T)/aug-cc-pVTZ method, but consistent with those predicted with the B3LYP/aug-cc-pVTZ method in this work and the MPWB1K/def2-TZVP method reported by Álvarez-Barcia et al.[8]

Table S1. Comparison of Observed and Calculated Vibrational Wavenumbers and IR Intensities of *Cc*-GA and *Tt*-GA

		Cc-GA					*Tt*-GA				
		B3LYP /aug-cc-pVTZ			*p*-H_2		B3LYP /aug-cc-pVTZ			*p*-H_2	
mode	sym.	harm.[a] /cm^{-1}	int. /[b]	anharm.[c] /cm^{-1}	ν^d /cm^{-1}	ν^e /cm^{-1}	harm.[a] /cm^{-1}	int. /[b]	anharm.[c] /cm^{-1}	ν^f /cm^{-1}	ν^e /cm^{-1}
ν_1	A'	3537	64	3524	3538.0	3537.8	3665	51	3670	3677.4	3677.3
ν_2	A'	2870	62	2816	2849.5	2851.3	2886	33	2835	2826.0	2894.3
ν_3	A'	2823	63	2792	2837.2	2837.5	2808	74	2789	2815.0	2815.0
ν_4	A'	1747	159	1766	1749.7	1746.6	1767	167	1780	1752.1	1752.0
ν_5	A'	1439	20	1425	1424.0	1427.0	1448	14	1451		1438.4
ν_6	A'	1401	37	1386	1408.6	1403.5	1397	2	1388		
ν_7	A'	1363	24	1359	1363.7	1363.8	1350	10	1344	1352.2	1352.8
ν_8	A'	1266	48	1257	1269.6	1269.6	1198	78	1220	1203.8	1204.4
ν_9	A'	1108	83	1096	1111.1	1111.0	1061	76	1047	1067.7	1067.7
ν_{10}	A'	855	52	841	862.0	859.3	977	46	973	1000.1	1000.2
ν_{11}	A'	757	10	744	750.4	751.8	338	16	329		
ν_{12}	A'	295	23	268	281.9[f]		102	27	106		
ν_{13}	A″	2879	16	2834	2882.0	2882.2	2916	16	2878	2919.0	2919.6
ν_{14}	A″	1228	2	1220	1229.0[f]		1223	0	1193		
ν_{15}	A″	1086	0	1080			1093	2	1089		
ν_{16}	A″	718	0	707			733	0	718		
ν_{17}	A″	414	87	367	365.0[f]		548	6	638		
ν_{18}	A″	238	3	224	216.5[f]		232	114	185		

[a]Harmonic vibrational wavenumber scaled according to 0.9619 x + 24.6 for wavenumbers <2500 cm^{-1} and 0.9200 x + 133.4 for wavenumbers >2500 cm^{-1}. [b]In unit of km mol^{-1}. [c]Anharmonic vibrational wavenumber. [d]Ceponkus et al. *J. Chem. Phys.* **2010**, *133* (9), 094502, unless noted. [e] This work. [f]Chin, W. et al. *J. Chem. Phys.* **2014**, *140* (22), 224319.

Table S2. Variations of Integrated Absorbance of *Cc*-GA, *Tt*-GA, and Reaction Products after Each Step of the H + *Cc*-GA Experiment with GA/Cl_2/*p*-H_2 (1/10/10000) and [*Cc*-GA]$_0$ = 203.9 ppm

Species	experimental steps					
	deposition[a]	380 nm[b]	IR[c]	darkness[c]	520 nm[c]	460 nm[c]
Cc-GA	~97%	~95%	−55%	−2%	−1%	~0%
Tt-GA	~3%	~3%	+621%	−30%	−1%	~0%
A, *Cc*-$HOCH_2C^{\bullet}O$ **(3)**	-	<1%	-	+32%	–65%	–30%
B, *Cc*-$HOC^{\bullet}HC(O)H$ **(4)**	-	<1%	-	–15%	~0%	~0%
D, *Cc*-$HOCH_2CH_2O^{\bullet}$ **(1)**	-	-	-	+6%	~0%	–11%
C, HOCHCO **(6)**	-	-	-	+21%	~0%	~0%
$C^{\bullet}H_2OH$	-	<1%	-	–6%	+52%	+43%
H_2CO	-	<1%	-	–2%	–6%	–5%
$HC^{\bullet}O$	-	<1%	-	–2%	–5%	–4%
CO	-	<1%	-	+5%	+18%	+11%

[a]The percentage of the total GA after deposition. [b]The percentage with respect to the total GA after irradiation at 380 nm for 30 min. [c]The variations of GA are with respect to those after irradiation at 380 nm and those of the new species are with respect to those after IR irradiation.

Table S3. Variations of Integrated Absorbance of *Tt*-GA, *Cc*-GA, and Reaction Products after Each Step of the H + *Tt*-GA Experiment with GA/Cl_2/*p*-H_2 (1/10/10000) and [*Cc*-GA]$_0$ = 148.6 ppm

species	experimental steps					
	deposition[a]	2827 nm[b]	380 nm[c]	IR[d]	darkness[d]	520 nm[d]
Cc-GA	~98%	~14%	~14%	−10%	−4%	−1%
Tt-GA	~2%	~86%	~85%	−40%	−1%	−1%
A′, *Tt*-$HOCH_2C^•O$ **(3′)**	-	-	<1%	-	+11%	–45%
B′, *Tt*-$HOC^•HC(O)H$ **(4′)**	-	-	<1%	-	–7%	~0%
D′, *Tt*-$HOCH_2CH_2O^•$ **(1′)**	-	-	~0%	-	+5%	~0%
C, HOCHCO **(6)**	-	-	~0%	-	+20%	~0%
$C^•H_2OH$	-	-	<1%	-	–5%	+48%
H_2CO	-	-	<1%	-	–2%	–6%
$HC^•O$	-	-	<1%	-	–2%	–5%
CO	-	-	<1%	-	+4%	+15%

[a]The percentage of the total GA after deposition. [b]The percentage of the total GA after irradiation at 2827 nm for 15 h. [c]The percentage with respect to the total GA after irradiation at 380 nm for 30 min. [d]The variations of GA are with respect to those after irradiation at 380 nm and those of the new species are with respect to those after IR irradiation.

Table S4. Estimated Mixing Ratios (in ppm) of Observed Species after Each Step of H-deficient and H-rich Experiments of H + *Cc*-GA

(a) H-deficient experiment[a]

	Cc-GA	$HOCH_2C^{\bullet}O$ *Cc*-, **(3)**	$HOC^{\bullet}HC(O)H$ *Cc*-, **(4)**	HOCHCO **(6)**	$HOCH_2CH_2O^{\bullet}$ *Cc*-, **(1)**	$C^{\bullet}H_2OH$	H_2CO	$HC^{\bullet}O$	CO	*Tt*-GA	$HOCH_2C^{\bullet}O$ *Tt*-, **(3′)**	$HOC^{\bullet}HC(O)H$ *Tt*-, **(4′)**	$HOCH_2CH_2O^{\bullet}$ *Tt*-, **(1′)**
Deposition	143.4	0.0	0.0	0.0	0.0	0.0	0.0	0.0	0.0	4.3	0.0	0.0	0.0
UV	141.5	0.0	0.1	0.0	0.0	0.5	0.4	0.3	0.0	4.5	0.0	0.0	0.0
IR	110.1	4.1	5.9	4.4	1.2	1.7	1.5	1.3	0.9	12.6	1.2	1.0	0.2
Darkness (10 h)	107.4	6.1	5.0	5.2	1.6	1.4	1.5	1.3	1.1	12.1	1.4	0.8	0.2

(b) H-rich experiment[a]

	Cc-GA	$HOCH_2C^{\bullet}O$ *Cc*-, **(3)**	$HOC^{\bullet}HC(O)H$ *Cc*-, **(4)**	HOCHCO **(6)**	$HOCH_2CH_2O^{\bullet}$ *Cc*-, **(1)**	$C^{\bullet}H_2OH$	H_2CO	$HC^{\bullet}O$	CO	*Tt*-GA	$HOCH_2C^{\bullet}O$ *Tt*-, **(3′)**	$HOC^{\bullet}HC(O)H$ *Tt*-, **(4′)**	$HOCH_2CH_2O^{\bullet}$ *Tt*-, **(1′)**
Deposition	203.9	0.0	0.0	0.0	0.0	0.0	0.0	0.0	0.0	5.6	0.0	0.0	0.0
UV	199.3	0.1	0.3	0.1	0.0	1.4	1.1	1.0	0.1	5.8	0.1	0.0	0.0
IR	88.9	11.7	15.4	8.9	5.3	10.8	9.3	8.5	6.6	36.0	3.7	2.9	0.9
Darkness (10 h)	84.2	15.4	13.1	11.1	5.8	10.3	9.2	8.5	7.1	34.3	4.5	2.5	1.0

[a]The standard deviations in averaging mixing ratios from various lines are: *Cc*-GA: 4 %; *Tt*-GA: 9 %; *Cc*-$HOC^{\bullet}HC(O)H$ **(4)**: 25 %; *Tt*-$HOC^{\bullet}HC(O)H$ **(4′)**: 27 %; *Cc*-$HOCH_2C^{\bullet}O$ **(3)**: 24 %; *Tt*-$HOCH_2C^{\bullet}O$ **(3′)**: 18 %; *Cc*-$HOCH_2CH_2O^{\bullet}$ **(1)**: 23 %; *Tt*-$HOCH_2CH_2O^{\bullet}$ **(1′)**: 42 %.

Table S5. Estimated Mixing Ratios (in ppm) of Observed Species after Each Step of H-deficient and H-rich Experiments of H + *Tt*-GA

	Cc-GA	*Tt*-GA	$HOCH_2C^{\bullet}O$ *Tt*-, **(3′)**	$HOC^{\bullet}HC(O)H$ *Tt*-, **(4′)**	HOCHCO **(6)**	$HOCH_2CH_2O^{\bullet}$ *Tt*-, **(1′)**	$C^{\bullet}H_2OH$	H_2CO	$HC^{\bullet}O$	CO	$HOCH_2C^{\bullet}O$ *Cc*-, **(3)**	$HOC^{\bullet}HC(O)H$ *Cc*-, **(4)**	$HOCH_2CH_2O^{\bullet}$ *Cc*-, **(1)**
(a) H-deficient experiment[a]													
Deposition	126.2	4.1	0.0	0.0	0.0	0.0	0.0	0.0	0.0	0.0	0.0	0.0	0.0
2827nm	24.2	106.8	0.0	0.0	0.0	0.0	0.0	0.0	0.0	0.0	0.0	0.0	0.0
UV	24.0	106.3	0.0	0.0	0.0	0.0	0.0	0.0	0.0	0.0	0.0	0.0	0.0
IR	23.6	99.9	0.9	1.2	0.7	0.3	0.9	0.7	0.6	0.4	0.3	0.2	0.1
Darkness (10 h)	23.2	99.3	1.3	1.0	0.9	0.4	0.7	0.7	0.6	0.5	0.3	0.2	0.1
(b) H-rich experiment[a]													
	Cc-GA	*Tt*-GA	$HOCH_2C^{\bullet}O$ *Tt*-, **(3′)**	$HOC^{\bullet}HC(O)H$ *Tt*-, **(4′)**	HOCHCO **(6)**	$HOCH_2CH_2O^{\bullet}$ *Tt*-, **(1′)**	$C^{\bullet}H_2OH$	H_2CO	$HC^{\bullet}O$	CO	$HOCH_2C^{\bullet}O$ *Cc*-, **(3)**	$HOC^{\bullet}HC(O)H$ *Cc*-, **(4)**	$HOCH_2CH_2O^{\bullet}$ *Cc*-, **(1)**
Deposition	148.6	3.3	0.0	0.0	0.0	0.0	0.0	0.0	0.0	0.0	0.0	0.0	0.0
2827nm	20.9	131.1	0.0	0.0	0.0	0.0	0.0	0.0	0.0	0.0	0.0	0.0	0.0
UV	20.7	128.5	0.1	0.1	0.0	0.0	0.8	0.6	0.5	0.0	0.0	0.0	0.0
IR	18.6	76.9	6.9	7.7	7.2	0.6	7.0	6.5	6.3	6.0	0.7	1.5	0.3
Darkness (10 h)	17.8	75.9	7.5	7.2	7.7	0.8	6.6	6.5	6.3	6.2	0.6	1.8	0.4

[a]The standard deviations in averaging mixing ratios from various lines are: *Cc*-GA: 4 %; *Tt*-GA: 9 %; *Cc*-$HOC^{\bullet}HC(O)H$ **(4)**: 25 %; *Tt*-$HOC^{\bullet}HC(O)H$ **(4′)**: 27 %; *Cc*-$HOCH_2C^{\bullet}O$ **(3)**: 24 %; *Tt*-$HOCH_2C^{\bullet}O$ **(3′)**: 18 %; *Cc*-$HOCH_2CH_2O^{\bullet}$ **(1)**: 23 %; *Tt*-$HOCH_2CH_2O^{\bullet}$ **(1′)**: 42 %.

Table S6. Vertical Excitation Wavelengths and Oscillator Strengths of Electronic Excitations of *Cc*-$HOCH_2C^{•}O$ (3) and *Tt*-$HOCH_2C^{•}O$ (3′) Predicted with the TD-B3LYP/aug-cc-pVTZ Method

excitation state	*Cc*-$HOCH_2C^{•}O$ **(3)**		*Tt*-$HOCH_2C^{•}O$ **(3′)**		approximate assignment
	wavelength / nm	oscillator strength f	wavelength / nm	oscillator strength f	
1	485.3	0.0017	521.5	0.0009	HOMO(α)→LUMO(α)
2	273.6	0.0022	278.4	0.0195	HOMO(α)→LUMO+1(α)
3	252.2	0.0098	251.6	0.0000	HOMO(β)→LUMO(β)
4	240.2	0.0302	248.3	0.0101	HOMO(α)→LUMO+2(α)
5	223.1	0.0118	230.2	0.0218	HOMO(α)→LUMO+3(α)
6	213.7	0.0140	221.5	0.0476	HOMO(α)→LUMO+4(α)
7	202.9	0.0033	199.1	0.0168	HOMO−2 (β)→LUMO(β)
					HOMO−1 (β)→LUMO(β)
8	198.5	0.0037	210.1	0.0023	HOMO−1(α)→LUMO(α)
9	193.4	0.0089	189.8	0.0005	HOMO−3 (β)→LUMO(β)[a]
					HOMO−1 (β)→LUMO(β)[a]
					HOMO−1(α)→LUMO+1(α)[b]
10	190.6	0.0048	193.7	0.0004	HOMO(α)→LUMO+5(α)
11	186.1	0.0116	191.7	0.0075	HOMO(β)→LUMO+1(β)
12	183.2	0.0017	187.4	0.0105	HOMO(α)→LUMO+6(α)

[a]Transition associated with *Cc*-$HOCH_2C^{•}O$ **(3)**. [b]Transition associated with *Tt*-$HOCH_2C^{•}O$ **(3′)**.

Table S7. Vertical Excitation Wavelengths and Oscillator Strengths of Electronic Excitations of *Cc*-$HOCH_2CH_2O^•$ (1) and *Tt*-$HOCH_2CH_2O^•$ (1′) Predicted with the TD-B3LYP/aug-cc-pVTZ method

excitation state	*Cc*-$HOCH_2CH_2O^•$ **(1)**		*Tt*-$HOCH_2CH_2O^•$ **(1′)**		approximate assignment
	wavelength / nm	oscillator strength *f*	wavelength / nm	oscillator strength *f*	
1	2839.1	0.0003	3078.4	0.0001	HOMO(β)→LUMO(β)
2	470.5	0.0049	413.2	0.0004	HOMO−1(β)→LUMO(β)
					HOMO(β)→LUMO(β) [a]
3	319.7	0.0008	282.5	0.0007	HOMO−2(β)→LUMO(β)
4	267.4	0.0007	216.7	0.0446	HOMO−3(β)→LUMO(β)
5	210.9	0.0322	259.9	0.0001	HOMO−2(β)→LUMO(β)
6	195.8	0.0010	194.8	0.0003	HOMO(β)→LUMO+1(β)
7	190.0	0.0065	192.1	0.0021	HOMO(α)→LUMO(α)
					HOMO−1(β)→LUMO+1(β)[b]
8	185.9	0.0043	185.9	0.0016	HOMO(β)→LUMO+2(β)
9	181.7	0.0052	185.8	0.0006	HOMO−3(β)→LUMO(β)
10	180.6	0.0185	181.3	0.0004	HOMO(α)→LUMO+1(α)
11	177.5	0.0058	174.0	0.0142	HOMO(β)→LUMO+3(β)
12	174.4	0.0066	199.7	0.0000	HOMO−1(β)→LUMO+1(β)

[a]Transition associated with *Cc*-$HOCH_2CH_2O^•$ **(1)**. [b]Transition associated with *Tt*- $HOCH_2CH_2O^•$ **(1′)**.

Table S8. Integrated Regions of Spectral Lines and Their Corresponding Harmonic Infrared Intensities Employed in the Estimations of Mixing Ratios

species	ν_i	mode description	intensity[a] / km mol^{-1}	range / cm^{-1}	$\int A$
Cc-GA	ν_1	ν OH	64	3560.3–3522.3	1.110[b]
Cc-GA	ν_7	ω CH_2/δ COH	24	1368.1–1359.8	0.383[b]
Cc-GA	ν_9	ν CO/δ COH	83	1120.9–1099.3	1.385[b]
Tt-GA	ν_1	ν OH	51	3681.7–3672.0	0.689[c]
Tt-GA	ν_9	ν CO	76	1075.5–1064.3	1.211[c]
Tt-GA	ν_{10}	ν CC/δ COH	46	1007.7–996.1	0.635[c]
Cc-HOC$^•$HC(O)H **(4)**	ν_4	ν C=O/δ CH/δ OH (ip)	34	1551.8–1547.9	0.067[b]
Cc-HOC$^•$HC(O)H **(4)**	ν_5	ν CC/δ CH (ip)/ δ OH (ip)	58	1494.7–1491.5	0.179[b]
Cc-HOC$^•$HC(O)H **(4)**	ν_8	ν CO/δ CH (ip)	140	1177.3–1174.2	0.304[b]
Tt-HOC$^•$HC(O)H **(4′)**	ν_4	ν C=O/δ CH/δ OH (ip)	34	1556.6–1552.2	0.035[c]
Tt-HOC$^•$HC(O)H **(4′)**	ν_5	ν CC/δ CH (ip)/ δ OH (ip)	58	1489.7–1486.3	0.091[c]
Tt-HOC$^•$HC(O)H **(4′)**	ν_7	δ OH (ip)/δ CH (ip)	231	1248.9–1242.7	0.411[c]
Cc-$HOCH_2C^•O$ **(3)**	ν_3	ν C=O	120	1870.5–1865.7	0.218[b]
Cc-$HOCH_2C^•O$ **(3)**	ν_7	ν CO	144	1033.8–1029.3	0.370[b]
Tt-$HOCH_2C^•O$ **(3′)**	ν_3	ν C=O	130	1875.7–1870.3	0.224[b]
Tt-$HOCH_2C^•O$ **(3′)**	ν_6	ω CH_2 /δ COH	96	1186.9–1183.9	0.115[c]
Tt-$HOCH_2C^•O$ **(3′)**	ν_7	ν CO	106	1075.3–1072.6	0.149[c]
HOCHCO **(6)**	ν_3	ν C=C=O	506	2129.5–2117.7	0.344[b]
Cc-$HOCH_2CH_2O^•$ **(1)**	ν_{10}	ν CO	141	996.4–988.8	0.161[b]
Cc-$HOCH_2CH_2O^•$ **(1)**	ν_{12}	γ CH_2	23	771.8–768.1	0.019[b]
Tt-$HOCH_2CH_2O^•$ **(1′)**	ν_7	ω CH_2/δ COH	34	1297.5–1290.8	0.005[c]
Tt-$HOCH_2CH_2O^•$ **(1′)**	ν_8	ω CH_2/δ COH	50	1211.6–1210.0	0.004[c]

[a] Calculated with the B3LYP/aug-cc-pVTZ method. [b] Integrated area of the species from the spectrum (Figures 3c and S2c) recorded after IR irradiation of the reaction H + *Cc*-GA. [c] Integrated area of the species from the spectrum (Figures S4c and S5c) recorded after IR irradiation of the reaction H + *Tt*-GA.

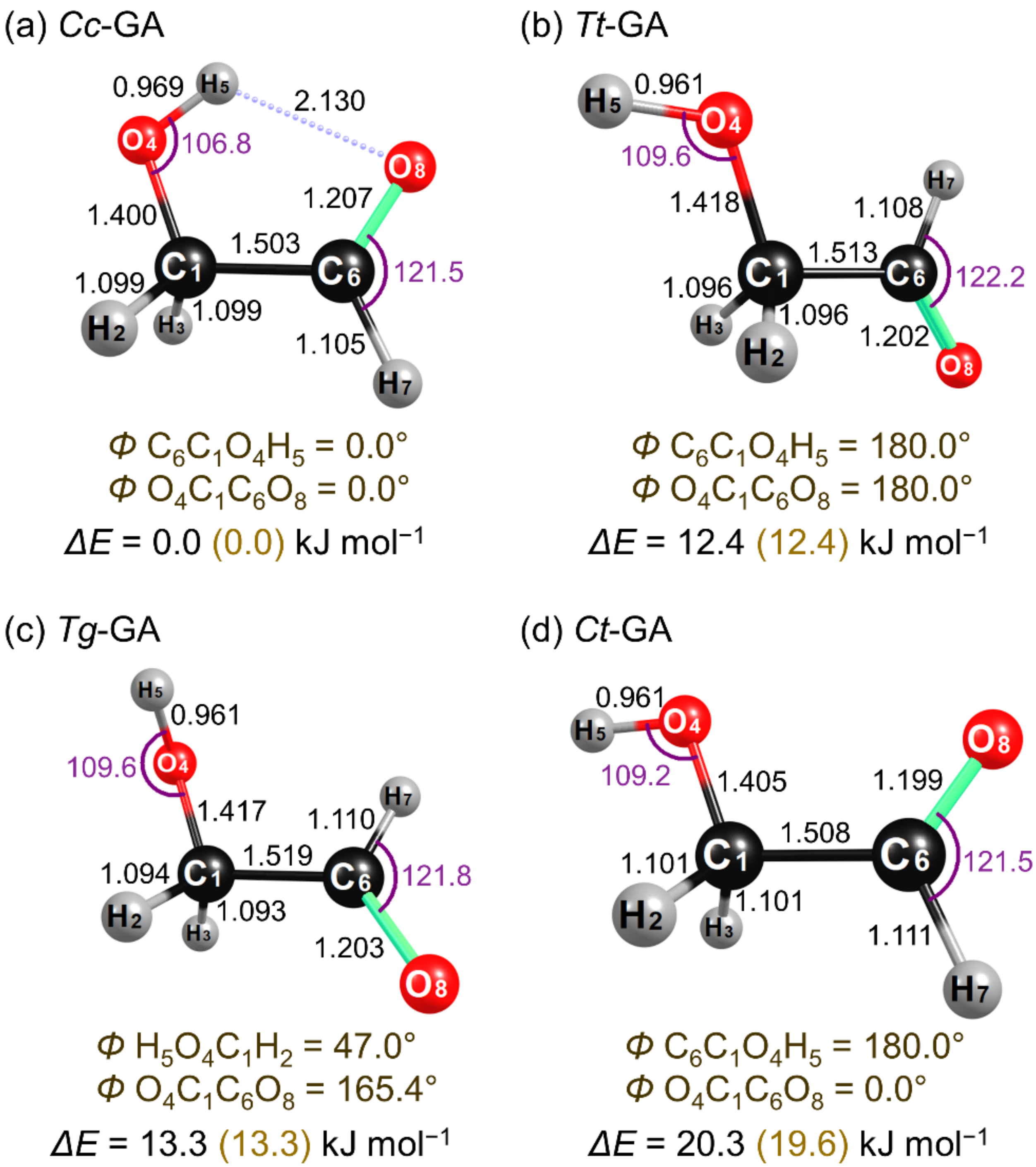

Figure S1. Geometries of conformers of glycolaldehyde (GA) optimized with the B3LYP/aug-cc-pVTZ method. (a) *Cis-cis* glycolaldehyde (*Cc*-GA), (b) *Trans-trans* glycolaldehyde (*Tt*-GA), (c) *Trans-gauche* glycolaldehyde (*Tg*-GA), and (d) *Cis-trans* glycolaldehyde (*Ct*-GA). Bond lengths (black) are in angstrom (Å); bond angles (purple) and dihedral angles Φ are in degrees (°). Relative energies (in kJ mol^{-1}) calculated with the CCSD(T)/aug-cc-pVTZ//B3LYP/aug-cc-pVTZ method (black) are listed; those with the B3LYP/aug-cc-pVTZ method (olive) are listed in parentheses for comparison.

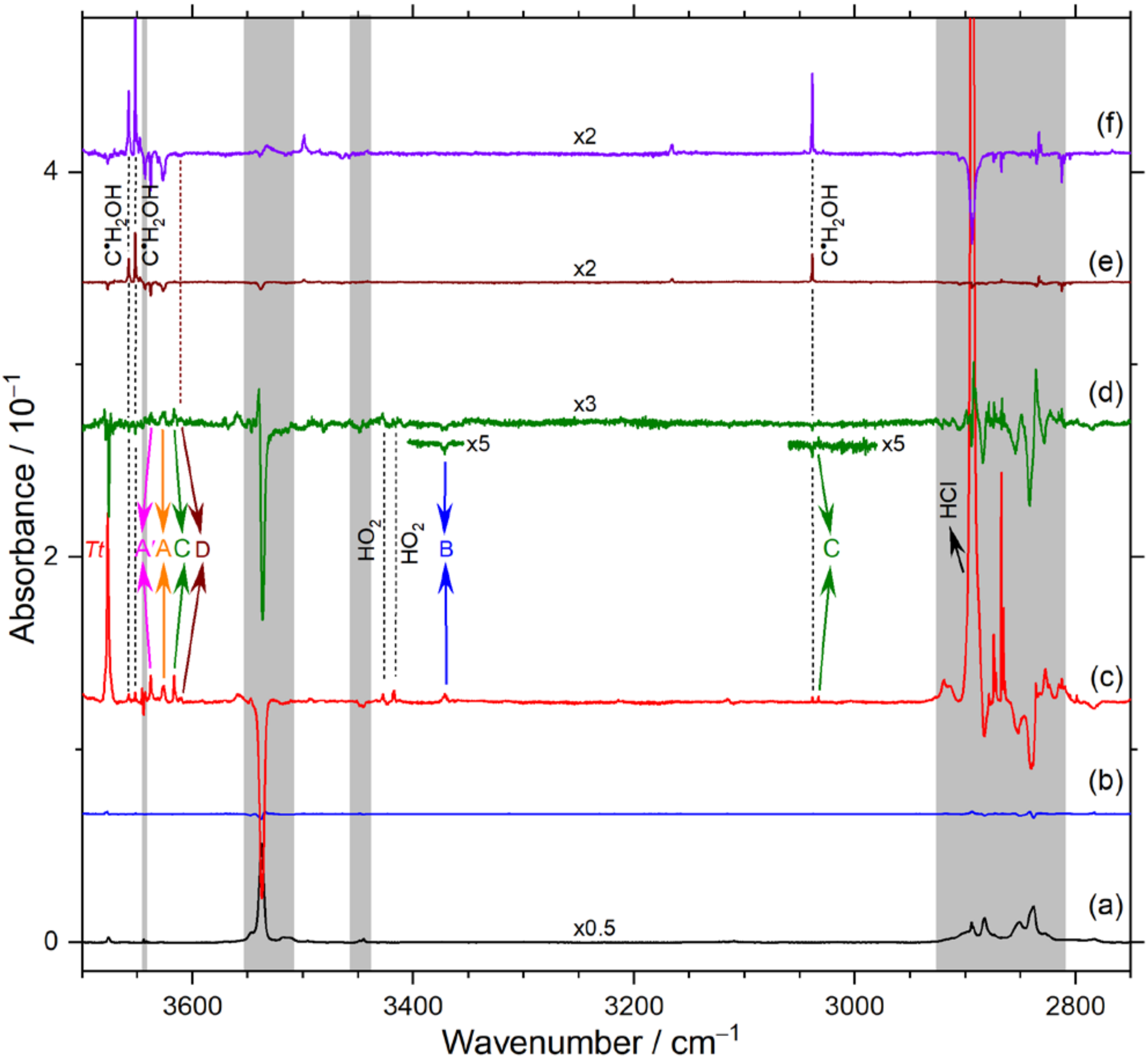

Absorbance / 10^{-1}
Wavenumber / cm^{-1}
(f)
(e)
(d)
(c)
(b)
(a)
x2
x2
x3
x5
x5
x0.5
C•H2OH
C•H2OH
C•H2OH
HO2
HO2
HCl
Tt
A'
A
C
D
B
C

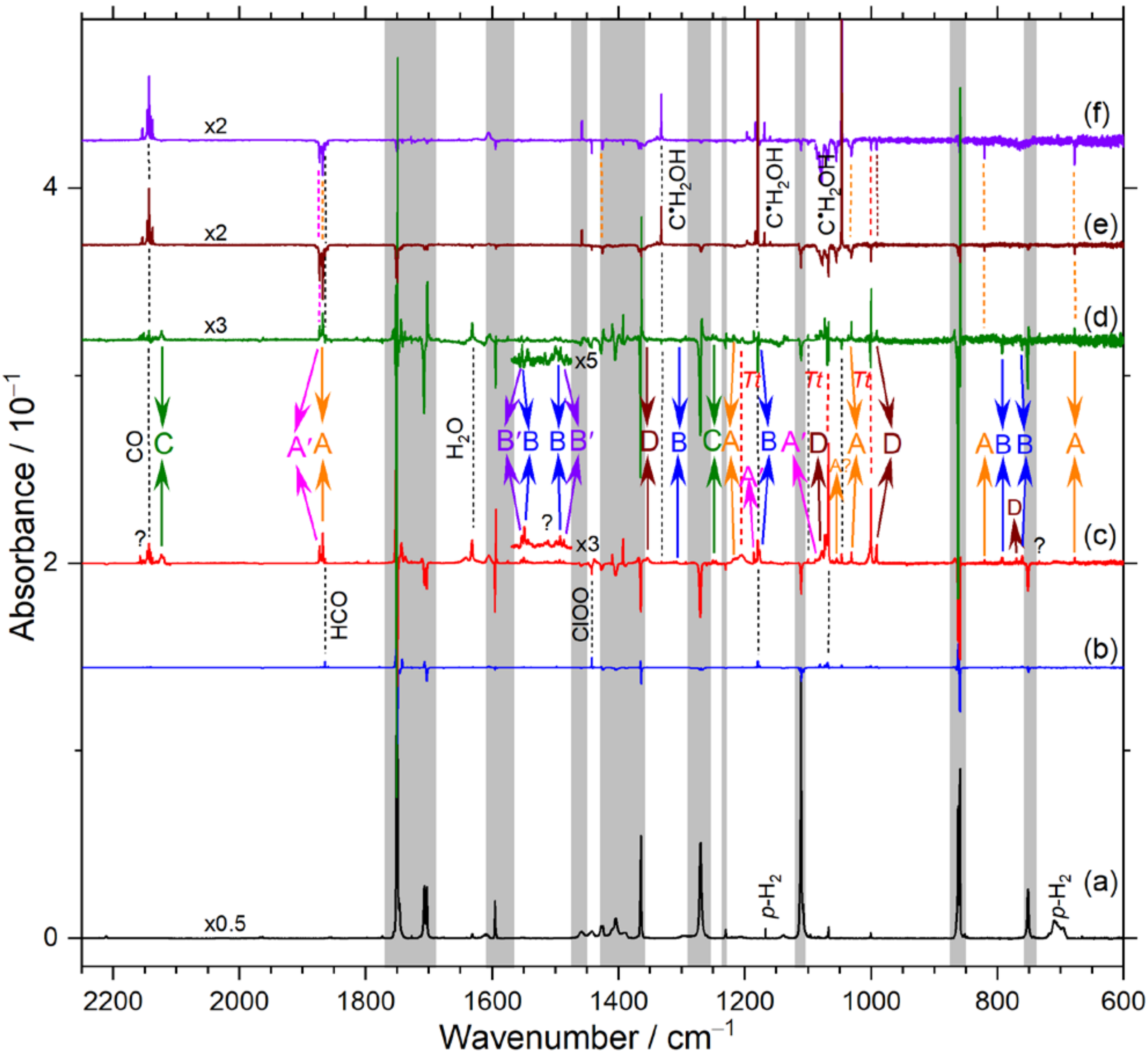

Figure S2. Full-range spectra (except 2750–2250 cm^{-1}) of a GA/Cl_2/p-H_2 (1/10/10000) matrix recorded at various stages of the H + *Cc*-GA experiment. (a) Spectrum recorded after deposition at 3.2 K for 6 h. (b) Difference spectrum after photolysis at 380 nm for 30 min. (c) Difference spectrum after additional full IR irradiation for 1 h. (d) Difference spectrum after terminating the IR irradiation and maintaining the matrix in darkness for 10 h. (e) Difference spectrum after secondary photolysis at 520 nm for 15 min. (f) Difference spectrum after secondary photolysis at 460 nm for 15 min. Lines in groups A–D, A′, and B′ are marked with orange, blue, green, brown, pink, and violet arrows and labels, these lines are later assigned as *Cc*-$HOCH_2C^{•}O$ **(3),** *Cc*-$HOC^{•}HC(O)H$ **(4),** HOCHCO **(6)**, *Cc*-$HOCH_2CH_2O^{•}$ **(1),** *Tt*-$HOCH_2C^{•}O$ **(3′)**, and *Cc*-$HOC^{•}HC(O)H$ **(4′)** respectively; lines of *Tt*-GA are indicated with red dashed lines and labelled *Tt*. The shaded gray areas indicate spectral regions subject to interference by the absorption of *Cc*-GA. Unidentified lines at 2157.2, 1512.6, and 732.1 cm^{-1} are denoted by question marks.

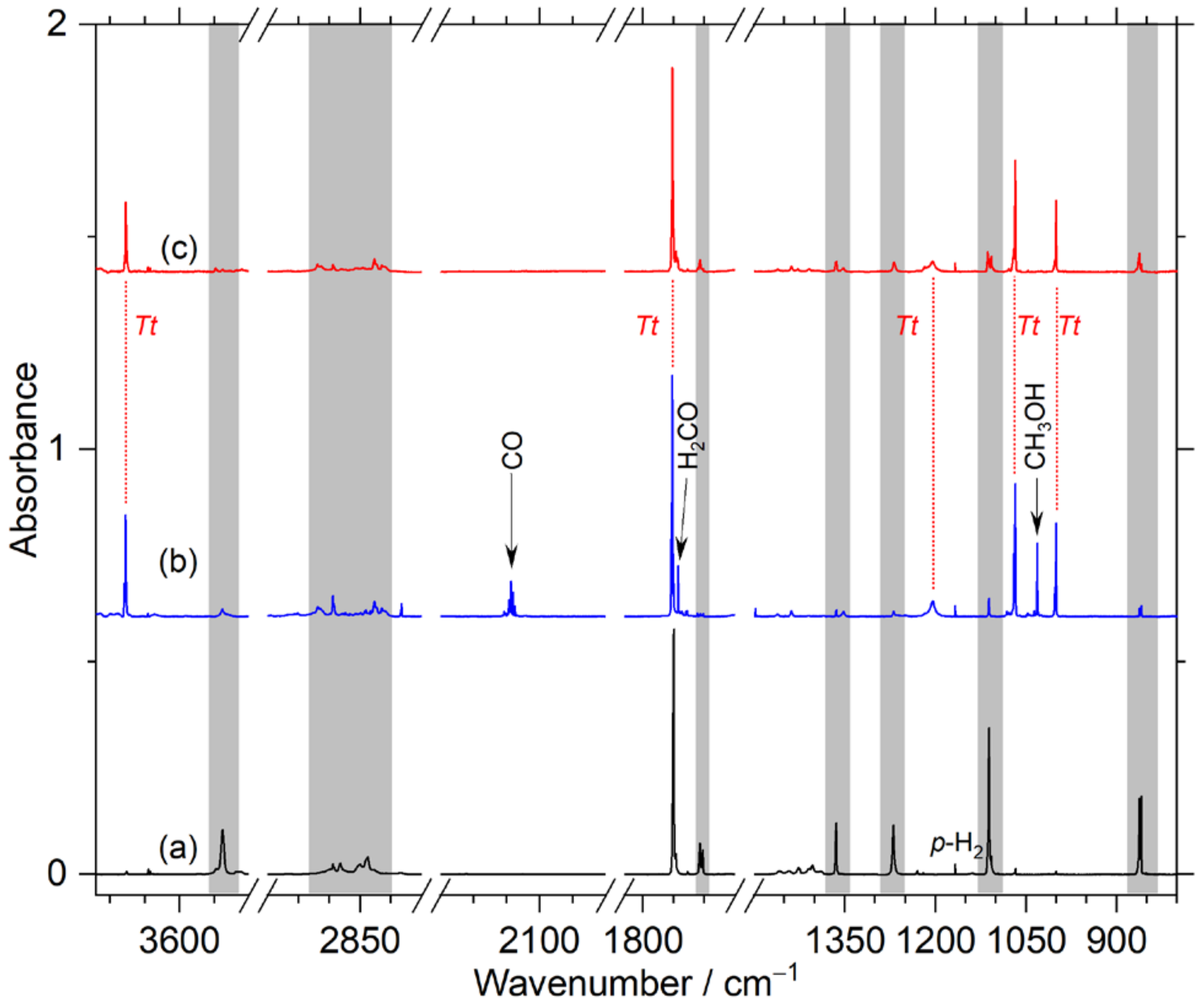

Figure S3. Representative spectra of a GA/Cl_2/*p*-H_2 (1/10/10000) matrix after irradiations at 266 nm or 2827 nm. (a) Spectrum recorded after deposition at 3.2 K for 6 h. (b) Spectrum recorded after irradiation of the matrix at 266 nm for 6 h. (c) Spectrum recorded after irradiation of the matrix at 2827 nm (ν_{OH} =3538 cm^{-1} of *Cc*-GA) for 15 h. Lines of *Tt*-GA are indicated with red dashed lines and labelled *Tt*.

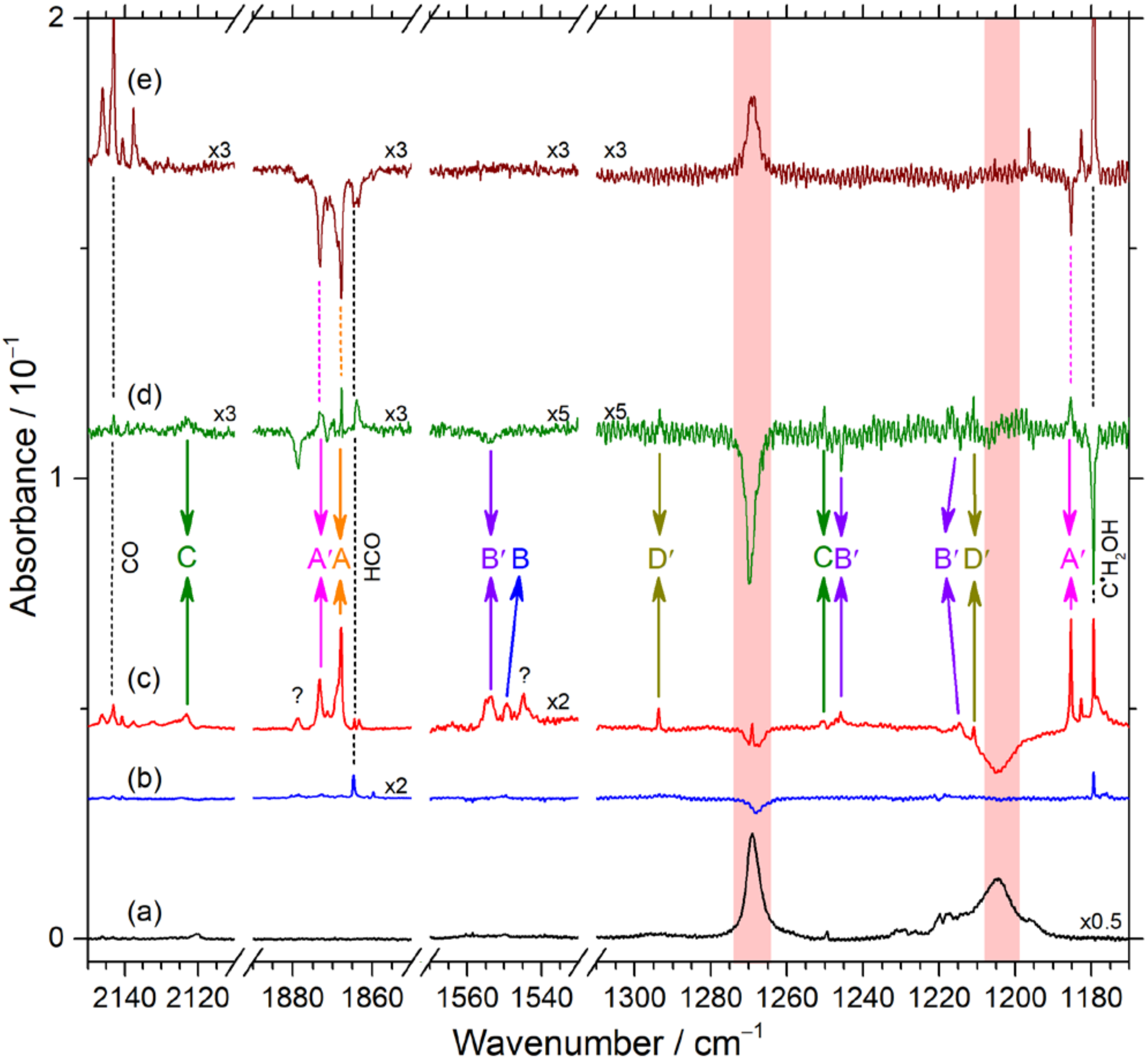

Figure S4. Representative spectra of a GA/Cl_2/*p*-H_2 (1/10/10000) matrix recorded at various stages of the H + *Tt*-GA experiment. (a) Spectrum recorded after irradiation of the matrix at 2827 nm for 15 h following the deposition at 3.2 K. Difference spectrum after photolysis at 380 nm for 30 min (b), after additional full IR irradiation for 1 h (c), after terminating the IR irradiation and maintaining the matrix in darkness for 10 h (d), and after secondary photolysis at 520 nm for 15 min (e). Lines in groups A′, B′, C, D′, A, and B are marked with pink, violet, green, olive, orange, and blue arrows and labels; these lines are assigned as *Tt*-$HOCH_2C^{\bullet}O$ **(3′)**, *Tt*-$HOC^{\bullet}HC(O)H$ **(4′)**, HOCHCO **(6)**, *Tt*-$HOCH_2CH_2O^{\bullet}$ **(1′)**, *Cc*-$HOCH_2C^{\bullet}O$ **(3)**, and *Cc*-$HOC^{\bullet}HC(O)H$ **(4)**, respectively. The shaded red areas indicate spectral regions subject to interference by the absorption of *Tt*-GA.

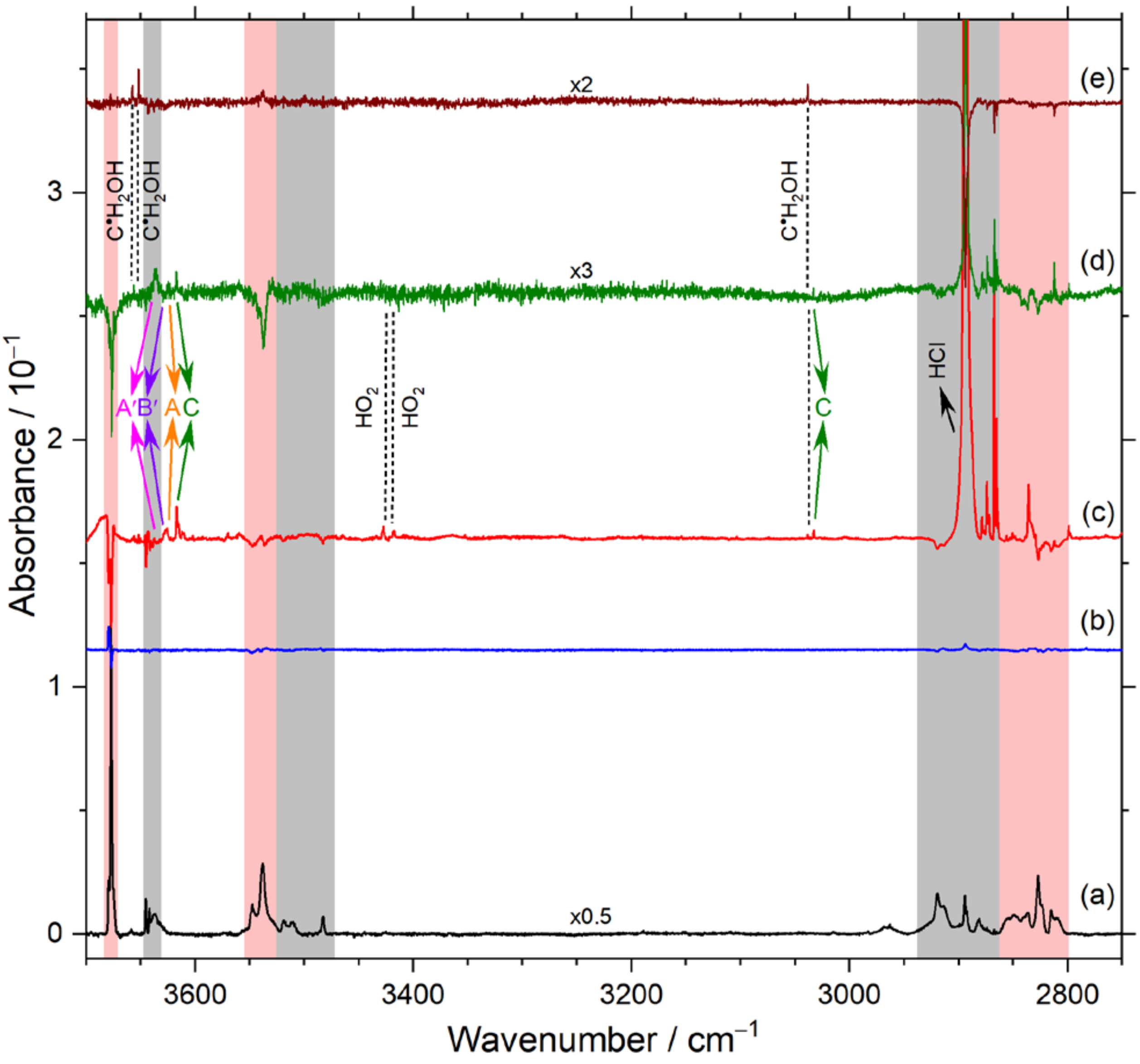

Absorbance / 10⁻¹
Wavenumber / cm⁻¹
(e)
(d)
(c)
(b)
(a)
x2
x3
x0.5
C•H2OH
C•H2OH
C•H2OH
A′
B′
A
C
C
HO2
HO2
HCl

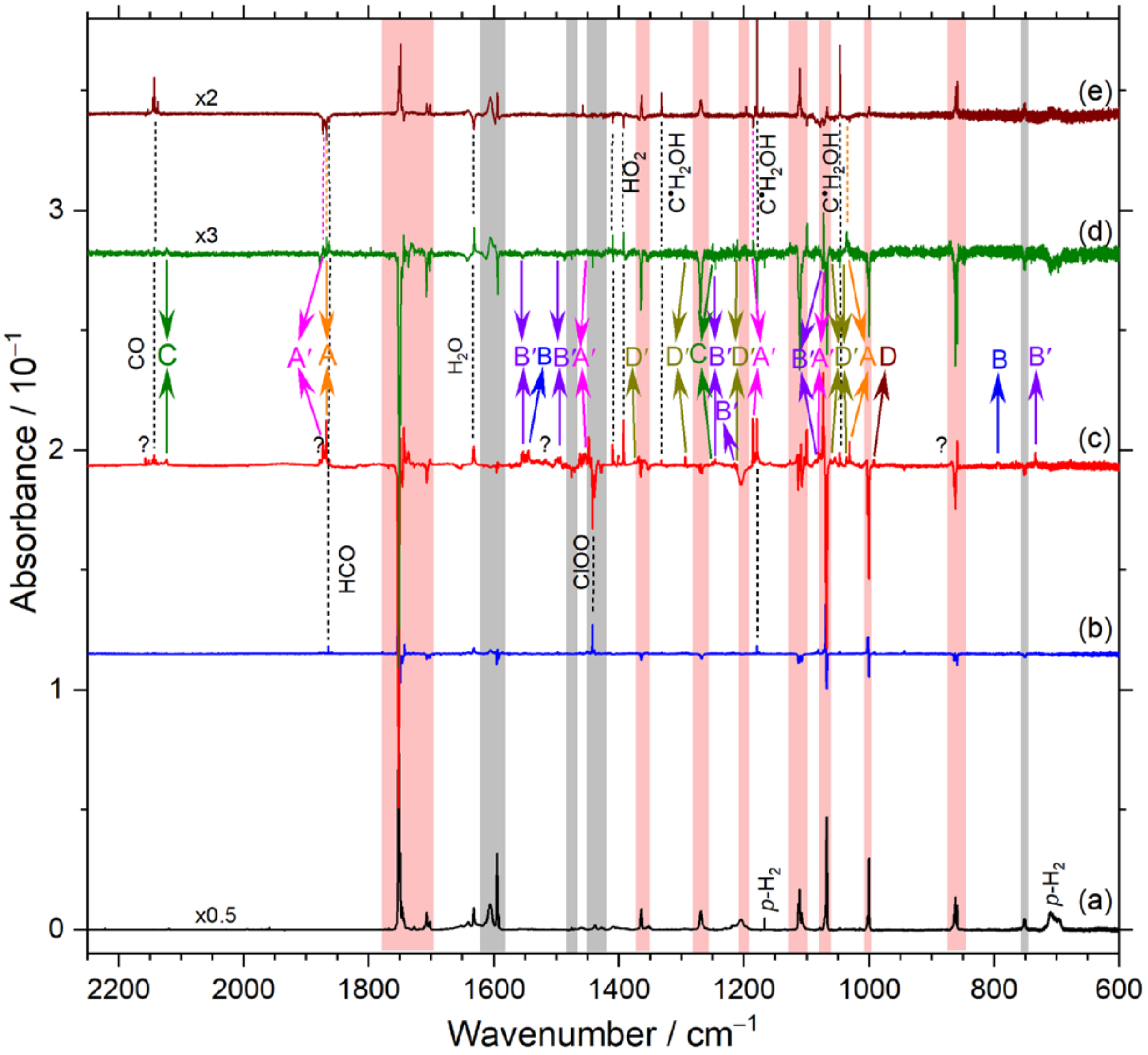

Figure S5. Full-range spectra (except 2750−2250 cm^{-1}) of a GA/Cl_2/*p*-H_2 (1/10/10000) matrix recorded at various stages of the H + *Tt*-GA experiment. (a) Spectrum recorded after irradiation of the matrix at 2827 nm (ν_{OH} =3538 cm^{-1} of *Cc*-GA) for 15 h following the deposition at 3.2 K. (b) Difference spectrum after photolysis at 380 nm for 30 min. (c) Difference spectrum after additional full IR irradiation for 1 h. (d) Difference spectrum after terminating the IR irradiation and maintaining the matrix in darkness for 10 h. (e) Difference spectrum after secondary photolysis at 520 nm for 15 min. Lines in groups A−D, A′, B′, and D′ are marked with orange, blue, green, brown, pink, violet, and olive arrows and labels, these lines are later assigned as *Cc*-$HOCH_2C^•O$ **(3),** *Cc*-$HOC^•HC(O)H$ **(4),** HOCHCO **(6)**, *Cc*-$HOCH_2CH_2O^•$ **(1),** *Tt*-$HOCH_2C^•O$ **(3′)**, *Cc*-$HOC^•HC(O)H$ **(4′),** and *Tt*-$HOCH_2CH_2O^•$ **(1′)**, respectively. The shaded red and gray areas indicate spectral regions subject to interference by the absorption of *Cc*-GA and *Tt*-GA, respectively. Unidentified lines at 2157.2, 1877.9, 1512.6, and 732.1 cm^{-1} are denoted by question marks.

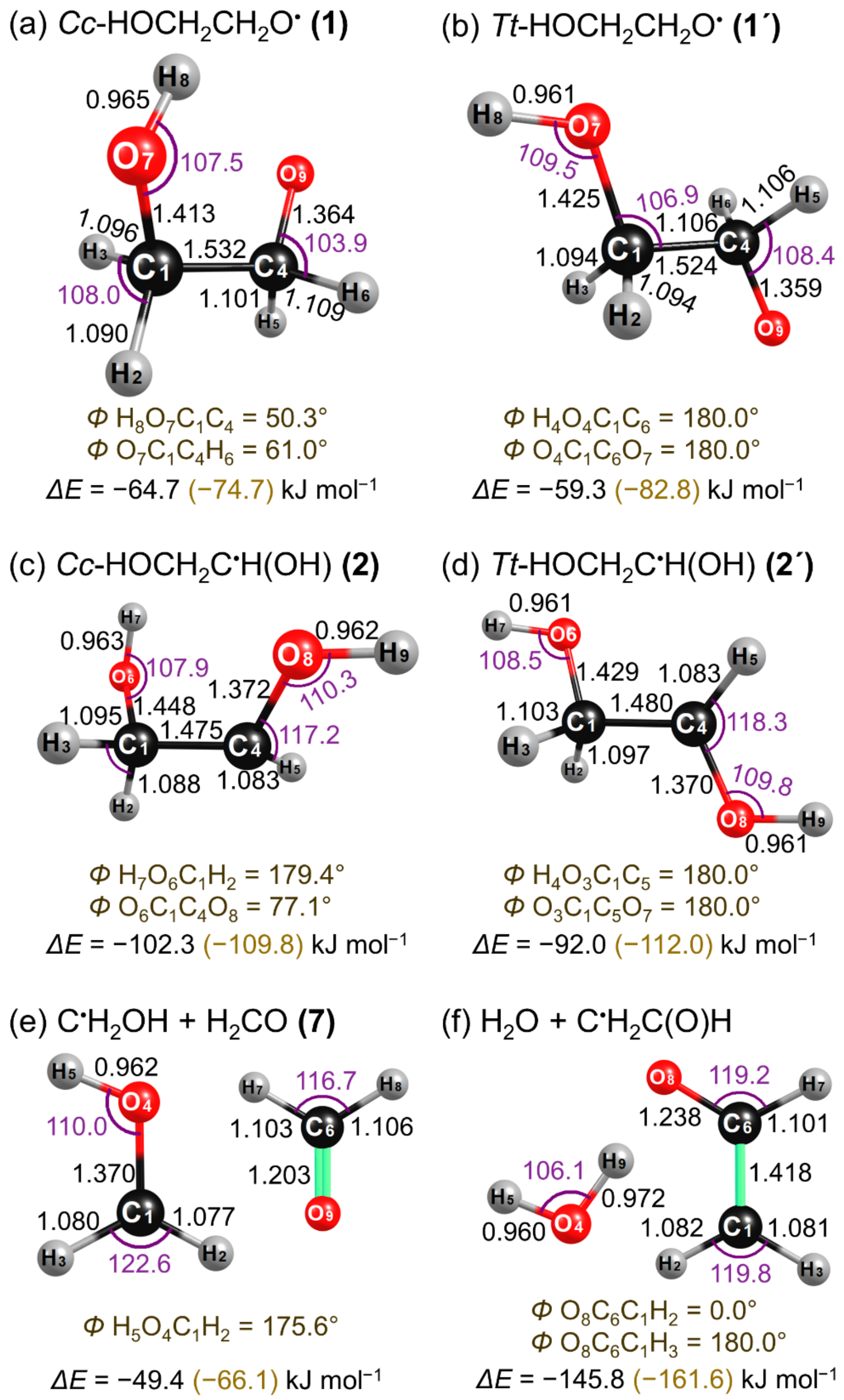

Figure S6. Geometries of H-addition and H-induced fragmentation products in reactions H + *Cc*-GA and H + *Tt*-GA optimized with the B3LYP/aug-cc-pVTZ method. (a) *Cc*-$HOCH_2CH_2O^\bullet$ **(1)**, (b) *Tt*-$HOCH_2CH_2O^\bullet$ **(1′)**, (c) *Cc*-$HOCH_2C^\bullet H(OH)$ **(2)**, (d) *Tt*- $HOCH_2C^\bullet H(OH)$ **(2′)**, (e) $C^\bullet H_2OH + H_2CO$ **(7)**, and (f) $H_2O + C^\bullet H_2C(O)H$. Bond lengths (black) in angstrom (Å) and bond angles (purple) and dihedral angles Φ are in degrees (°). Relative energies (in kJ mol^{-1}) calculated with the CCSD(T)/aug-cc-pVTZ//B3LYP/aug-cc-pVTZ method (black) are listed; those with the B3LYP/aug-cc-pVTZ method (olive) are listed in parentheses for comparison.

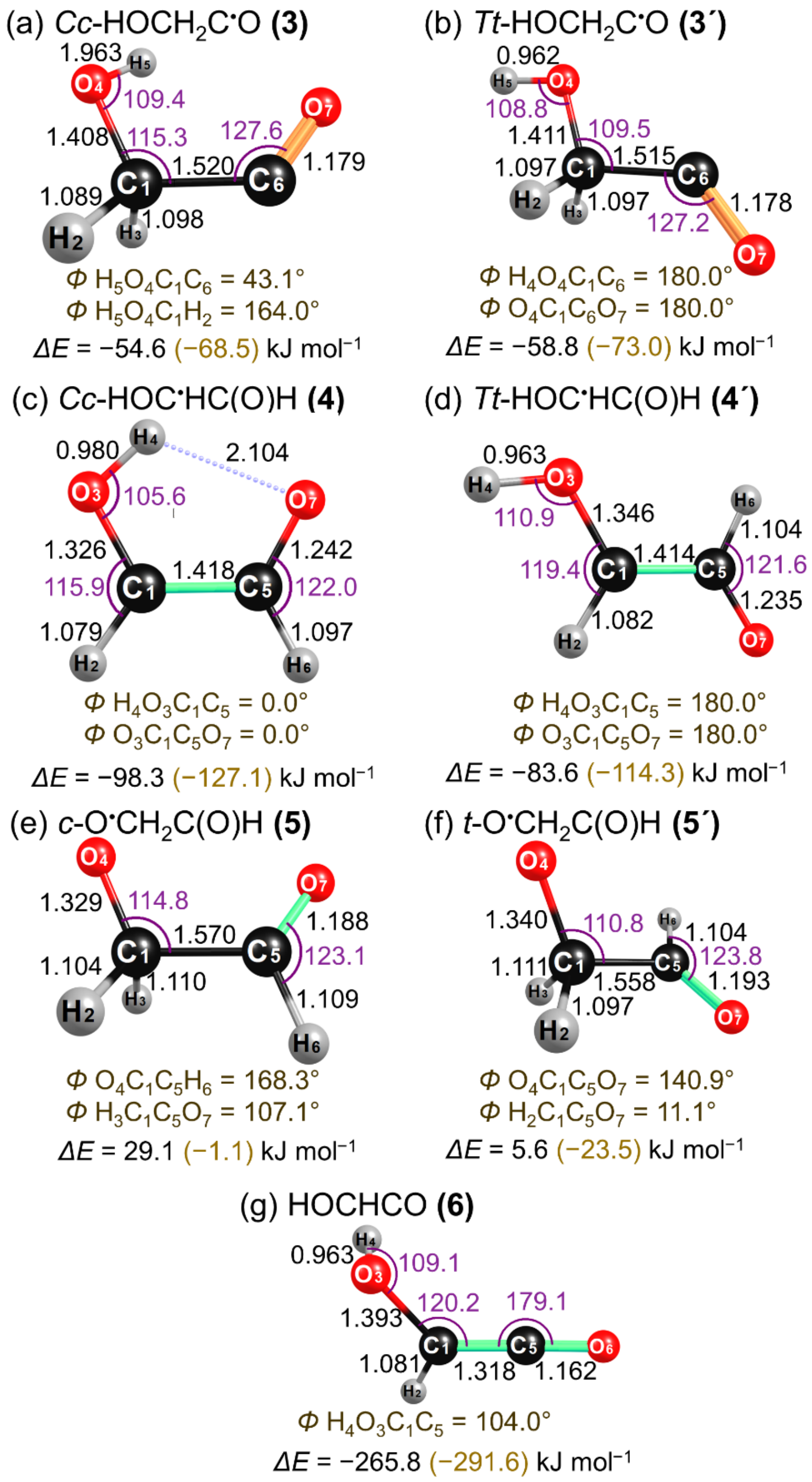

Figure S7. Geometries of H-abstraction products in reactions H + *Cc*-GA and H + *Tt*-GA optimized with the B3LYP/aug-cc-pVTZ method. (a) *Cc*-$HOCH_2C^•O$ **(3)**, (b) *Tt*-$HOCH_2C^•O$ **(3′)**, (c) *Cc*-$HOC^•HC(O)H$ **(4)**, (d) *Tt*-$HOC^•HC(O)H$ **(4′)**, (e) *c*-$O^•CH_2C(O)H$ **(5)**, (f) *t*-$O^•CH_2C(O)H$ **(5′)**, and (g) HOCHCO **(6)**. Bond lengths (black) in angstrom (Å) and bond angles (purple) and dihedral angles Φ are in degrees (°). Energies (in kJ mol^{-1}) relative to H + *Cc*-GA (or H + *Tt*-GA for **3′**, **4′**, and **5′**) calculated with the CCSD(T)/aug-cc-pVTZ//B3LYP/aug-cc-pVTZ method (black) are listed; those with the B3LYP/aug-cc-pVTZ method (olive) are listed in parentheses for comparison.

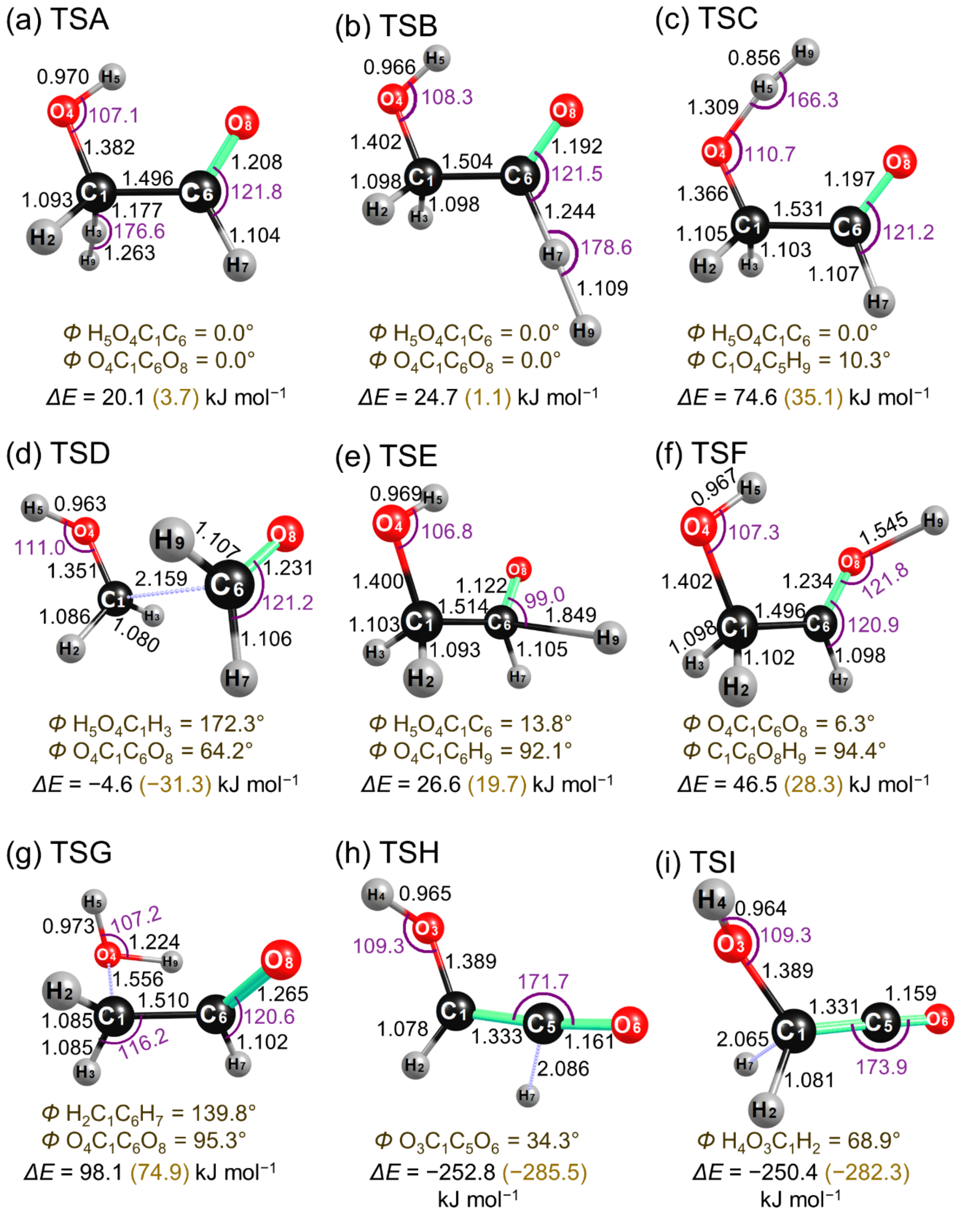

Figure S8. Geometries of transition states in the reaction H + *Cc*-GA optimized with the B3LYP/aug-cc-pVTZ method. (a) TSA, (b) TSB, (c) TSC, (d) TSD, (e) TSE, (f) TSF, (g) TSG, (h) TSH, and (i) TSI. Bond lengths (black) in angstrom (Å) and bond angles (purple) and dihedral angles Φ are in degrees (°). Energies (in kJ mol^{-1}) relative to H + *Cc*-GA calculated with the CCSD(T)/aug-cc-pVTZ//B3LYP/aug-cc-pVTZ method (black) are listed; those with the B3LYP/aug-cc-pVTZ method (olive) are listed in parentheses for comparison.

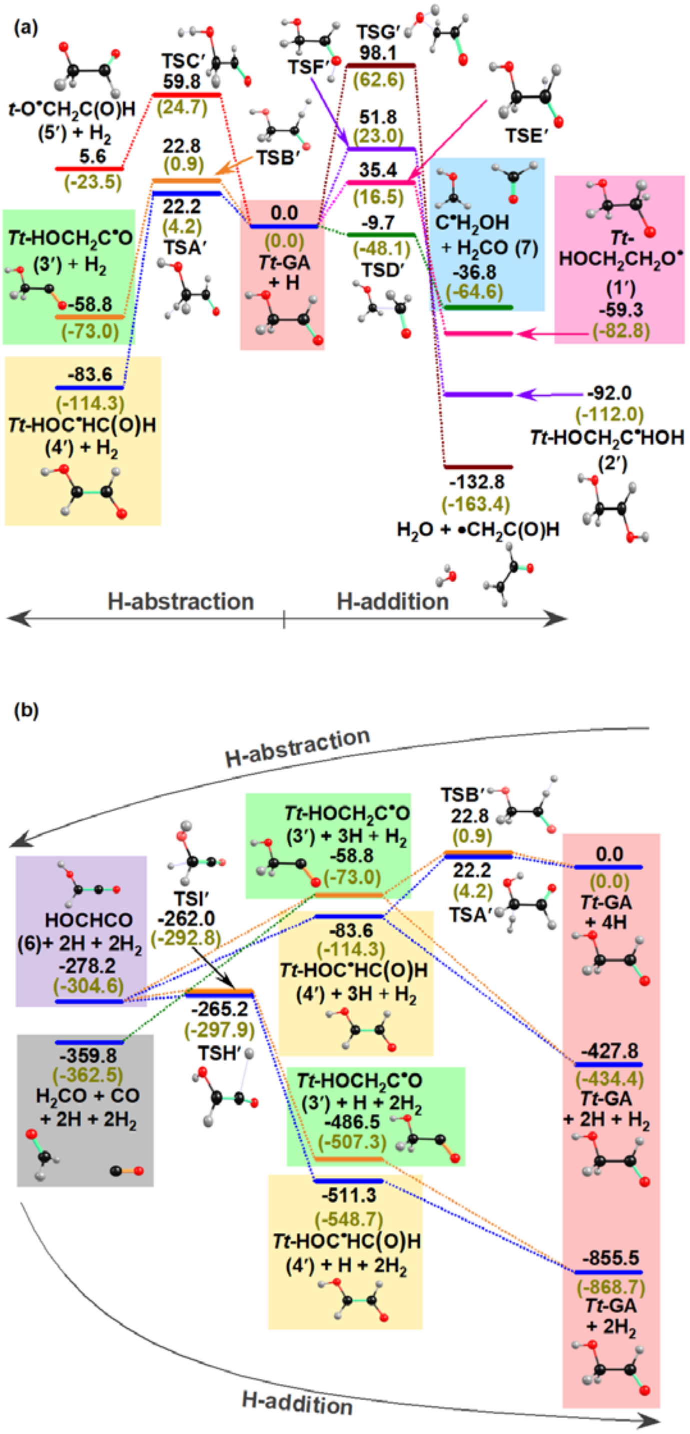

Figure S9. Potential-energy scheme of various channels predicted for the reaction H + *Tt*-$HOCH_2C(O)H$ (*Tt*-GA). (a) All possible H-abstraction and H-addition channels of H + *Tt*-GA; energies are relative to H + *Tt*-GA. (b) Two most feasible successive H-abstraction channels followed by two H-addition channels connecting *Tt*-GA with HOCHCO **(6)** via *Tt*- HOC•HC(O)H **(4′)** or *Tt*-$HOCH_2C^{•}O$ **(3′)** and possible formation of fragmented products H_2CO + CO; energies are relative to 4H + *Tt*-GA. Energies (in kJ mol^{-1}) were calculated with the CCSD(T)/aug-cc-pVTZ//B3LYP/aug-cc-pVTZ method (black); values in parentheses (olive) were calculated with the B3LYP/aug-cc-pVTZ method. Zero-point vibrational energies (ZPVE), calculated with the B3LYP/ aug-cc-pVTZ method, were corrected.

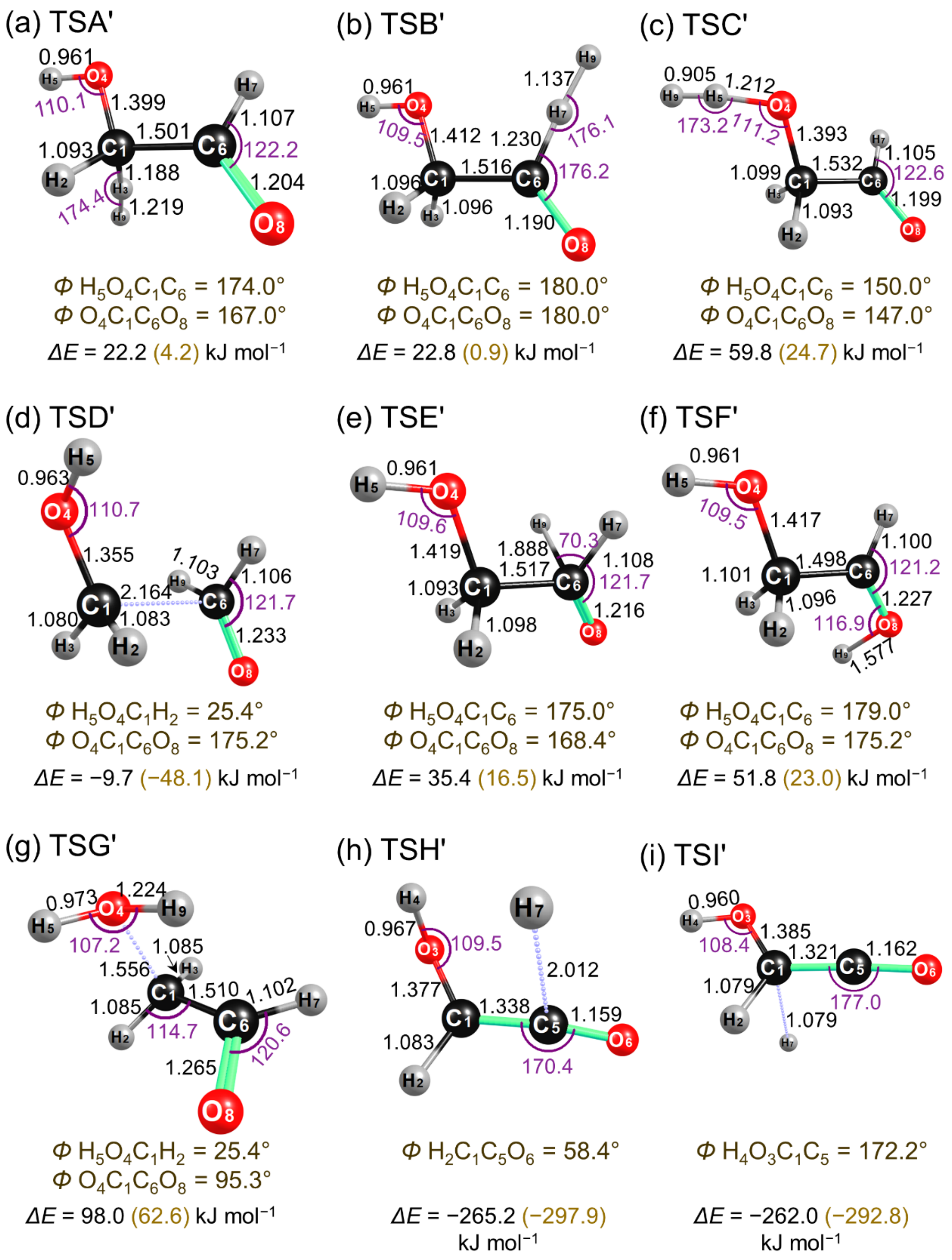

Figure S10. Geometries of transition states in the reaction H + *Tt*-GA optimized with the B3LYP/aug-cc-pVTZ method. (a) TSA′, (b) TSB′, (c) TSC′, (d) TSD′, (e) TSE′, (f) TSF′, (g) TSG′, (h) TSH′, and (i) TSI'. Bond lengths (black) in angstrom (Å) and bond angles (purple) and dihedral angles Φ are in degrees (°). Energies (in kJ mol^{-1}) relative to H + *Cc*-GA calculated with the CCSD(T)/aug-cc-pVTZ//B3LYP/aug-cc-pVTZ method (black) are listed; those with the B3LYP/aug-cc-pVTZ method (olive) are listed in parentheses for comparison.

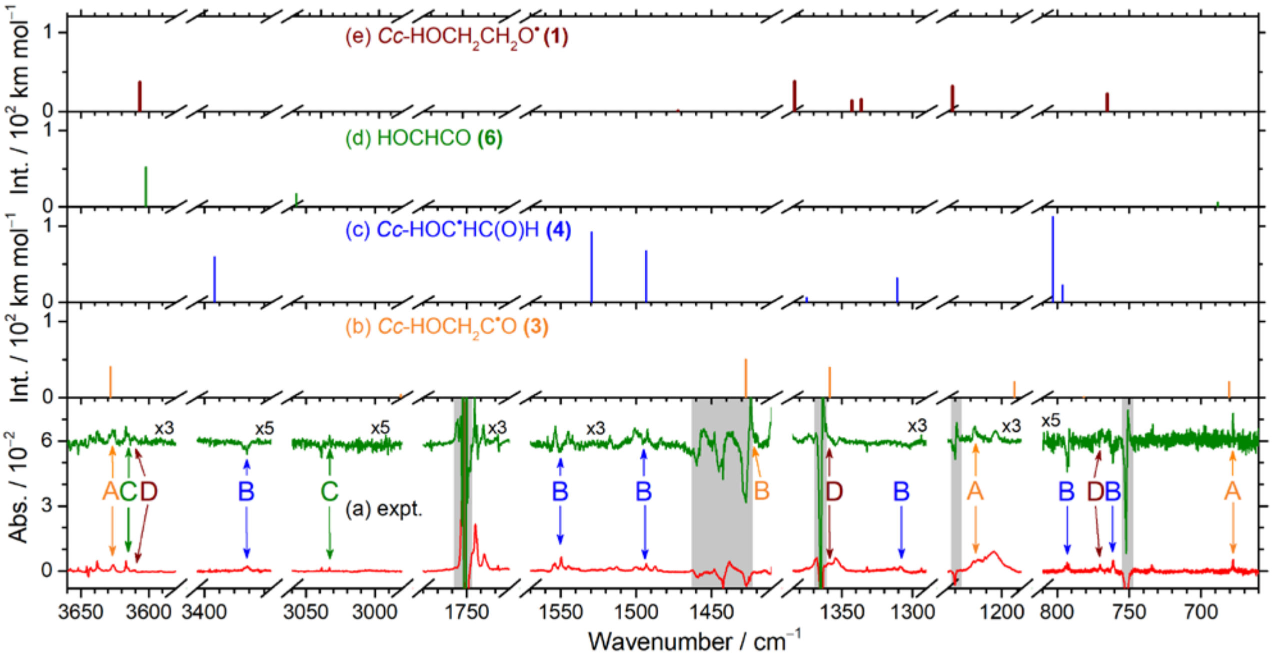

Figure S11. Comparison of lines in groups A−D with predicted IR stick spectra of possible H-reaction products of H + *Cc*-GA. (a) The lower trace is from Figure S2c and the upper trace from Figure S2d; lines in groups A−D are marked with orange, blue, green, and brown arrows and labels. The predicted spectra are (b) *Cc*-HOCH$_2$C$^•$O **(3)**, (c) *Cc*-HOC$^•$HC(O)H **(4)**, (d) HOCHCO **(6)**, and (e) *Cc*-HOCH$_2$CH$_2$O$^•$ **(1)**, according to scaled harmonic vibrational wavenumbers. The regions severely interfered with by absorptions of *Cc*-GA are in gray.

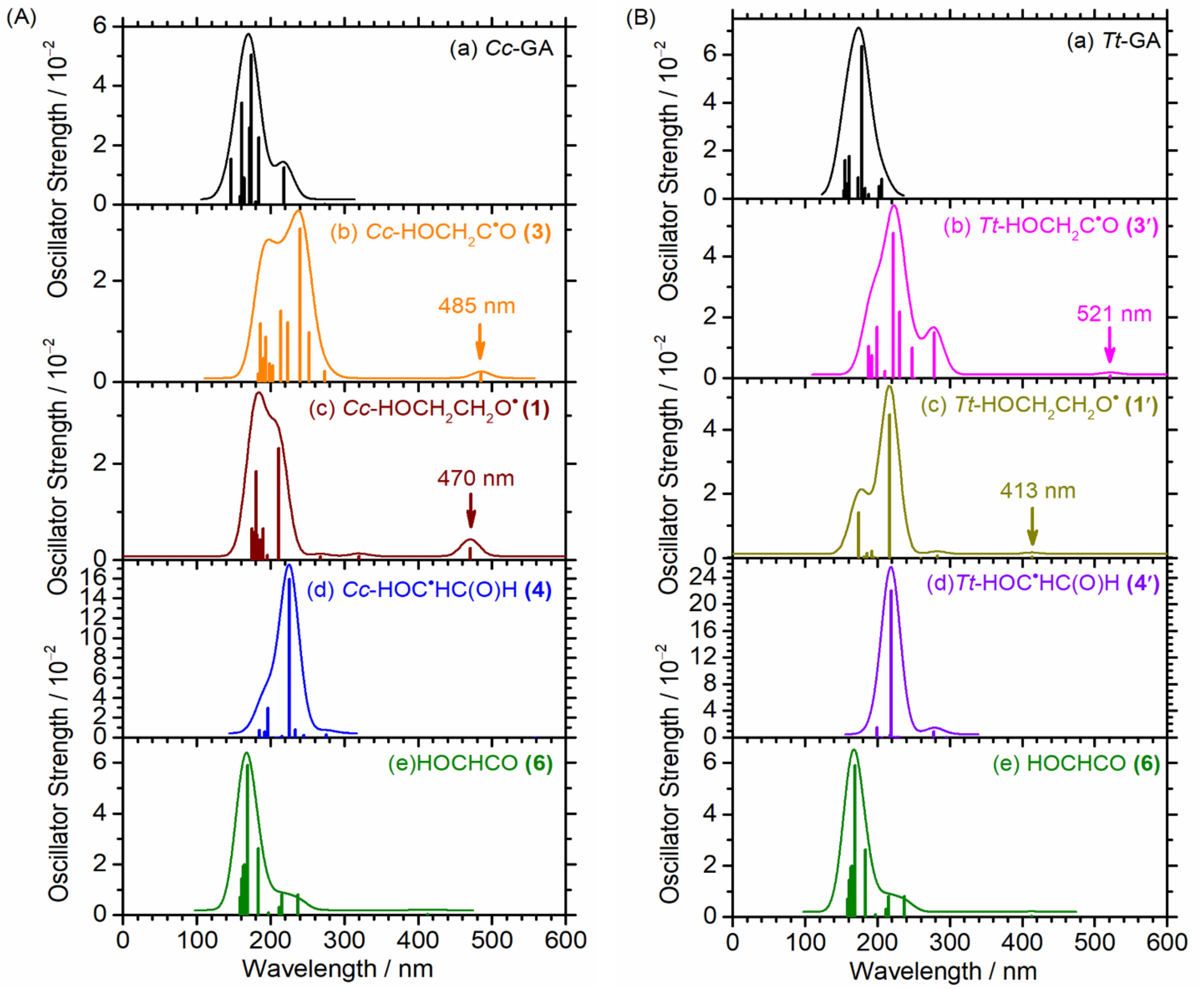

Figure S12. Vertical excitation UV spectra of various products formed in the reactions of H + *Cc*-GA (A) and H + *Tt*-GA (B). Left panel: (a) *Cc*-GA, (b) *Cc*-$HOCH_2C^{\bullet}O$ **(3)**, (c) *Cc*-$HOCH_2CH_2O^{\bullet}$ **(1)**, (d) *Cc*-$HOC^{\bullet}HC(O)H$ **(4)**, and (e) HOCHCO **(6)**. Right panel: (a) *Tt*-GA, (b) *Tt*-$HOCH_2C^{\bullet}O$ **(3′)**, (c) *Tt*-$HOCH_2CH_2O^{\bullet}$ **(1′)**, (d) *Tt*-$HOC^{\bullet}HC(O)H$ **(4′)**, and (e) HOCHCO **(6)**. The stick spectra were calculated with the TD-B3LYP/aug-cc-pVTZ method and the traces were convoluted with a full-width-half-maximum (FWHM) of 30 nm.

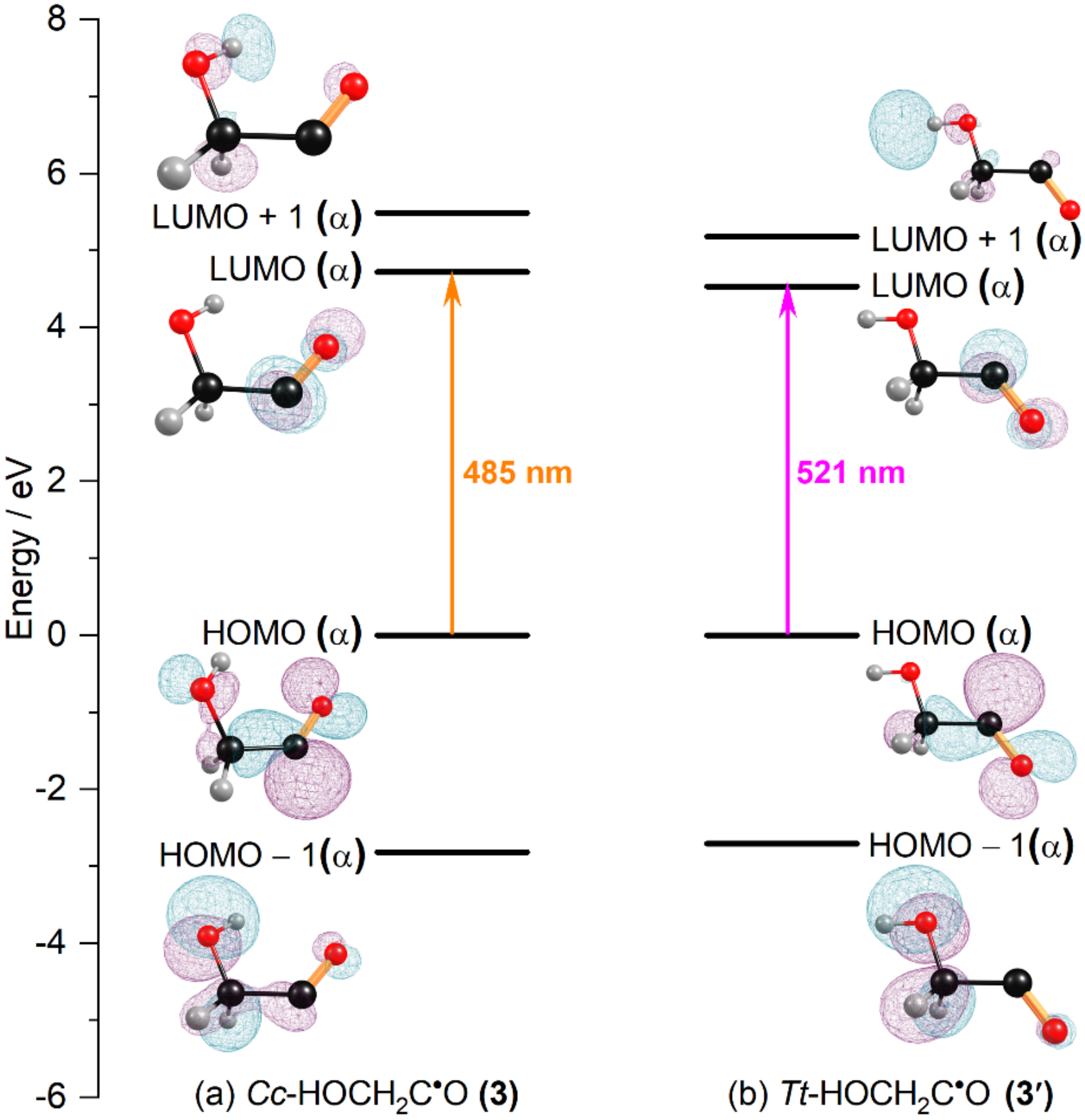

Figure S13. The frontier molecular orbital diagrams of *Cc*-$HOCH_2C^{\bullet}O$ **(3)** and *Tt*-$HOCH_2C^{\bullet}O$ **(3′)**. The isovalue contours (0.03 a.u.) were calculated with the TD-B3LYP/aug-cc-pVTZ method. The wavelengths of the first electronic transitions of each species are indicated.

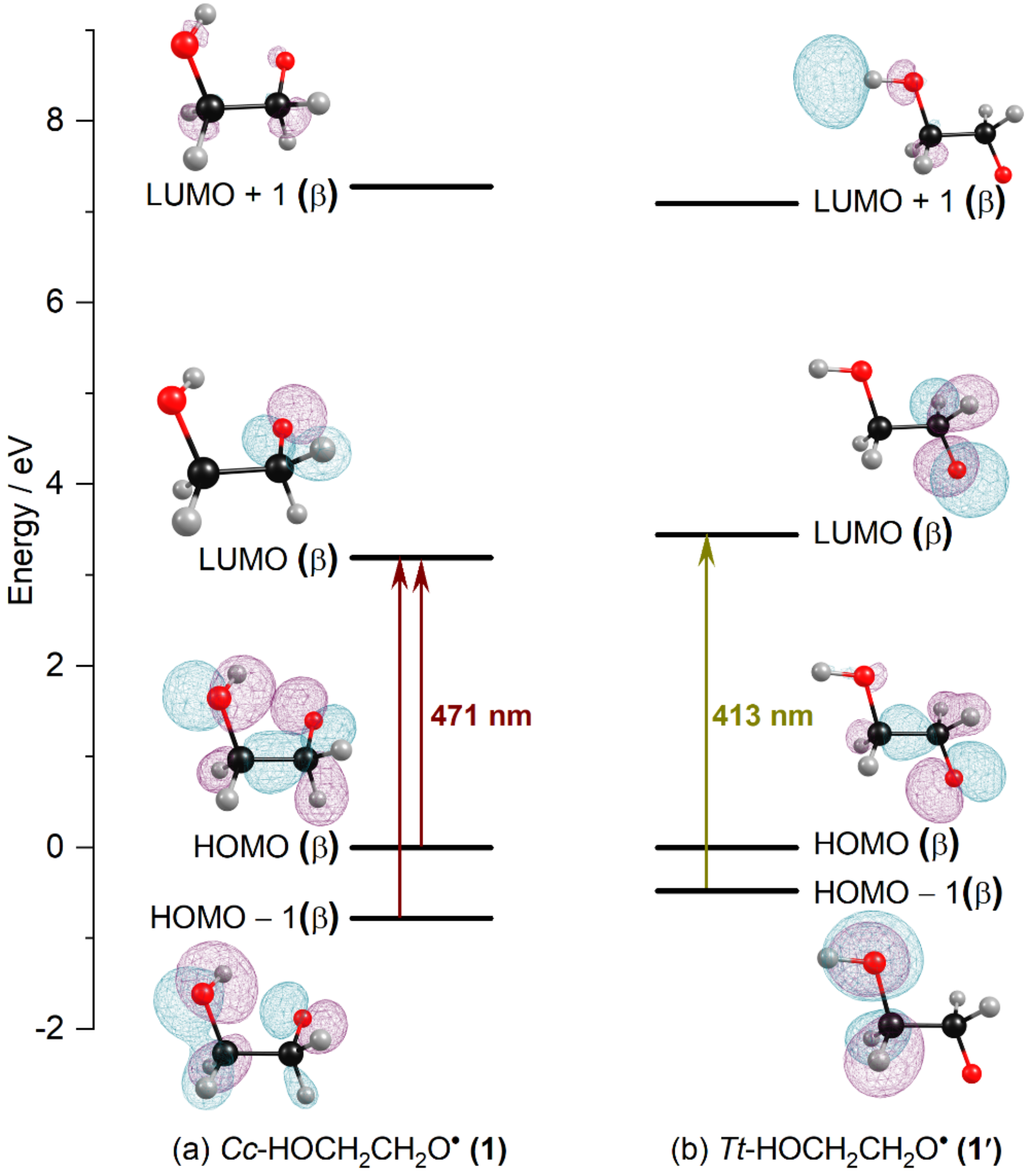

Figure S14. The frontier molecular orbital diagrams of *Cc*-$HOCH_2CH_2O^•$ **(1)** and *Tt*- $HOCH_2CH_2O^•$ **(1′)**. The isovalue contours (0.03 a.u.) were calculated with the TD-B3LYP/aug-cc-pVTZ method. The wavelengths of the first electronic transitions of each species are indicated.

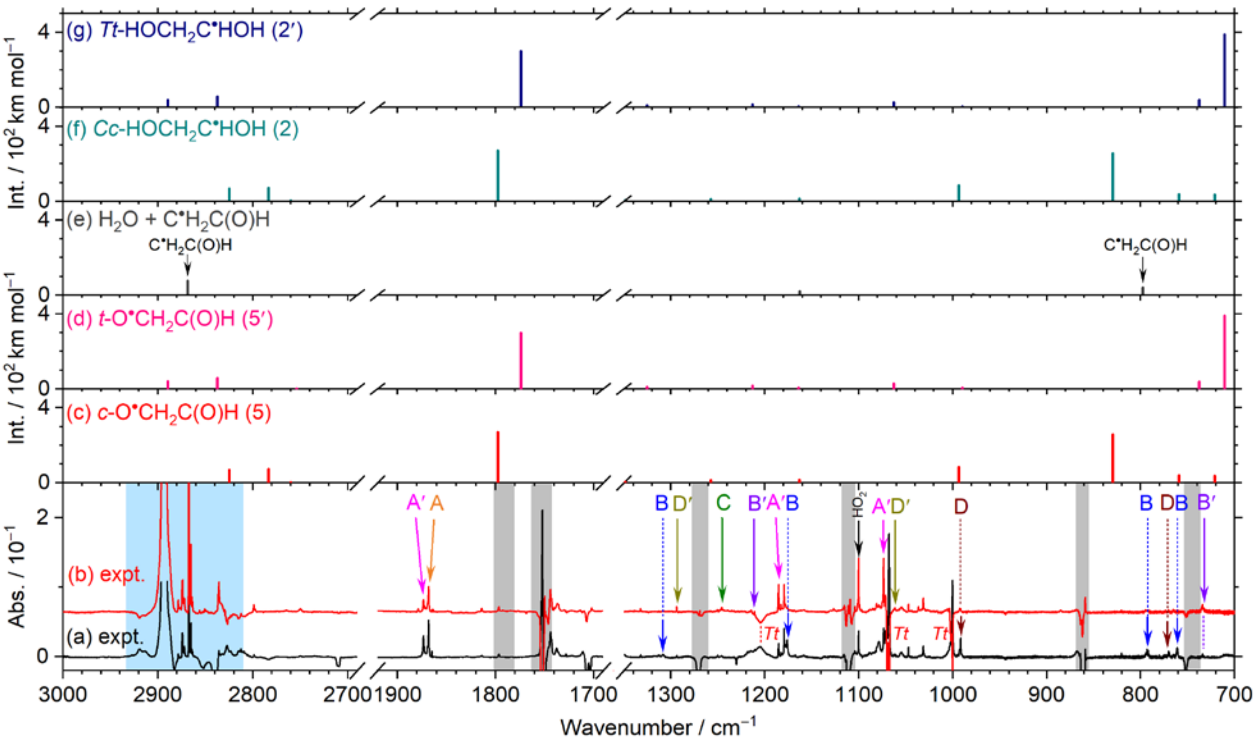

Figure S15. Comparison of lines in groups A−D, A′, B′, and D′ with predicted IR stick spectra of other H-reaction products of H + *Cc*-GA and H + *Tt*-GA. (a) The spectra are from Figures 2c and S2c and (b) the spectra are from Figure 3c and S4c; lines in groups A−D, A′, B′, and D′ are marked with orange, blue, green, brown, pink, violet, and olive arrows and labels. The predicted stick spectra are (c) *c*-O•$CH_2C(O)H$ **(5)**, (d) *t*-O•$CH_2C(O)H$ **(5′)**, (e) H_2O + C•$H_2C(O)H$, (f) *Cc*-$HOCH_2C$•HOH **(2)**, and (g) *Tt*-$HOCH_2C$•HOH **(2′)**, according to scaled harmonic vibrational wavenumbers. The regions severely interfered with by absorptions of *Cc*-GA and HCl are in gray and blue, respectively.

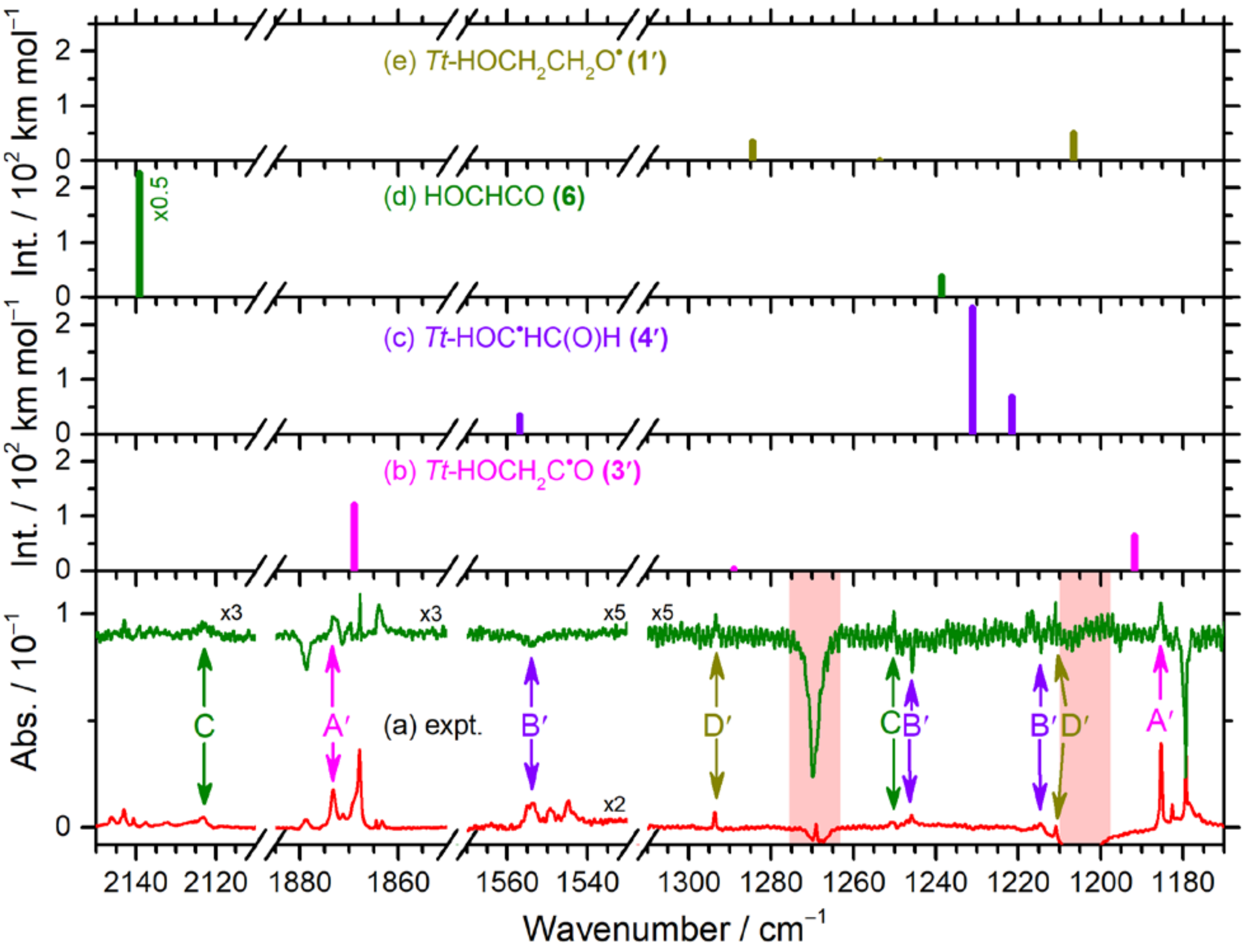

Figure S16. Comparison of lines in groups A′, B′, C, and D′ with predicted IR stick spectra of possible H-reaction products of possible H-reaction products of H + *Tt*-GA. (a) The lower trace is from Figure S4c and the upper trace is from Figure S4d; lines in groups A′, B′, C, and D′ are marked with pink, violet, green, and olive arrows and labels. The predicted stick spectra are (b) *Tt*-$HOCH_2C^{\bullet}O$ **(3′)**, (c) *Tt*-$HOC^{\bullet}HC(O)H$ **(4′)**, (d) HOCHCO **(6)**, and (e) *Tt*-$HOCH_2CH_2O^{\bullet}$ **(1′)**, according to scaled harmonic vibrational wavenumbers. The regions severely interfered with by absorptions of *Tt*-GA are in red.

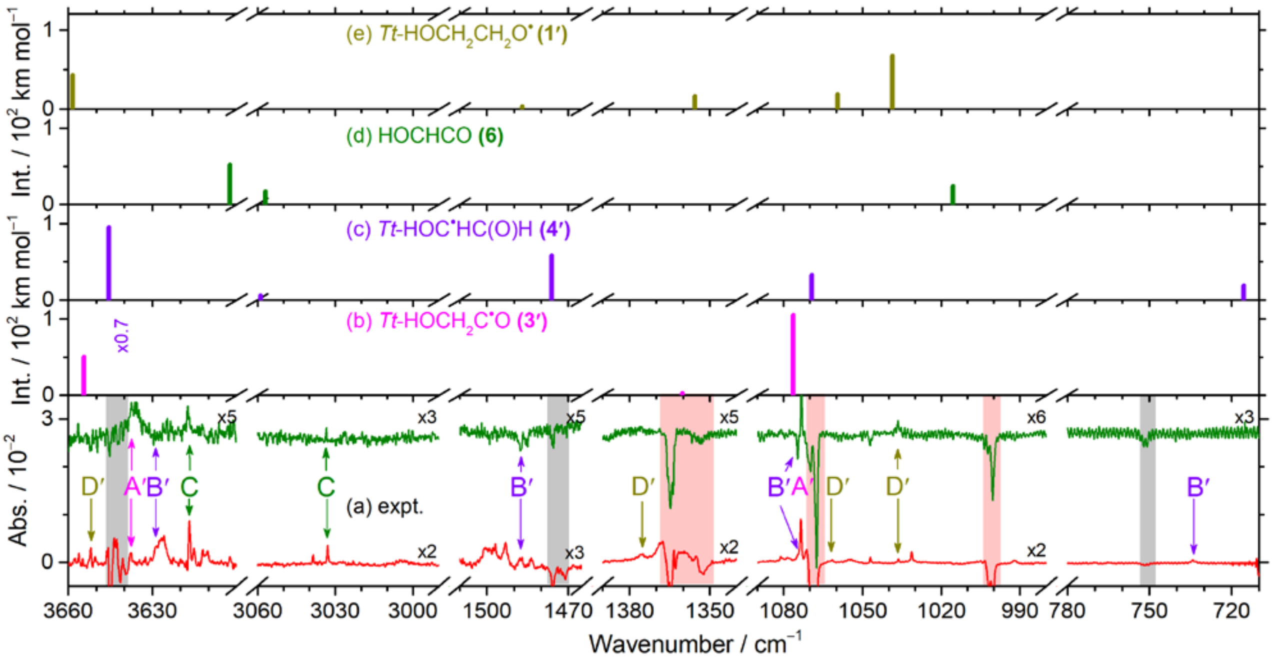

Figure S17. Comparison of lines in groups A′, B′, C, and D′ with predicted IR stick spectra of possible H-reaction products of H + *Tt*-GA in different spectral regions. (a) The lower trace is from Figure S4c and the upper trace is from Figure S4d; lines in groups A′, B′, C, and D′ are marked with pink, violet, green, and olive arrows and labels. The predicted stick spectra are (b) *Tt*-$HOCH_2C^{\bullet}O$ **(3′)**, (c) *Tt*-$HOC^{\bullet}HC(O)H$ **(4′)**, (d) HOCHCO **(6)**, and (e) *Tt*-$HOCH_2CH_2O^{\bullet}$ **(1′)**, according to scaled harmonic vibrational wavenumbers. The regions severely interfered with by absorptions of Cc-GA and *Tt*-GA are in gray and red, respectively.

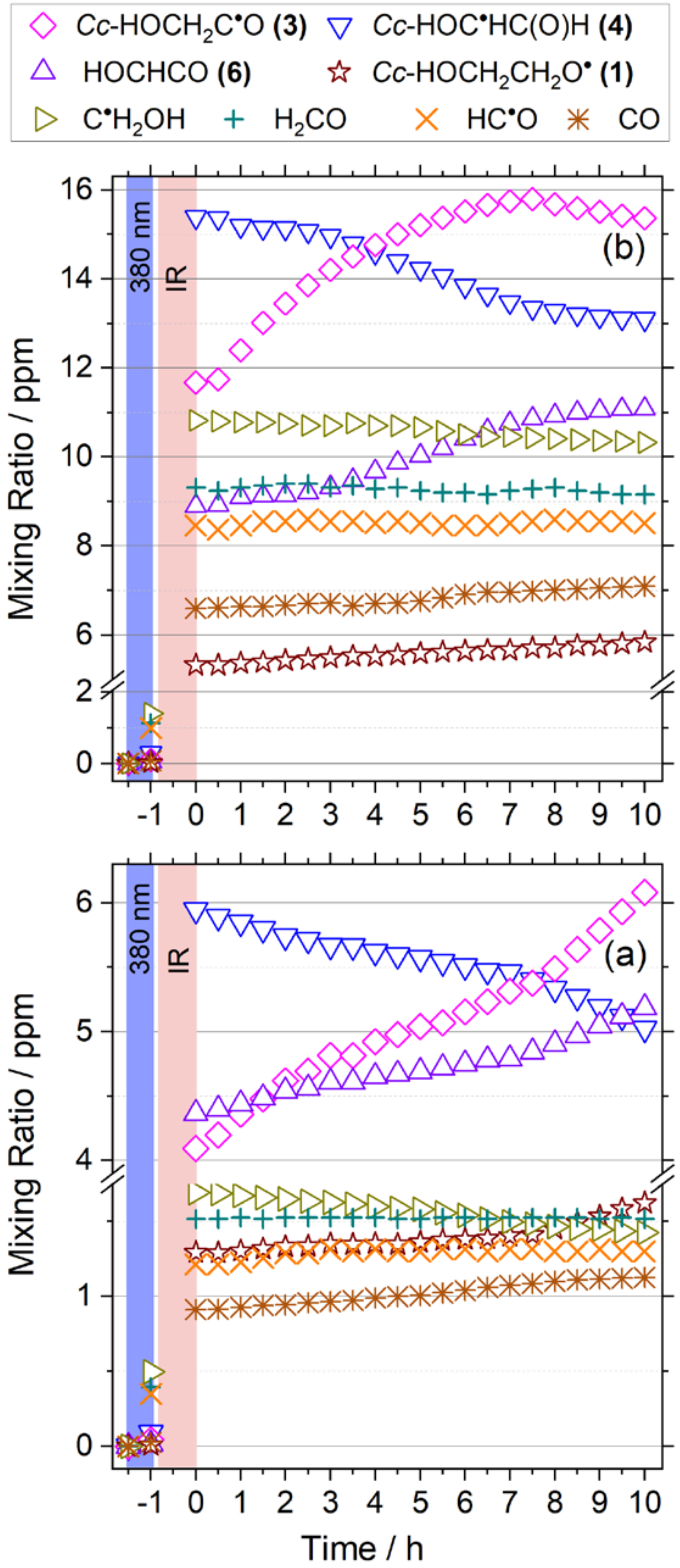

Figure 18. Expanded version of traces (a) and (b) of Figure 6: Temporal evolution of mixing ratios of products formed in the reaction H + *Cc*-GA. (a) H-deficient experiment: $[H]_0/[Cc\text{-GA}] \approx 2.3$ and $[Cc\text{-GA}]_0 = 143.4$ ppm for the reaction H + *Cc*-GA. (b) H-rich experiment: $[H]_0/[Cc\text{-GA}] \approx 7.5$ and $[Cc\text{-GA}]_0 = 203.9$ ppm for the reaction H + *Cc*-GA. $[H]_0$ was estimated from $[HCl]_0$. The regions shaded with blue and red correspond to the period of 380-nm and IR irradiation, respectively.

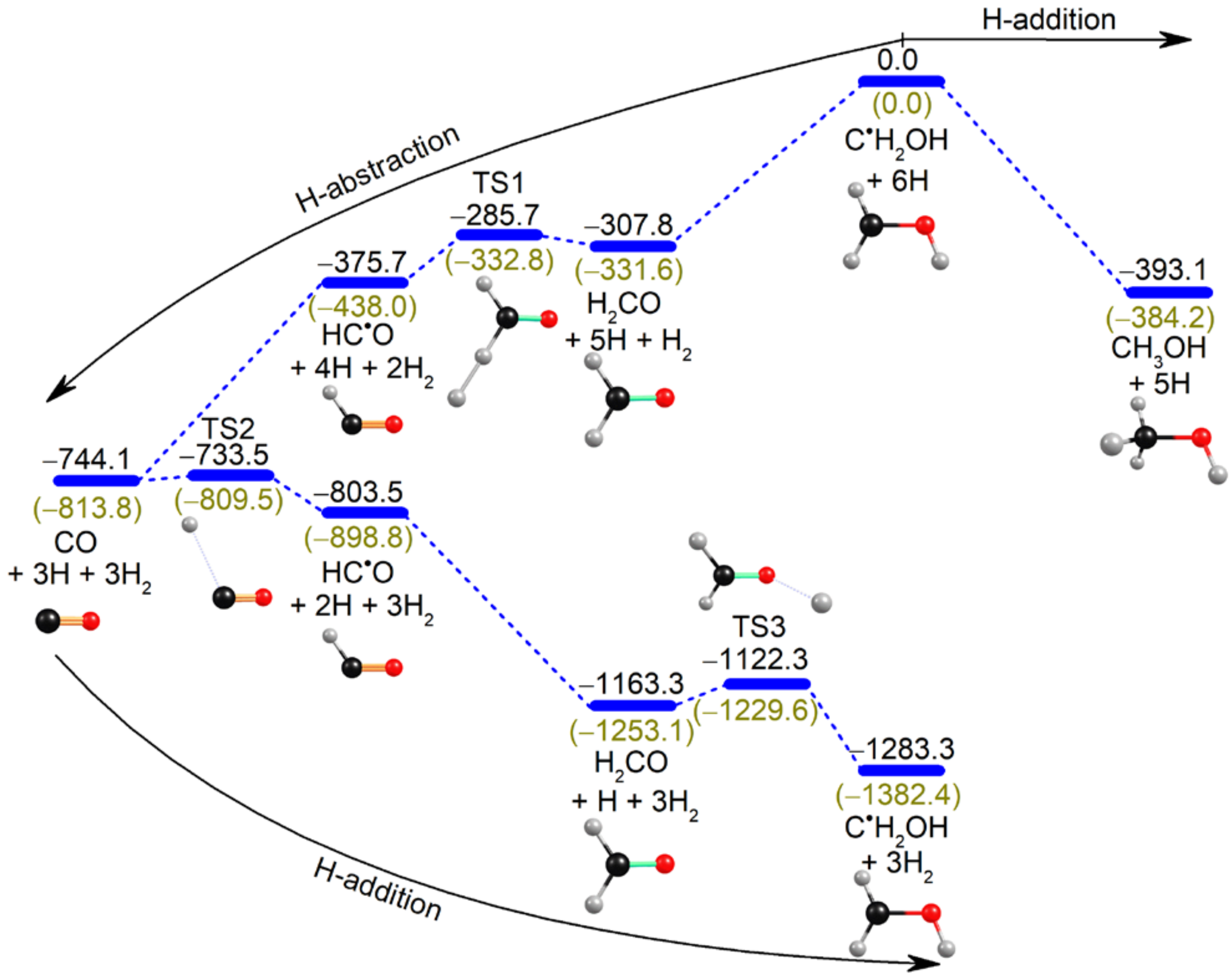

Figure S19. Potential-energy scheme of H-addition and H-abstraction reactions connecting $C^{\bullet}H_2OH$, H_2CO, $HC^{\bullet}O$, and CO. The energies (in kJ mol^{-1}) relative to $C^{\bullet}H_2OH$ + 6H were calculated with the CCSD(T)/aug-cc-pVTZ//B3LYP/aug-cc-pVTZ method (black); values in parentheses (olive) were calculated with the B3LYP/aug-cc-pVTZ method. Zero-point vibrational energies (ZPVE), calculated with the B3LYP/ aug-cc-pVTZ method, were corrected.

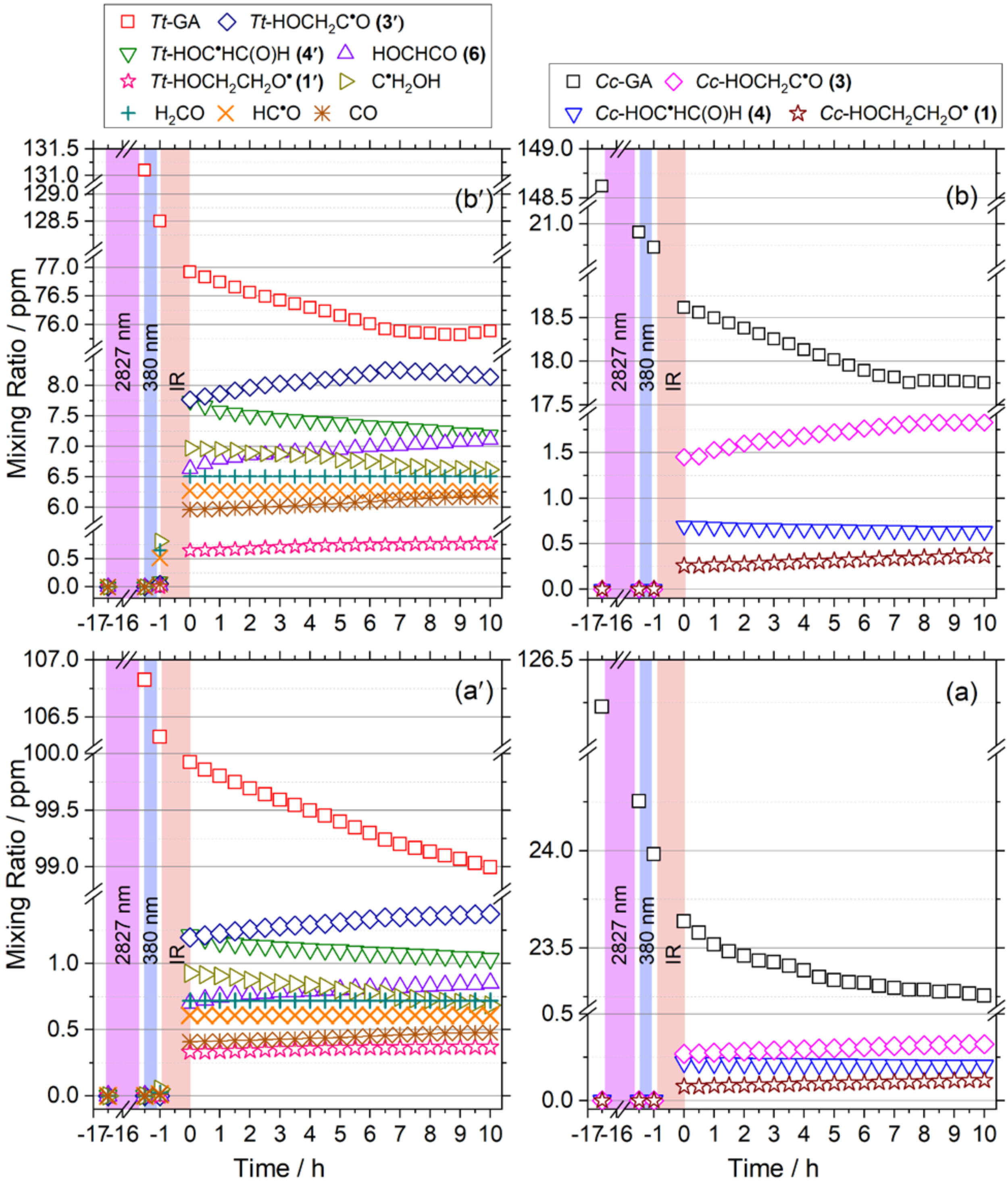

Figure S20. Temporal evolution of mixing ratios of *Tt*-GA and products formed in the reaction H + *Tt*-GA. Left panels represent temporal profiles of products from the reaction H + *Tt*-GA, whereas right panels represent temporal profiles of products from the reaction H + *Cc*-GA; *Tt*-GA was produced mainly after prolonged IR irradiation at 2827 nm. (a′) H-deficient experiment: $[H]_0/[Tt\text{-GA}] \approx 1.1$ and $[Tt\text{-GA}]_0 = 107$ ppm for the reaction H + *Tt*-GA. (a) H-deficient experiment for the H + *Cc*-GA. (b′) H-rich experiment: $[H]_0/[Tt\text{-GA}] \approx 4.1$ and $[Tt\text{-GA}]_0 = 131$ ppm for the reaction H + *Cc*-GA. (b) H-rich experiment for the H + *Cc*-GA. $[H]_0$ was estimated from $[HCl]_0$. The profile of *Tt*-$HOCH_2C^{\bullet}O$ **(3′)** in (b) was shifted up by 0.5 ppm for clarity. The regions shaded with pink, blue, and red correspond to the period of irradiations at 2827 nm, 380 nm, and IR, respectively.

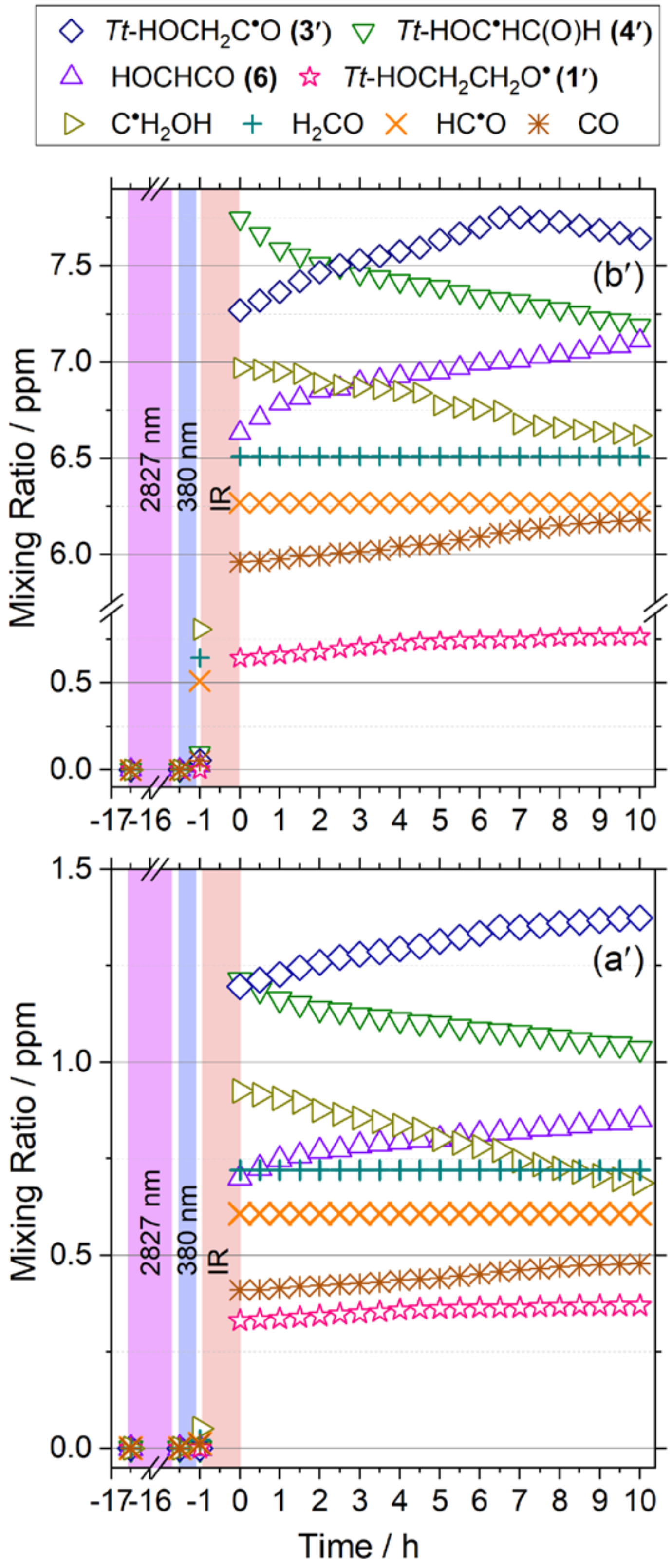

Figure 21. Expanded version of traces (a′) and (b′) of Figure 20: Temporal evolution of mixing ratios of products formed in the reaction H + *Tt*-GA. (a′) H-deficient experiment: $[H]_0/[Tt\text{-GA}] \approx 1.1$ and $[Tt\text{-GA}]_0 = 107$ ppm. (b′) $[H]_0/[Tt\text{-GA}] \approx 4.1$ and $[Tt\text{-GA}]_0 = 131$ ppm. $[H]_0$ was estimated from $[HCl]_0$. The regions shaded with pink, blue, and red correspond to the period of irradiations at 2827 nm, 380 nm, and IR, respectively.

Supplementary References

[1] Lee, Y.-F.; Chou, W.-T.; Johnson, B. A.; Tabor, D. P.; Sibert III, E. L.; Lee, Y.-P. Infrared Absorption of CH_3O and CD_3O Radicals Isolated in Solid *para*-H_2. *J. Mol. Spectrosc.* **2015**, *310*, 57−67. DOI: 10.1016/j.jms.2014.11.008

[2] Chin, W.; Chevalier, M.; Thon, R.; Pollet, R.; Ceponkus, J.; Crépin, C. Photochemistry of Glycolaldehyde in Cryogenic Matrices. *J. Chem. Phys.* **2014**, *140*, 224319. DOI: 10.1063/1.4881605

[3] Paulson, L. O.; Mutunga, F. M.; Follett, S. E.; Anderson D. T. Reactions of Atomic Hydrogen with Formic Acid and Carbon Monoxide in a Quantum Solid I: Anomalous Effect of Temperature. *J. Phys. Chem. A* **2014**, *118*, 7640−7652. DOI: 10.1021/jp502470j

[4] Tam, S. & Fajardo, M. Single and Double Infrared Transitions in Rapid-Vapor-Deposited Parahydrogen Solids: Application to Sample Thickness Determination and Quantitative Infrared Absorption Spectroscopy. *Appl. Spectrosc.* **2001**, *55*, 1634−1644. DOI: 10.1366/0003702011953946

[5] Silvera, I. F. The Solid Molecular Hydrogens in the Condensed Phase: Fundamentals and Static Properties. *Rev. Mod. Phys.* **1980**, *52*, 393–452. DOI: 10.1103/RevModPhys.52.393

[6] Fajardo, M. E. Matrix Isolation Spectroscopy in Solid Parahydrogen: A Primer. In Physics and Chemistry at Low Temperatures; Khriachtchev, L., Ed.; Pan Stanford Publishing, 2011. DOI: 10.1201/9780429066276-6

[7] Kettwich, S.C.; Raston, P. L.; Anderson, D. T. The Cl + H_2 →HCl + H Reaction Induced by IR + UV Irradiation of Cl_2 in Solid *para*-H_2: Experiment. *J. Phys. Chem. A* **2009**, *113*, 7621–7629. DOI: 10.1021/jp811206a

[8] Álvarez-Barcia, S.; Russ, P.; Kästner, J.; Lamberts, T. Hydrogen Transfer Reactions of Interstellar Complex Organic Molecules. *Mon. Not. R. Astron. Soc.* **2018**, *479*, 2007−2015. DOI: 10.1093/mnras/sty1478
